# FRET Assays for the Identification of *C. albicans* HSP90-Sba1 and *Human* HSP90α-p23 Binding Inhibitors

**DOI:** 10.3390/ph17040516

**Published:** 2024-04-17

**Authors:** Philip Kohlmann, Sergey N. Krylov, Pascal Marchand, Joachim Jose

**Affiliations:** 1 Institute of Pharmaceutical and Medicinal Chemistry, Pharmacampus, University of Münster, 48149 Münster, Germany; pkohlman@uni-muenster.de; 2 Department of Chemistry, York University, Toronto, ON M3J 1P3, Canada; skrylov@yorku.ca; 3 Centre for Research on Biomolecular Interactions, York University, Toronto, ON M3J 1P3, Canada; 4 Cibles et Médicaments des Infections et de l’Immunité, IICiMed, Nantes Université, UR 1155, F-44000 Nantes, France; pascal.marchand@univ-nantes.fr

**Keywords:** HSP90, Sba1, p23, drug discovery, accuracy assessment of *K*_d_, fungal infections, cancer, high-throughput screening (HTS) capability, FRET

## Abstract

Heat shock protein 90 (HSP90) is a critical target for anticancer and anti-fungal-infection therapies due to its central role as a molecular chaperone involved in protein folding and activation. In this study, we developed *in vitro* Förster Resonance Energy Transfer (FRET) assays to characterize the binding of *C. albicans* HSP90 to its co-chaperone Sba1, as well as that of the homologous *human* HSP90α to p23. The assay for *human* HSP90α binding to p23 enables selectivity assessment for compounds aimed to inhibit the binding of *C. albicans* HSP90 to Sba1 without affecting the physiological activity of *human* HSP90α. The combination of the two assays is important for antifungal drug development, while the assay for *human* HSP90α can potentially be used on its own for anticancer drug discovery. Since ATP binding of HSP90 is a prerequisite for HSP90-Sba1/p23 binding, ATP-competitive inhibitors can be identified with the assays. The specificity of binding of fusion protein constructs—HSP90-mNeonGreen (donor) and Sba1-mScarlet-I (acceptor)—to each other in our assay was confirmed via competitive inhibition by both non-labeled Sba1 and known ATP-competitive inhibitors. We utilized the developed assays to characterize the stability of both HSP90–Sba1 and HSP90α–p23 affinity complexes quantitatively. *K*_d_ values were determined and assessed for their precision and accuracy using the 95.5% confidence level. For HSP90-Sba1, the precision confidence interval (PCI) was found to be 70–120 (100 ± 20) nM while the accuracy confidence interval (ACI) was 100–130 nM. For HSP90α-p23, PCI was 180–260 (220 ± 40) nM and ACI was 200–270 nM. The developed assays were used to screen a nucleoside-mimetics library of 320 compounds for inhibitory activity against both *C. albicans* HSP90-Sba1 and *human* HSP90α-p23 binding. No novel active compounds were identified. Overall, the developed assays exhibited low data variability and robust signal separation, achieving *Z* factors > 0.5.

## 1. Introduction

Heat shock protein 90 (HSP90) belongs to a family of molecular chaperones protecting proteins from cellular stress and is essential for the viability of eukaryotes [1]. HSP90 forms homodimers and has an ATPase activity that drives distinct conformational changes necessary for its chaperone function. Dependent on the binding and hydrolysis of ATP, HSP90 traverses a conformational cycle further influenced by forming a complex with other proteins termed co-chaperones.

Structurally, the HSP90 monomer can be divided into three domains: an N-terminal domain (NTD) that encompasses an ATP-binding site involved in ATP hydrolysis and can transiently dimerize upon ATP association, connected by a charged flexible linker to the middle domain (MD) that is involved in many client protein interactions, and a C-terminal domain (CTD) that is inherently associated with HSP90’s dimerization. The CTD is also considered to contain a second nucleotide-binding site [2,3]. HSP90 is involved in a plethora of interactions with client proteins and co-chaperones. A maintained list of HSP90 interactors can be accessed via the Picard lab [4]. Details about the HSP90 chaperoning machinery have been discussed before in several reviews [1,5,6].

In *Homo sapiens*, HSP90 can be found in four isoforms that are associated with different cell compartments and are termed HSP90α and β, Grp94 and TRAP-1 [7]. The HSP90α and β isoforms primarily occur in the cytoplasm. HSP90α expression can be induced, whereas the β isoform is constitutively expressed. Grp94 is the isoform that is found in the endoplasmic reticulum, and TRAP-1 is present in mitochondria.

During HSP90’s conformational cycle, the chaperone associates and disassociates with a variety of co-chaperones. Upon ATP binding, HSP90 adopts a dimer conformation by the N terminus. This closed, rather twisted state of the dimer permits the binding of co-chaperones like p23 (or its homolog Sba1 in *S. cerevisiae* and *Candida albicans*) and Aha1. Whereas p23/Sba1 binding leads to a decrease in HSP90 ATPase activity, the binding of Aha1 accelerates it (Aha1 = Activator of HSP90 ATPase 1) [8]. Due to its central role as a ‘foldase’ and ‘holdase’ at the intersection of many signaling pathways, HSP90 and its interactions with co-chaperones are of relevance in cancer and neurodegenerative diseases [9,10,11,12]. Co-chaperones of HSP90, such as p23, have been shown to play a role in carcinogenesis [13,14].

Apart from that, HSP90 and co-chaperones like Aha1 and Sba1 also play a role in conferring drug resistance and virulence in fungal infections [15,16]. Thus, HSP90 has long been considered a promising drug target. It is strongly conserved across isoforms and different species, whereas co-chaperones such as p23 and Sba1 are not. Therefore, targeting these protein–protein interactions (PPIs) for the development of species-selective inhibitors designated, e.g., for antifungal treatment seems to be a promising approach. An increase in drug resistance of pathogenic fungi and a growing immunocompromised patient population at risk of fungal infections emphasize the need for novel anti-infective drugs [17], particularly antimycotic drugs with high selectivity [18]. Recently, the WHO has recognized *C. albicans* as a pathogen of critical concern and encouraged research on and development of antifungal medicines [19]. Following this need, there have been investigations on whether already existing drugs can be repurposed for the treatment of fungal infections [20].

Here, we report *in vitro* FRET assays for the identification and profiling of *C. albicans* HSP90-Sba1 binding inhibitors as well as for the study of the homologous binding of *human* HSP90α-p23. Since ATP binding of HSP90 is a prerequisite for HSP90-Sba1/p23 binding, ATP-competitive inhibition of HSP90 can be assessed using the assays. The assays can potentially be used to identify either new selective antifungal agents (by identifying active compounds with the *C. albicans* HSP90-Sba1 binding assay and assessing selectivity with the *human* HSP90α-p23 binding assay) or anticancer compounds (by using the *human* HSP90α-p23 binding assay).

## 2. Results

### 2.1. Development of FRET Assays to Characterize Fungal HSP90–Sba1 and Human HSP90α–p23 Binding

To enable the use of Förster Resonance Energy Transfer (FRET) for the quantification and characterization of *C. albicans* HSP90 binding to its co-chaperone Sba1 and homologous *human* HSP90α binding to p23, we generated fusion constructs of these proteins of interest with fluorescent proteins. The fluorescent proteins mNeonGreen and mScarlet-I were used as donor and acceptor fluorophores, respectively. They have been proven to be a suitable FRET pair for profiling inter-molecular binding [21]. Fusion proteins of *C. albicans* HSP90-mNeonGreen and *human* HSP90α-mNeonGreen were used as FRET donors. Sba1-mScarlet-I (*C. albicans*) and p23-mScarlet-I (*human* homolog) served as FRET acceptors. Proteins were expressed in *E. coli* and purified via ion metal affinity chromatography (IMAC) and size exclusion chromatography (SEC) (see Section 4.2).

**Figure 1:**
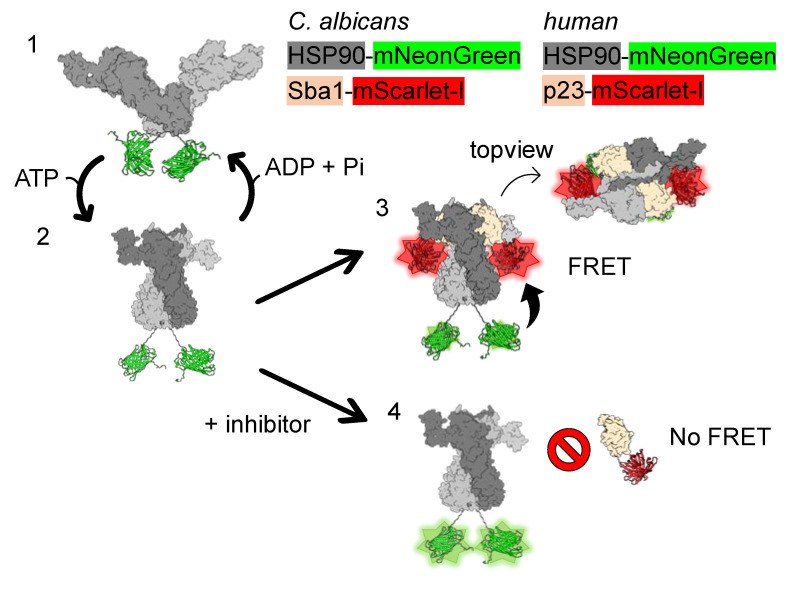
FRET assay setup for *C. albicans* HSP90–Sba1 or *human* HSP90α–p23 inhibitor screening. The FRET assay setup is explained for *C. albicans* HSP90–Sba1 binding. The corresponding assay setup for the *human* complex formation is analogous. (**1**) HSP90-mNeonGreen fusion proteins form homodimers that are dimerized at the C-terminal domain. HSP90-mNeonGreen adopts an open conformation in the absence of ATP. (**2**) Upon ATP binding, HSP90-mNeonGreen can progress to an N-terminally dimerized state. (**3**) The ATP-bound twisted configuration of HSP90-mNeonGreen enables the binding of Sba1-mScarlet-I to HSP90-mNeonGreen. Two Sba1 molecules can bind per HSP90 dimer. During this complex formation, the donor and acceptor come into close contact. This state allows FRET between the donor fluorescent protein mNeonGreen and the acceptor fluorescent protein mScarlet-I, resulting in an increase in FRET emission. Concomitantly, as energy is transferred from the donor fluorophore to the acceptor fluorophore via FRET, the donor fluorescence decreases compared to non-binding samples. Binding of Sba1 to HSP90 stabilizes the ATP-bound conformation of HSP90 leading to a deceleration of HSP90 ATPase function and its conformational cycle progression. Subsequent to ATP hydrolysis, HSP90 adopts the open conformation again (**1**), and Sba1 dissociates from the complex. (**4**) When an inhibitor of HSP90–Sba1 binding is added to this setup, no FRET occurs, resulting in a low sensitized emission as well as no reduction in fluorescence emission. Since the ATP-bound conformation of HSP90 is a prerequisite for HSP90–Sba1 binding, the assay is suitable for the identification of HSP90–Sba1 protein–protein interaction (PPI) inhibitors, as well as for the identification of ATP-competitive HSP90 inhibitors. HSP90 monomers are depicted in dark gray and gray. Fluorescent protein mNeonGreen is depicted in lime. Sba1 is shown in beige. Fluorescent protein mScarlet-I is colored red. Homology models of *C. albicans* HSP90 open and closed conformations (based on PDB IDs 2IOQ and 2CG9, respectively) and HSP90-Sba1 complex (based on PDB ID 2CG9) were created using the Swiss Model server [22]. The figure was created with ChimeraX 1.7.1 [23].

FRET is highly dependent on the distance between donor and acceptor fluorophores. When Sba1-mScarlet-I binds to HSP90-mNeonGreen, the fluorescent proteins are brought in close proximity, so that FRET from the excited donor molecule mNeonGreen to the acceptor molecule mScarlet-I can occur (Figure 1). Subsequently, the emission of the acceptor fluorophore, also called sensitized emission, increases relative to the case when there is no binding between the proteins of interest. From fluorescence measurements of three channels, FRET emission (*Em*_FRET_) can be derived (see Section 4.3). Additionally, as energy from the excited donor molecule is transferred to the acceptor fluorophore, mNeonGreen fluorescence emission (*FL*_DD_) is decreased. The same principle applies to *human* HSP90α-mNeonGreen binding to p23-mScarlet-I.

### 2.2. K_d_ Determination for C. albicans HSP90–Sba1 and Human HSP90α–p23 Binding

**Figure 2:**
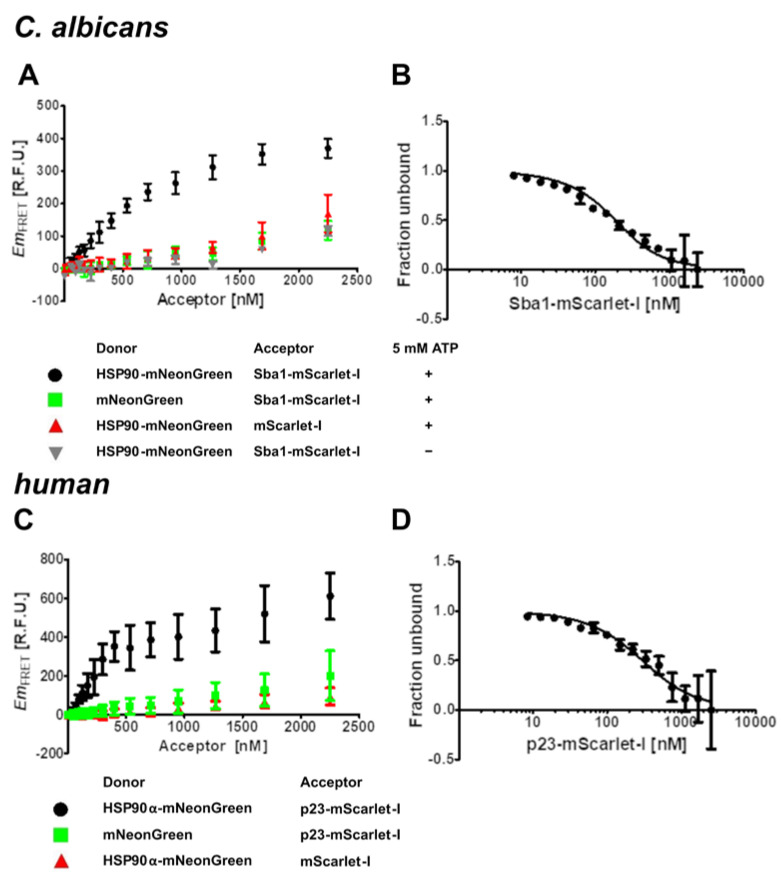
Specificity and equilibrium dissociation constant (*K*_d_) determination of *C. albicans* HSP90–Sba1 and *human* HSP90α–p23 binding via FRET. (**A**,**C**) All donor concentrations were kept constant at 1 µM. The acceptor was varied in a concentration range of 0–2250 nM (lowest non-zero concentration 53 nM). (**A**) Specificity of *C. albicans* HSP90-mNeonGreen binding to Sba1-mScarlet-I in comparison with donor control and acceptor control. When omitting ATP from the reaction buffer (grey down-pointing triangles), HSP90-mNeonGreen–Sba1-mScarlet-I binding is abrogated. mNeonGreen (green squares) and mScarlet-I (red up-pointing triangles) show a linear, unspecific rise in FRET emission. (**B**) The *C. albicans* HSP90E36A-mNeonGreen concentration was kept constant at 200 nM. Sba1-mScarlet-I concentration was varied from 0–3000 nM (lowest non-zero concentration 10 nM). The samples were incubated at 37 °C for 3 h to ensure equilibrium. The determined *K*_d_ value was 100 nM (PCI: 80–120 nM, ACI: 100–140 nM). (**C**) Shown are the results for the *human* homologous complex formation of HSP90α–p23. (**D**) The experiment for determining the *K*_d_ of *human* HSP90αE47A-mNeonGreen–p23-mScarlet-I binding was performed analogously to (**B**) except for incubating at 37 °C for 3 h. The *K*_d_ value was 210 nM (PCI: 170–250 nM, ACI: 180–260 nM). *Em*_FRET_: FRET emission, R.F.U.: relative fluorescence units, PCI: precision confidence interval, ACI: accurate confidence interval. Both PCI and ACI were calculated at a 95.5% confidence level. Error bars represent the standard deviation. Experiments with varying incubation times to test for equilibration are included in the Supplementary Materials (Figure S1). Reports for ACI determination are included in the Supplementary Materials.

As a proof of principle for the FRET assays, we assessed their readout specificity (Figure 2). For the *C. albicans* interaction, the FRET signal of the binding sample (HSP90-mNeonGreen and Sba1-mScarlet-I) was compared to a donor control (replacing HSP90-mNeonGreen with mNeonGreen) and an acceptor control (replacing Sba1-mScarlet-I with mScarlet-I) (Figure 2A). Analogous experiments were conducted for *human* HSP90α-mNeonGreen binding to p23-mScarlet-I (Figure 2B). The donor concentration was fixed at 1 µM and the acceptor was varied in a concentration range from 0 to 2250 nM (lowest non-zero concentration 53 nM). For the HSP90 co-chaperone samples, we observed a saturation-like binding curve (black circles), whereas the donor (green squares) and acceptor (red triangles) controls showed both lower FRET emission and a linear increase in FRET emission that we attributed to unspecific effects, such as molecular crowding at high acceptor concentrations, also termed stochastic FRET [24,25].

Furthermore, an important prerequisite for the binding of *C. albicans* HSP90 to Sba1 (and its *human* homologous interaction) is that HSP90 is in a transient complex with ATP. The conformational cycle of HSP90 is driven by the binding and hydrolysis of ATP [1]. Upon binding of ATP to HSP90, HSP90 can adopt a twisted closed conformation, in which alongside the C-terminal domains of the HSP90 dimer, the N-terminal domains are also transiently dimerized. This conformational state allows the binding of Sba1/p23 to HSP90. If in contrast to the other samples (containing 5 mM ATP), ATP is omitted from the buffer, no binding of HSP90-mNeonGreen to Sba1-mScarlet-I can be observed (Figure 2A, gray triangles). The resulting FRET signal is congruent with the negative donor and acceptor controls. The strong ATP dependence of the binding is in agreement with previous reports of the HSP90–Sba1/p23 binding from *S. cerevisiae* and *human* protein homologs [26].

Binding isotherms obtained from modified experiments were used for the determination of equilibrium dissociation constant (*K*_d_) values by nonlinear regression. Large systematic errors in *K*_d_ values can occur when the ratio between the concentration of the constant component (*L*_0_) and *K*_d_ is high [27]. Such errors can be minimized by using *L*_0_ (here either *C. albicans* HSP90-mNeonGreen or *human* HSP90α-mNeonGreen) at the level of the limit of quantitation (LOQ) [27,28,29]. Furthermore, the attainment of the equilibrium in the investigated binding reaction must be assessed [28]. The binding of HSP90 to Sba1/p23 in *C. albicans* and *humans* is influenced by the conformational change of HSP90 caused by ATP binding to HSP90. However, ATP is also hydrolyzed over time by HSP90. To safeguard the achievement of equilibrium in the binding of HSP90 to Sba1/p23 from effects of ATP hydrolysis, HSP90 mutants defective in ATP-hydrolysis were generated for *C. albicans* HSP90 and *human* HSP90α and used for *K*_d_ determination experiments. Mutation of a glutamic acid to alanine in the N-terminal nucleotide-binding site of HSP90 (E36A in *C. albicans* HSP90 and E47A in *human* HSP90α) has been shown to result in the abrogation of ATP hydrolysis, while only minimally affecting ATP binding [30,31,32].

Thus, for *K*_d_ determination of the *C. albicans* HSP90–Sba1 complex, HSP90E36A-mNeonGreen (200 nM) was incubated with various concentrations of Sba1-mScarlet-I (0-3000 nM, 10 nM lowest non-zero concentration) at 30 °C in reaction buffer containing 5 mM ATP. To check for equilibrium, we conducted FRET measurements every 60 min for up to 4 h (see Figure S1). The experiments for *K*_d_ determination of *human* HSP90α–p23 binding were performed similarly, except for incubation at 37 °C instead of 30 °C. Shown in Figure 2B,D are results for measurements after 180 min of incubation, at which equilibrium was established. From the binding isotherms, a *K*_d_ value can be derived (Figure 2B,D).

Since *K*_d_ is computed by nonlinear regression from a binding isotherm and depends on concentration inputs of both a limiting component (*L*_0_) and a varied component (*T*_0_, here either *C. albicans* Sba1-mScarlet-I or *human* p23-mScarlet-I concentration), errors in both *L*_0_ and *T*_0_ can have a severe impact on the calculated *K*_d_ values (see also Section 4.3.1 Equation (4)) [27,29]. These ramifications of systematic errors (arising from normally distributed random errors) in *L*_0_ and *T*_0_ should be reported to attain an informative range of *K*_d_. A detailed description of how the accuracy confidence interval (ACI) of the *K*_d_ value can be determined can be found elsewhere [29]. Reports for ACI determination are included in the Supplementary Materials. We calculated the ACI and error of the fit (called precision confidence interval (PCI) hereafter) at a 99.5% confidence level.

The calculated *K*_d_ value of 100 nM (PCI: ± 20 nM (70–120 nM), ACI: 100–130 nM) for *C. albicans* HSP90–Sba1 binding differs by a factor of ~5–18 from previously reported results of 540 ± 80 nM and 1750 ± 130 nM of closely related *S. cerevisiae* HSP90–Sba1 binding determined by SPR and ITC [8,33]. Both *C. albicans* and *S. cerevisiae* belong to the yeast family. The determined *K*_d_ for *human* HSP90α–p23 binding was 220 nM (PCI: 180–260 nM, ACI: 200–270 nM). The affinity of the *human* HSP90 isoform alpha to p23 seems to be similar to the *C. albicans* orthologous complex formation, as both *K*_d_ values lie in the same range (compare Figure 2B,D). A *K*_d_ value for the *human* HSP90β–p23 complex was reported to be 1500 nM [34], differing by a factor of ~7 from our results for HSP90α–p23 binding.

In general, since affinity and subsequently *K*_d_ also depend on temperature and buffer conditions, a comparison of equilibrium dissociation constants with the literature is at times challenging. Reporting PCI as well as ACI informs researchers about the impact of *L*_0_ and random errors of *L*_0_ and *T*_0_ on determined *K*_d_. Thus, researchers can make an informed decision after only a single binding experiment if their experiment should be modified (in particular by lowering *L*_0_) to improve the accuracy of their determined *K*_d_. Furthermore, the declaration of PCI and ACI when reporting *K*_d_ should improve the comparability of the data with values from other studies regarding this issue. However, caution is advised in removing all sources of possible systematic errors and ensuring saturation in binding reactions and reach of equilibrium when conducting experiments to generate high-quality data. A limitation of the developed assays for *K*_d_ determination is the occurrence of high unspecific FRET at high acceptor concentrations, presumably due to crowding effects. This limits the dynamic concentration range, more specifically the upper concentration limit of the acceptor important for reaching saturation in the binding isotherm.

### 2.3. Influence of Unlabeled Sba1 on HSP90-mNeonGreen Binding to Sba1-mScarlet-I and Unlabeled p23 on Binding of HSP90α-mNeonGreen to p23-mScarlet-I

To assess the capability of the FRET assays to identify and characterize direct PPI HSP90-Sba1/p23 inhibitors, we conducted a set of experiments. The experiments will be described for *C. albicans* HSP90–Sba1 binding. The experiments for *human* HSP90α–p23 binding were conducted analogously.

**Figure 3:**
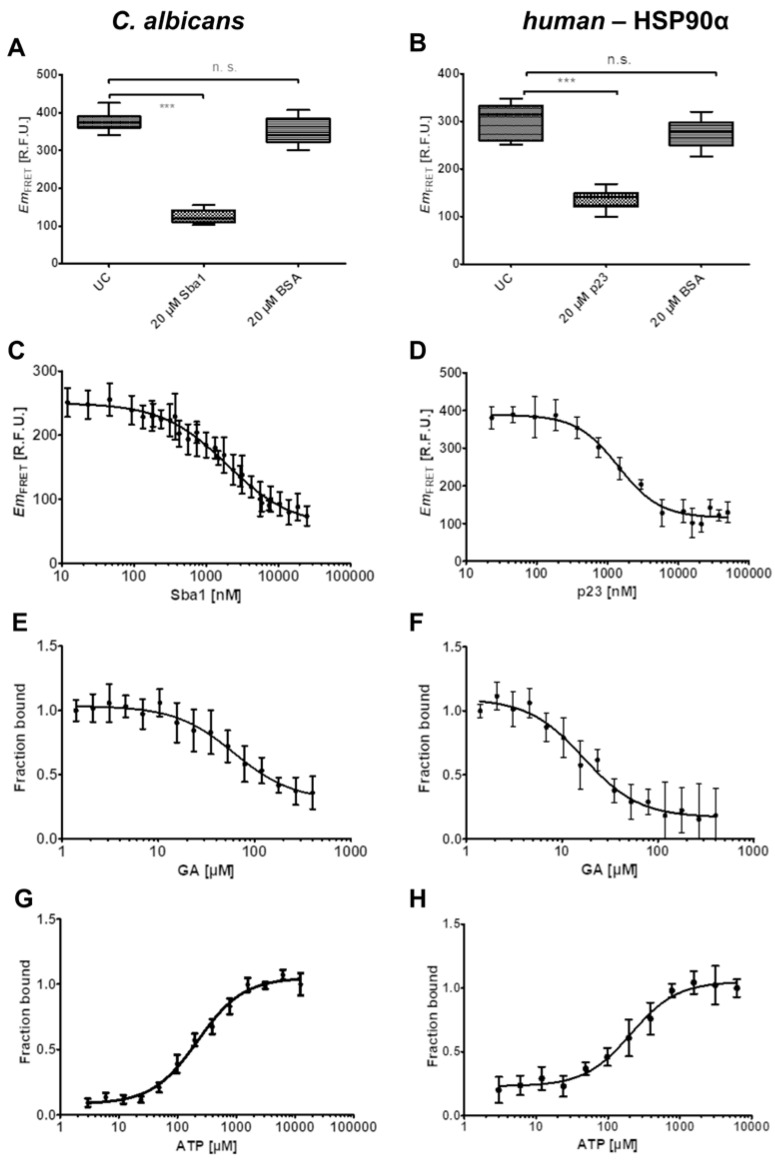
Characterization of model *C. albicans* HSP90–Sba1 and *human* HSP90α–p23 binding inhibitors via FRET. (**A**) Sba1 selectively competes with Sba1-mScarlet-I for binding to HSP90-mNeonGreen. HSP90-mNeonGreen (1 µM) and Sba1-mScarlet-I (2 µM) were incubated in reaction buffer containing 5 mM ATP. The addition of Sba1 (20 µM) showed a significant reduction (*p* < 0.001, depicted as ***) in the observed *Em*_FRET_ in comparison to the untreated control (UC). When adding bovine serum albumin (BSA) (20 µM) to the aforementioned constant concentrations of HSP90-mNeonGreen/Sba1-mScarlet-I, there was no significant (n.s.) reduction in *Em*_FRET_. (**B**) p23 selectively competes with p23-mScarlet-I for binding to HSP90α-mNeonGreen. The experiment was performed analogously to (**A**). (**C**) Sba1 competes with Sba1-mScarlet-I for binding to HSP90-mNeonGreen in a dose-dependent manner. HSP90-mNeonGreen (1 µM) and Sba1-mScarlet-I (1 µM) were incubated in reaction buffer containing 5 mM ATP. The determined *IC*_50_ is 1950 ± 230 nM. (**D**) p23 competes with p23-mScarlet-I for binding to HSP90α-mNeonGreen in a dose-dependent manner. The determined *IC*_50_ is 1420 ± 190 nM. The experiment was performed analogously to (**C**). (**E**) Small molecule HSP90 inhibitor geldanamycin (GA) disrupts the binding of Sba1-mScarlet-I to the ATP-hydrolysis-defective mutant HSP90E36A-mNeonGreen in a dose-dependent manner. HSP90E36A-mNeonGreen (1 µM) and Sba1-mScarlet-I (2 µM) were incubated in reaction buffer containing 5 mM ATP. The geldanamycin concentration was varied in a range of 0–400 µM (lowest non-zero concentration 1.4 µM). The determined *IC*_50_ is 60 ± 10 µM. (**F**) GA disrupts the binding of p23-mScarlet-I and the ATP-hydrolysis-defective mutant HSP90αE47A-mNeonGreen in a dose-dependent manner. HSP90αE47A-mNeonGreen (1 µM) and p23-mScarlet-I (2 µM) were incubated at 37 °C for 15 min in reaction buffer containing 5 mM ATP. The determined *IC*_50_ is 17 ± 3 µM. (**G**) ATP concentration shows a strong influence on HSP90E36A-mNeonGreen–Sba1-mScarlet-I binding. HSP90E36A-mNeonGreen (1 µM) was incubated with Sba1-mScarlet-I (2 µM) in reaction buffer. ATP was varied in a concentration range of 0–12,500 µM (lowest non-zero concentration 3 µM). The *EC*_50_ is 220 ± 40 µM. (**H**) ATP concentration shows a strong influence on HSP90αE47A-mNeonGreen–p23-mScarlet-I binding. HSP90αE47A-mNeonGreen (1 µM) was incubated with p23-mScarlet-I (2 µM) in reaction buffer. The *EC*_50_ is 210 ± 30 µM. Error bars represent the standard deviation. Given error is the error of the fit. *Em*_FRET_ [R.F.U.]: FRET emission in relative fluorescence units. (**E**–**H**) Maximum *Em*_FRET_ was normalized to 1 to represent the full binding of HSP90 and co-chaperone.

First, the specificity of unlabeled Sba1 to compete with Sba1-mScarlet-I for binding to HSP90-mNeonGreen was investigated (Figure 3A). In this investigation, 1 µM HSP90-mNeonGreen was incubated with 2 µM Sba1-mScarlet-I in reaction buffer containing 5 mM ATP either alone (UC) or with the addition of 20 µM Sba1. As a negative control, samples containing 20 µM bovine serum albumin (BSA) were used. BSA should not interfere with the binding of HSP90-mNeonGreen to Sba1-mScarlet-I. The addition of unlabeled Sba1 or p23 to respective fluorescent fusion protein binding pairs showed a significant reduction in FRET emission (*p* < 0.001, one-way ANOVA with post hoc Dunnett’s test) (Figure 3A,B). In contrast, BSA samples did not show a significant influence on the complex formation.

Furthermore, a dose-dependent reduction in FRET emission was observed when varying concentrations of unlabeled Sba1 were incubated with fixed concentrations of the donor and acceptor (both 1 µM). The relative *IC*_50_ value for the inhibition of *C. albicans* HSP90-mNeonGreen–Sba1-mScarlet-I binding by unlabeled Sba1 was 1.95 ± 0.23 µM (Figure 2C). The *K*_i_ value derived from the competition of Sba1 with Sba1-mScarlet-I for binding to HSP90-mNeonGreen was 100 ± 10 nM. The *K*_d_ value of the direct interaction was in a similar range (100 nM, PCI: 70–120 nM, ACI: 100–130 nM) (Figure 2B), indicating that the fluorescent protein mScarlet-I does not affect binding affinity to HSP90-mNeonGreen. For the inhibition of unlabeled p23 of *human* HSP90α-mNeonGreen–p23-mScarlet-I binding, a relative *IC*_50_ of 1.4 ± 0.2 µM was determined (Figure 3D). The corresponding *K*_i_ value for the *human* competition of p23 with p23-mScarlet-I for binding to HSP90α-mNeonGreen was 100 ± 20 nM. This suggests a similar conclusion, as the *K*_d_ value of the direct binding experiment was 220 nM (PCI: 180–260 nM, ACI: 200–270 nM) (Figure 2D).

### 2.4. Influence of Geldanamycin on the Binding of HSP90-Sba1 and HSP90α-p23

To confirm that ATP-competitive inhibitors of HSP90 can be characterized regarding their potency with the FRET assays, we determined the *IC*_50,rel_ of geldanamycin, a known HSP90 inhibitor for both *C. albicans* and *human* interactions. As ATP is required to obtain binding between HSP90 and the co-chaperone, we incubated 1 µM of the donor and 2 µM of the acceptor in the presence of 5 mM ATP. However, ATP is hydrolyzed by *wild-type* HSP90 in the course of the conformational cycle. To remove the uncertainty in the possible fluctuation of ATP concentration over time, in the experiments, HSP90E36A-mNeonGreen was used for *C. albicans,* and HSP90αE47A-mNeonGreen was for *human* interactions. Using these mutants enabled the determination of *IC*_50,rel_ values of 60 ± 10 µM for *C. albicans* HSP90–Sba1 binding inhibition and 17 ± 3 µM for the inhibition of *human* HSP90α–p23 binding.

### 2.5. Influence of ATP on HSP90-Sba1/p23 Binding and Optimization for Inhibitor Screening

Since ATP concentration strongly affects the PPI on the one hand but impedes the detection of HSP90 ATP-competitive substances on the other hand, we assessed the optimal ATP concentration for screening conditions. For this purpose, the ATP concentration should be as high as necessary to achieve maximum separation between binding and non-binding samples, and as low as possible to favor potential ATP-competitive compound detection. Variation in ATP (0–12,500 µM, lowest non-zero concentration 3 µM) in the presence of fixed concentrations of donor HSP90E36A-mNeonGreen (*C. albicans*) or HSP90αE47A-mNeonGreen (*human*) (1 µM) and acceptor Sba1-mScarlet-I (*C. albicans*) or p23-mScarlet-I (*human*) (2 µM) showed a dose-dependent increase in *Em*_FRET_ and by association HSP90–co-chaperone binding. Maximum binding was reached at ATP concentrations of ~3 mM. For the screening of compounds, we used this concentration in all subsequent experiments (see Section 2.6 and Section 2.7). Generally, the effects of ATP on the binding of *C. albicans* and the *human* HSP90α isoform with the co-chaperones seem to be similar, as both showed similar *EC*_50,rel_ values of 220 ± 40 µM and 210 ± 30 µM, respectively.

To assess if the ATP affinity of *C. albicans* HSP90 is close to that of other HSP90 species, we determined the binding of the hydrolysis-stable ATP analog AMP-PNP to *C. albicans* HSP90-mNeonGreen via microscale thermophoresis (MST) (see Supplementary Materials Figure S2). The resultant *K*_d_ value was 200 ± 70 µM (error of the fit), being in good agreement with published results from *yeast* HSP90 ATP and AMP-PNP binding experiments ranging from 30 µM to 132 ± 73 µM [26,34,35,36]. For AMP-PNP binding to *human* HSP90β at 25 °C, McLaughlin et al. reported a *K*_d_ value of 148 ± 12 µM [37]. For the *human* HSP90β–p23 complex, a *K*_d_ value of 39 ± 12 µM of AMP-PNP binding is reported [34]. Judging by the range and overall similarity to *K*_d_ values of ATP and AMP-PNP binding to *human*, *yeast* and *C. albicans* HSP90, the presence of p23 or Sba1 does not seem to strongly alter HSP90’s affinity to ATP. It can be speculated that based on this and the similarity of our *EC*_50_ values for ATP influence on both *C. albicans* and *human* HSP90–co-chaperone binding, the ATP affinity of the discussed HSP90 species is similar as well.

### 2.6. Validation of Screening Assay Conditions

**Figure 4:**
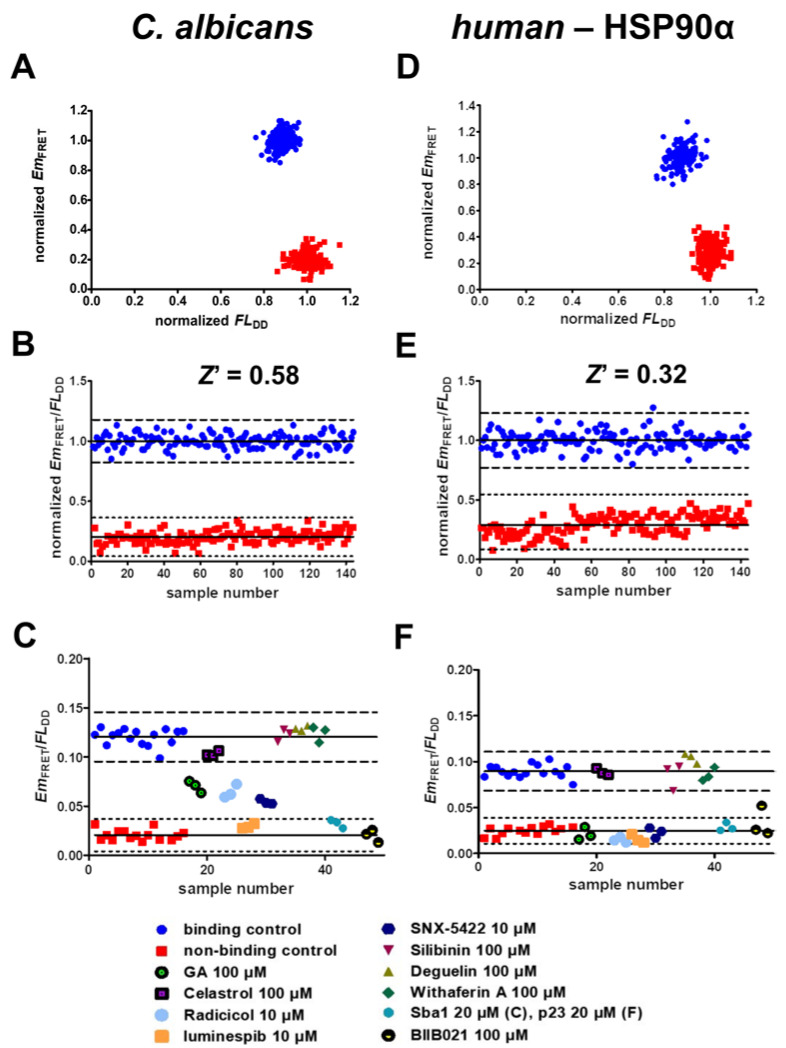
Categorization of screening assay quality and validation. (**A**) Binding control samples containing 3 mM ATP (blue circles) show high FRET emission (*Em*_FRET_) as well as a lower donor fluorescence (*FL*_DD_) compared to the non-binding control containing no ATP (red squares). *C. albicans* HSP90-mNeonGreen (1 µM) and Sba1-mScarlet-I (2 µM) in reaction buffer with 1% DMSO containing either 3 mM ATP or no ATP were incubated for 15 min at 30 °C prior to measurement. (**B**) Calculation of the quotient of FRET emission and donor emission (*Em*_FRET_/*FL*_DD_) results in robust separation and a *Z*′ factor of 0.58. (**C**) The screening assay can identify HSP90-Sba1 binding inhibitors with a high degree of confidence. HSP90-mNeonGreen (1 µM) and Sba1-mScarlet-I (2 µM) in reaction buffer, 3 mM ATP and 1% DMSO were incubated with various literature-described HSP90 inhibitors at concentrations of 10 or 100 µM for 15 min at 30 °C prior to measurement. When the hit threshold is defined as 3 SDs of the binding control mean (blue circles), ATP-competitive inhibitors of HSP90 geldanamycin, radicicol, luminespib (NVP-AUY922), SNX-5422 and BIIB021 are reliably identified as disrupting HSP90-Sba1 binding. Non-ATP competitive HSP90 inhibitors silibinin, deguelin and withaferin A do not show an effect on HSP90–Sba1 binding. Furthermore, Sba1 (20 µM) is also identified as disrupting HSP90-mNeonGreen–Sba1-mScarlet-I binding. (**D**–**F**) The assay conditions for *human* HSP90α-mNeonGreen–p23-mScarlet-I binding inhibitor identification are analogous to the *C. albicans* assay, with the exception that samples were incubated for 15 min at 37 °C prior to measurement. (**E**) For the *human* HSP90–p23 inhibitor screening assay, a *Z*′ factor of 0.32 was calculated. This classifies the assay as a double assay, indicating that compounds screened for HSP90α–p23 binding inhibition should be screened in duplicates. (**B**,**C**,**E**,**F**) Solid lines represent means of each data set (binding control or non-binding control). Dashed lines represent 3 standard deviations (SDs) from the respective mean.

We used the *Z*′ factor (*Z*′) to optimize the FRET assays for screening purposes [38]. *Z*′ represents a simple statistical parameter for the quality assessment and optimization of HTS assays and screening assays in general. It is a measure that determines the degree of signal separation between binding and non-binding data sets. Calculation of the *Z*′ factor is based on the standard deviation of the individual data sets as well as their general signal separation. Values ≥ 0.5 can be classified as excellent for screening purposes. Since binding of HSP90 to Sba1 in *C. albicans* and *human* HSP90α–p23 strongly depends on the prior binding of ATP to HSP90, all binding control (Figure 4A–F, blue circles) and tested compound samples contained 3 mM ATP in the buffer, whereas ATP was omitted in non-binding control samples (Figure 4A–F, red squares). To reliably identify active inhibitory compounds, an ideal assay generates a high signal separation between binding and non-binding control samples with low signal fluctuation of said data sets. When the fluorescent fusion proteins of HSP90 and co-chaperones bind to each other, FRET occurs between donor and acceptor fluorophores, and the sensitized emission (*Em*_FRET_) signal increases. Consequently, the fluorescence intensity of the donor decreases (*FL*_DD_). This phenomenon is shown in Figure 4A,D. Thus, to achieve a higher signal separation as well as lower fluctuation of binding and non-binding samples, we calculated the quotient of *Em*_FRET_ and *FL*_DD_ and used it as the readout signal to evaluate compounds and categorize them as inhibitors or non-active compounds. To obtain an informative *Z*′ value, we calculated it based on at least three independent experiments amounting to 144 samples for both binding and non-binding control data sets (Figure 4B,E). The resulting *Z*′ factor of the *C. albicans* HSP90–Sba1 binding inhibitor screening assay was 0.58. According to Zhang et al., this classifies the assay as excellent related to screening [39]. The calculated *Z*′ factor for the *human* HSP90α–p23 inhibitor screening assay was 0.32, classifying it as a double assay. This means that screening of compounds in duplicates is advisable to increase the reliability of the assay results. We defined the ‘hit threshold’ as a decrease in readout signal of more than 3 standard deviations (SDs) from the mean signal of the binding control. This constitutes a common threshold for hit identification in HTS campaigns [38,39].

To validate the capability of the assays to distinguish inhibitors of *C. albicans* HSP90–Sba1 and *human* HSP90α–p23 binding from HSP90 inhibitors showing no influence on the complex formation, we screened a set of literature-described HSP90 inhibitors in concentrations of 10 or 100 µM in triplicates (Figure 4C,F). The characteristics of the screened HSP90 inhibitors are detailed in Table S1. The results show that ATP-competitive inhibitors of HSP90 that bind to its N-terminal ATP pocket are reliably identified as hits, disrupting HSP90–co-chaperone binding in both the *C. albicans* and *human* homologous interactions. These encompass geldanamycin, radicicol, NVP-AUY922 (also known as luminespib), SNX-5422 and BIIB021. Also, unlabeled Sba1 (*C. albicans*) and p23 (*human*) are identified as hits, supporting that direct PPI inhibitors can also be identified under screening conditions. These compounds led to a low *Em*_FRET_, as no energy was transferred from the donor to the acceptor molecule. Hence, the donor fluorescence (*FL*_DD_) was not reduced and was thus higher than that of the binding control samples (blue circles, containing 3 mM ATP). Substances that are reported to bind to the C-terminal domain of HSP90 show no influence on HSP90–co-chaperone binding at screened concentrations. These substances include celastrol, silibinin, deguelin and withaferin A. Celastrol and withaferin A have been reported to not influence the HSP90–p23 interaction [40,41]. Our results come to the same conclusion for both the investigated *C. albicans* HSP90–Sba1 and the *human* HSP90α–p23 complex formation.

Another reported HSP90 inhibitor, the natural product (−)-Epigallocatechin-3-gallate (EGCG), was identified in our assays as a pan-assay interfering compound (PAIN) (see Figure S3) [42,43]. EGCG showed both a strong reduction in FRET emission as well as a strong reduction in donor emission, indicating that this effect is not due to disruption of the PPI, but rather due to general protein reactivity. EGCG contains two catecholic as well as pyrogallol (effectively a bis-catechol) structural motifs, both being recognized as PAIN motifs [44]. EGCG, among other catechol-containing compounds, has been found to have a variety of biological activities and to frequently signal in bioassays. Baell et al. also noted that those catechols were not optimizable and could interfere with bioassays via different mechanisms [44]. Consequently, EGCG is classified as a natural product PAIN. Our results confirm this interpretation.

### 2.7. Screening of Nucleoside-Mimetics Library

**Figure 5:**
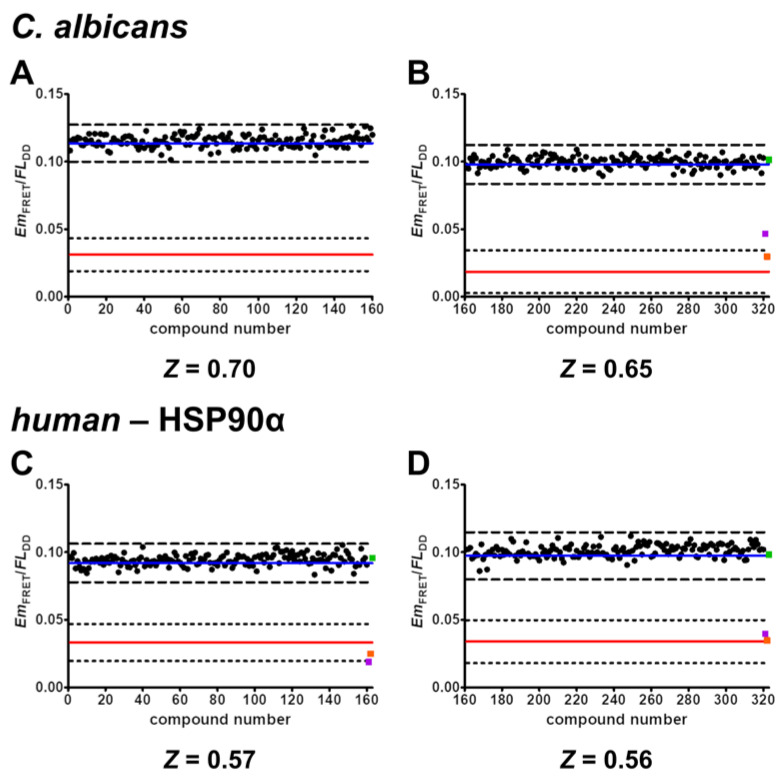
Screening of nucleoside-mimetics library. Compounds were screened in duplicates at a concentration of 100 µM. Shown in the graphs is the average *Em*_FRET_/*FL*_DD_ signal for each compound. The average of the binding control (containing 3 mM ATP) is shown as a solid blue line. The average of the non-binding control (no ATP) is shown as a solid red line. Averages of inhibition controls geldanamycin (100 µM) and NVP-AUY922 (10 µM) are depicted as purple and orange squares, respectively. Non-inhibition control withaferin A (10 µM) is pictured as a green square. Both inhibition and non-inhibition controls were screened analogously to compounds in duplicates. Dashed lines represent 3 standard deviations (SDs) from the respective mean (3 mM ATP/no ATP). (**A**–**D**) Each graph shows the screening results of one 384-well plate for either *C. albicans* HSP90–Sba1 binding inhibition (**A**,**B**) or *human* HSP90α–p23 binding inhibition (**C**,**D**) and the corresponding calculated Z factor. When the hit limit is defined as 3 SDs, none of the compounds can be identified as a hit. The *Z* factor was calculated using the average of the twice-screened compounds. Graphs with *Em*_FRET_/*FL*_DD_ signal for each well are included in the SM (Figure S4). A list with the structures of the screened compounds is included in the SM (Table S2).

In an attempt to find novel active pharmaceutical ingredients (APIs) that disrupt the binding of *C. albicans* HSP90–Sba1 or *human* HSP90α–p23, we screened a nucleoside-mimetics library of 320 compounds obtained from Enamine (NML-320) (Figure 5). The compounds in this targeted library have been filtered for undesired functionalities and reactive groups by Enamine. A list with the structures of these compounds is included in Table S2. Screening experiments were performed in duplicates for all compounds. This increased the robustness of the assays by reducing the signal fluctuation, leading to *Z* factors (*Z*) of 0.70 and 0.65 (calculated for each 384-well plate) for screening of *C. albicans* HSP90–Sba1 binding inhibitors and *Z* factors of 0.57 and 0.56 for *human* HSP90α–p23 binding inhibitors, respectively. When defining the hit threshold as a decrease in the mean signal of the binding control sample (represented by a blue solid line) by more than 3 SDs (depicted as dashed lines), no compound was a hit. This pertains to both *C. albicans* and *human* PPI. The premise of screening a nucleoside-mimetics library was to increase the chance of finding an API, since HSP90 has a well-defined ATP binding pocket in the N-terminal domain and a putative secondary ATP-binding site in the C-terminal domain [3]. Typically, the hit rate in many HTS campaigns is below 1% [40]. An HTS campaign by Rowlands et al. of ~56,000 compounds for inhibition of HSP90 ATPase reported a hit rate of 0.3% [45]. Though the chances of a focused library of nucleoside mimetics encompassing an API should presumably be higher, no active compound was identified.

### 2.8. Verification of C. albicans HSP90 Homodimers and HSP90–Sba1 Complex

**Figure 6:**
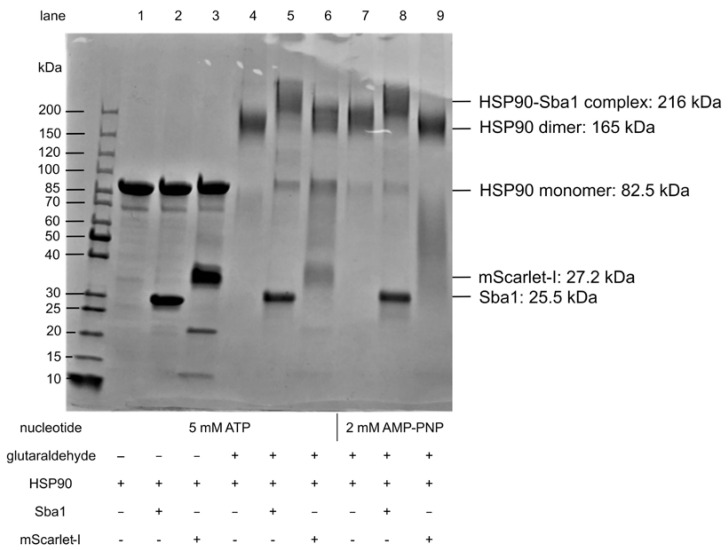
Cross-linking SDS-PAGE analysis of *C. albicans* HSP90 dimers and HSP90–Sba1 binding. HSP90 (10 µM) was incubated with an equimolar amount of Sba1 or mScarlet-I in the presence of either 5 mM ATP or 2 mM AMP-PNP as indicated. Cross-linking was induced by the addition of a 2.5% glutaraldehyde solution. Proteins were separated by SDS-PAGE without prior heating of samples followed by Coomassie staining. Lanes 1-3 not treated with glutaraldehyde show monomeric HSP90 (82.5 kDa) as well as Sba1 (25.5 kDa, lane 2) and mScarlet-I (27.2 kDa, lane 3). mScarlet-I was used as a negative control to show that cross-linking was specific for interacting proteins. Upon addition of glutaraldehyde, higher molecular weight bands corresponding to homodimers of HSP90 (lanes 4 and 7) as well as for the HSP90-Sba1 complex (lanes 5 and 8) could be observed, whereas no interaction between HSP90 and mScarlet-I was apparent (lanes 6 and 9). Shown is one out of two representative SDS gels (see Figure S5). Both generated the same results.

As a second method for confirming and characterizing inhibitors of HSP90–Sba1 binding, we established a cross-linking SDS-PAGE analysis of *C. albicans* HSP90 and the HSP90–Sba1 complex formation (Figure 6). The applied analysis is based on an adapted protocol from Richter et al. 2001 [46]. Glutaraldehyde was used as a cross-linking agent. Glutaraldehyde reacts with different nucleophilic amino acids and can be used to detect PPIs [47]. If glutaraldehyde was added to samples containing HSP90, a protein band at a molecular weight between 150 and 200 kDa, corresponding to an HSP90 dimer (165 kDa), was observed (lanes 4 and 7). Without glutaraldehyde, only a band corresponding to the HSP90 monomer at 85 kDa was apparent (lanes 1–3). This indicates that the *C. albicans* HSP90 dimer is not SDS-resistant and requires prior covalent cross-linking by glutaraldehyde to be visible on an SDS-PAGE gel. Additionally, glutaraldehyde-containing samples with HSP90 and Sba1 showed a higher molecular weight band in the presence of ATP as well as AMP-PNP above 200 kDa (lanes 5 and 8) than that of HSP90 alone (compare lanes 4 and 5 with lanes 7 and 8). The HSP90-Sba1 complex consists of two HSP90 monomers as well as two Sba1 molecules, resulting in a combined molecular weight of 216 kDa. In contrast, the control samples containing HSP90 and mScarlet-I showed only a band corresponding to an HSP90 dimer and a lower stretched-out band between 30 and 85 kDa. Thus, there was no indication of an HSP90-mScarlet-I complex formed by glutaraldehyde cross-linking (see lanes 6 and 9). Since mScarlet-I is an SDS-resistant protein, the mScarlet-I fluorescence can be visualized after running the gel and prior to Coomassie staining under UV light. Matching the observation of the staining results, we did not observe red fluorescence corresponding to mScarlet-I in the higher molecular weight band, but only in the lower band of lanes 6 and 9 (image not shown). This illustrates the specificity of the cross-linking for HSP90–Sba1 binding detection. A virtue of this method is that no fluorophores, which can affect the PPI, are necessary.

Potentially, hits can be verified as disrupting the HSP90–co-chaperone binding with this method. Furthermore, theoretically, the effect of compounds on the homodimerization of HSP90 can also be assessed. This method would primarily give a qualitative readout about the compound effect.

### 2.9. Effect of Co-Chaperones and Geldanamycin on ATPase Activity of C. albicans HSP90

An additional method for profiling the inhibitors of HSP90–Sba1 binding is based on measuring their effect on the HSP90 ATPase activity. The conformational cycle of HSP90 is driven by the binding and hydrolysis of ATP at the NTD ATP-binding site [1]. ADP has been described to inhibit the HSP90 ATPase activity, whereas for other nucleotides such as AMP or cAMP, no corresponding data are available [48]. The influence of active compounds on the binding of co-chaperones Sba1 and Aha1 to HSP90 can be investigated, as both co-chaperones modify the ATP turnover of HSP90. The binding of Sba1 to HSP90 leads to a temporary arrest of HSP90 in a “non-hydrolyzing” conformation, consequently showing a decrease in ATP hydrolysis [8,33,34].

**Figure 7:**
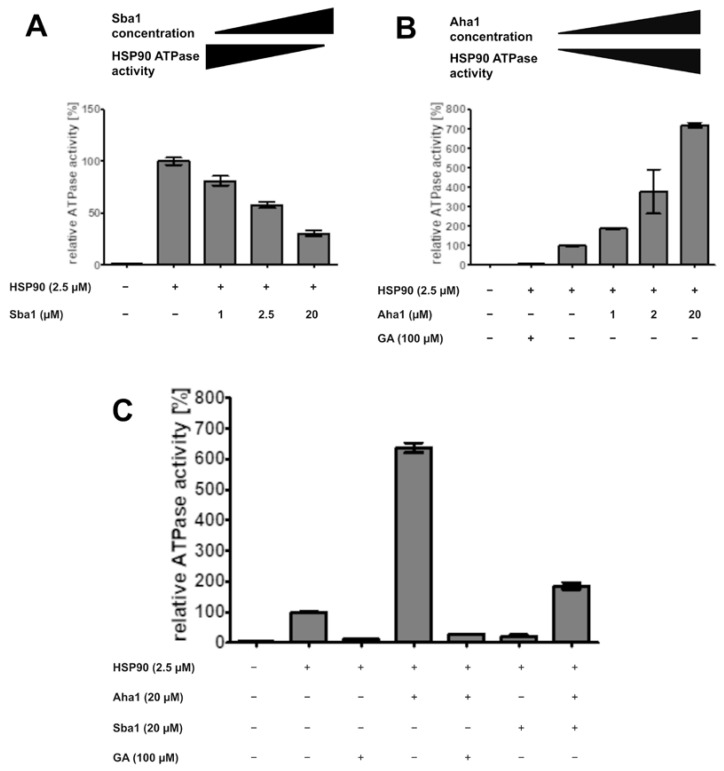
Effect of co-chaperones and geldanamycin (GA) on ATPase activity of *C. albicans* HSP90. ATPase activity of HSP90 was determined with the ADP-Glo™ assay. ATPase activity was normalized to the measurement with only HSP90. (**A**) Increasing concentrations of Sba1 were incubated with HSP90 in reaction buffer containing 100 µM ATP for 1 h at 30 °C. Sba1 stabilizes HSP90 in its ATP-bound conformation leading to a reduction in HSP90 ATPase activity to a maximum of about 30% of its original ATPase activity. (**B**) Increasing concentrations of Aha1 were incubated with HSP90 in a reaction buffer containing 1 mM ATP for 1 h at 30 °C. Aha1 activates HSP90’s ATPase activity, enhancing ATP hydrolysis by HSP90 by up to 7-fold. (**C**) ATPase reaction was performed in reaction buffer containing 1 mM ATP for 1 h at 30 °C. The HSP90-specific ATP-competitive inhibitor geldanamycin (GA) reduces ATP hydrolysis by HSP90 to blank levels. Co-incubation of Aha1 and Sba1 at equimolar concentrations shows an overall 1.9-fold increase in ATPase activity, indicating a stronger affinity of Aha1 to HSP90 than Sba1. Error bars represent the standard deviation. Measurements were performed in triplicates.

This phenomenon was confirmed for *C. albicans* HSP90–Sba1 binding. Incubation of HSP90 (2.5 µM) with increasing amounts of Sba1 (1–20 µM) showed a dose-dependent decrease in HSP90 ATPase activity (Figure 7A). Sba1 binding led to a reduction in HSP90 ATPase activity to up to 30% of its original ATP turnover. Previous studies reported a decrease in ATPase activity to up to 50% of its initial value (in the absence of Sba1) for *S. cerevisiae* HSP90/Sba1 complexes [8,33]. However, for the *human* HSP90α–p23 interaction, a complete abrogation of HSP90 ATPase activity upon HSP90’s binding to p23 was observed [34]. Because of the close relation of *C. albicans* and *S. cerevisiae*, a similar influence of Sba1 on HSP90 ATPase activity is expected. This difference in p23 and Sba1 influence on ATPase activity of *human* and *C. albicans/yeast* HSP90 is interesting considering the implications for selective drug development potential for *C. albicans* HSP90-Sba1 interaction inhibitors and should be further investigated.

Contrary to the effect of Sba1, binding of co-chaperone Aha1 (Accelerator/Activator of HSP90 ATPase 1) to HSP90 leads to a strong acceleration in ATP hydrolysis of *S. cerevisiae* HSP90 and *human* HSP90β [49]. We noticed the same effect for the *C. albicans* binding of HSP90 (2.5 µM) to Aha1 (1–20 µM). Aha1 binding enhanced the HSP90 ATP hydrolysis rate by up to 7-fold at a molar excess of 8-fold Aha1/HSP90 (Figure 7B). Wolmarans et al. reported an up to ~20-fold stimulation in ATPase activity for *S. cerevisiae* HSP90 in a range of 5–10-fold molar excess Aha1/HSP90 [32].

The ATP-competitive inhibitor geldanamycin showed an almost complete abrogation of HSP90 ATPase activity to nearly blank levels (Figure 7B,C). Geldanamycin also interferes with Aha1 binding to HSP90, as it leads to a decrease in HSP90 ATPase activity (Figure 7C).

Interestingly, the simultaneous incubation of HSP90 (2.5 µM) with equimolar amounts of Sba1 (20 µM) and Aha1 (20 µM) leads to an overall increase in HSP90 ATPase activity of almost 2-fold. This indicates that the binding affinity of *C. albicans* Aha1 to HSP90 is higher than that of Sba1. Indeed, *K*_d_ values in the presence of ATP or AMP-PNP reported for the HSP90–Aha1 complex are generally lower than for HSP90–Sba1 binding (0.16–0.2 µM vs. 0.5–1.75 µM, respectively) [8,33,34,49,50]. Another implication is that this effect is either through Aha1 and Sba1 competition for binding to HSP90, or that theoretically, an asymmetric complex consisting of an HSP90 dimer and one Aha1 molecule as well as one Sba1 molecule is formed [51]. This complex could lead to an activation, but reduced activation of HSP90 ATPase activity when compared to HSP90 alone or HSP90 and Aha1 without Sba1. As Aha1 and Sba1 have a partially common binding site, the competition for binding to a shared binding interface is the likely scenario [52,53].

With the ADP-Glo™ assay, APIs can be characterized regarding their influence on the HSP90 ATPase activity alone (potential ATP-competitiveness and general ATPase rate modification). Also, PPI inhibitors can potentially be identified as such if disruption of the influence of the co-chaperones on the HSP90 ATP hydrolysis is observed while the basal HSP90 ATP turnover remains unaffected [54]. Since Aha1 and Sba1 show partially overlapping binding sites on HSP90 (bearing in mind that there are two potential binding sites per HSP90 dimer), if compounds disrupt the binding of both Aha1 and Sba1, information about the possible binding mode of a hit compound can be obtained [52].

## 3. Discussion

### 3.1. HSP90-Sba1 and HSP90α-p23 Binding as Drug Targets for Antifungal Drug Development or Cancer Therapy

While HSP90 is heavily conserved across species, co-chaperones like Sba1 are less so (Figure S6A). In fact, *C. albicans* HSP90 shows a sequence identity of 62.4% and 78% similarity with the *human* homolog HSP90α, whereas *C. albicans* Sba1 and *human* p23 share a sequence identity of only 29% and similarity of 50%. Thus, it has been speculated that inhibition of this HSP90–co-chaperone interaction is a promising target for the development of new antifungal drugs.

To further investigate structural differences of Sba1 and p23 and their conformations when binding to HSP90, we superimposed a homology model of the *C. albicans* HSP90/Sba1 complex based on the crystal structure of a *yeast* HSP90/Sba1 complex (2CG9) with a cryo-EM structure of *human* HSP90α/p23 (7L7J) (see Figure S6C). Superimposition of Sba1 and p23 also revealed distinct structural differences.

Clinically, Sba1-deletion mutants have been shown to ameliorate azole susceptibility of formerly drug-resistant *C. albicans* strains [16]. Sba1 is involved in virulence traits such as biofilm formation. HSP90 is essential for cell survival, whereas Sba1 is not. Ergo, new drugs targeting the *C. albicans* HSP90-Sba1 binding might be best suited for combinatorial treatment with fungicidal drugs such as azoles. Also, drugs inhibiting the binding of HSP90 with this chaperone might not be subject to such high evolutionary pressure of developing resistance to the drug, as opposed to drugs targeting essential structures. This might pose an advantage in treating drug-resistant fungal infections.

Mutational studies of *human* p23 and *yeast* Sba1 indicate hotspot amino acids involved in the binding of HSP90 and Sba1 (Figure S6B). The mutation of the conserved amino acids F121A and W124A in *yeast* Sba1 (corresponding to F120 and W123 in *C. albicans*) was shown to result in a distinct reduction in binding to HSP90 [55]. Furthermore, a different study showed that mutation of I117N in *yeast* Sba1 (corresponding to I116 in *C. albicans*) led to a loss in HSP90 binding and caused severe loss of function *in vivo* [56]. While the DFxxW motif of Sba1/p23 is conserved, the I116 in *C. albicans* Sba1 corresponds to L99 in p23. Moreover, prior amino acids 112–115 (close to the lid segment of HSP90) also differ between Sba1 and p23. Of these amino acids, H114 reaches into a buried pocket of HSP90. This could potentially qualify it as a target site for selective inhibitor development.

Differences between *yeast* Sba1- and *human* p23-mediated inhibition of the HSP90 ATPase activity have been described, supporting the possibility for selective drug development [8,34].

Inhibitor development of PPIs of HSP90 with its co-chaperones has been the subject of investigation. To date, two inhibitors of the HSP90–p23 interaction, the natural products gedunin and ailanthone (AIL), have been published [57,58]. Interestingly, gedunin seems to bind somewhere close to the C-terminal tail region of p23, as discovered by mutational analysis. Additionally, the proposed binding site of AIL on p23 (inferred from docking) is mainly not conserved and partially includes amino acids (K95, K112 in *C. albicans* Sba1) suspected to be involved in gedunin binding.

These characteristics indicate the potential for selective *C. albicans* inhibitor development. Apart from Sba1 inhibition for antifungal drug development, inhibition of *human* p23 has potential for anticancer therapy [13,14]. For this purpose, targeting the amino acids F103 and W106 of p23 to develop inhibitors can also be considered.

Overall, inhibition of either Sba1 or p23 constitutes an attractive target for drug development of anti-infectives or anticancer agents. With the developed FRET assays presented in this study, it is possible to both identify new lead structures of *C. albicans* HSP90–Sba1 and *human* HSP90α–p23 inhibitors by screening compound libraries and testing compounds based on rational drug design approaches.

### 3.2. ATP-Competitive Inhibitors

So far, to our knowledge, exclusively ATP-competitive inhibitors targeting the HSP90 NTD nucleotide-binding pocket have entered clinical trials. Generally, the development of this class of HSP90 inhibitors has received the most attention. However, due to several reasons limiting efficacy and the occurrence of toxic side effects, none of these APIs reached market authorization until last year [59]. Now, pimitespib, an inhibitor of isoforms α and β of HSP90, is the first drug that has received market authorization for the treatment of gastrointestinal stromal tumors (GISTs) in Japan [60]. Due to this recent development and to presumably increase the hit rate of our library screening, we chose to screen a nucleoside-mimetics library.

Regarding the potential of developing species-selective ATP-competitive inhibitors of *C. albicans* HSP90, we compared the amino acids of the NTD nucleotide-binding site with those of the *human* isoforms HSP90α, HSP90β, Grp94 and TRAP-1 (see Figure S7A). Predominantly, these amino acids are heavily conserved. In fact, there is only one amino acid difference (L176/Val, numeration according to *C. albicans* HSP90) in comparison to *human* HSP90α/β isoforms [61]. Both are chemically similar. We also investigated the structural similarity of the nucleotide-binding pockets by superimposing cryo-EM structures of *yeast* HSP90 (PDB: 6XLC) and *human* HSP90β (PDB: 8EOB) (see Figure S7B). Since no structure of full-length *C. albicans* HSP90 is available, we chose one of the closely related *S. cerevisiae* HSP90. The amino acids of *C. albicans* and *S. cerevisiae* HSP90 involved in nucleotide binding are fully conserved. Structural alignment also showed a high three-dimensional similarity.

From this standpoint, there seems to be a limited potential for the development of species-selective HSP90 inhibitors that target the nucleotide-binding site. However, protein conformation is also recognized to be a driver in isoform selectivity [62]. This would potentially translate to species selectivity as well. Reported differences in ATPase activity between *yeast* and *human* HSP90 (1 ATP/min for *yeast* Hsp90 and 0.1 ATP/min for *human* HSP90) indicate differences in conformational flexibility influencing ATP turnover [63,64,65]. It has been reported that although the apo, ATP and ADP-bound states of *yeast* and *human* HSP90 conformations are universal, there exist differences in the conformational equilibrium of these states. Unlike the *yeast* homolog, *human* HSP90 favors the open conformation even when bound to AMP-PNP [66]. Furthermore, it has been shown that known *human* HSP90 inhibitors targeting the NTD show different efficacies in *Candida* species [67].

In fact, some efforts to develop ATP-competitive fungal-selective HSP90 inhibitors have been made [68,69,70]. These studies support the hypothesis that variations in protein flexibility can be utilized to derive *C. albicans*-specific HSP90 inhibitors. So far, an inhibitor derived by modification of the parent compound radicicol with >25-fold selectivity for *C. albicans* over *human* HSP90α and β has been reported [68]. Also, targeting other structural elements of HSP90 apart from the nucleotide-binding site could be a strategy. To date, the development of isoform-selective inhibitors of *human* HSP90α, β, Grp94 and TRAP-1 has been achieved [61,71,72,73]. In the case of developing an HSP90α-selective inhibitor, the group of Brian Blagg exploited a difference in only one amino acid (S52/A52). Still, a compound with >50-fold selectivity over HSP90β inhibition was developed [71]. Grp94- and HSP90β-specific inhibitors were developed by researchers taking advantage of unique pockets in the NTD ATP-binding site in these isoforms [61,72,74].

Furthermore, the generation of a TRAP-1-selective inhibitor was achieved not by exploiting minute structural differences of the ATP-binding site, but rather by designing a subcellular compartment-specific inhibitor through mitochondrial accumulation [73]. Recently, Wang et al. constructed new antifungal compounds by adding a benzamidine moiety (BM) leading to fungal accumulation [75]. Thus, aiming for this approach by generating HSP90 inhibitors conjugated with a benzamidine residue might be a promising strategy to improve antifungal effects while potentially reducing host toxicity. Since BM is also considered a mitochondria-targeting warhead, the possible occurrence of host toxicity due to TRAP-1 inhibition should be assessed early on.

Ultimately, it can be concluded that the development of NTD ATP-binding site species-selective inhibitors is a challenging task better suited for rational drug design. We therefore see higher potential in identifying new species-selective antifungal drugs through HTS targeting PPIs of HSP90 with co-chaperones such as Sba1. Our rationale in screening the nucleoside-mimetics library in the case of *C. albicans* HSP90 inhibitor discovery was therefore also to further validate the assay. Apart from this, the discovery and development of new ATP-competitive HSP90 inhibitors as therapeutics for cancer and neurodegenerative diseases remains a viable approach.

### 3.3. HTS in HSP90 Drug Discovery

Generally, several HSP90 inhibitor scaffolds that have been clinical candidates have emerged from HTS campaigns. These include SNX-5422 and NVP-AUY922 (developed from CCT018159 hit in HTS), underlining the applicability of HTS in drug discovery and particularly HSP90 inhibitor development [76,77,78]. Also, an HTS assay for identifying HSP90–co-chaperone inhibitors in a 96-well format has been reported [79].

We report the first HTS-*capable* assays specifically aimed at the discovery of *C. albicans* HSP90-Sba1 and *human* HSP90α-p23 inhibitors in a 384-well format. The developed assays are cheap and can be performed quickly without complicating features such as necessary washing steps.

### 3.4. Main Findings

The main findings and conclusions of the study are as follows:

1. The developed FRET assays can reliably detect inhibitors of *C. albicans* HSP90–Sba1 and *human* homologous HSP90α–p23 binding as well as ATP-competitive HSP90 inhibitors that disrupt this binding. They show high robustness and low data variability with determined *Z*’ factors of 0.58 and 0.32. Following the definition of Zhang et al., the *Z*’ factor was calculated using only the control data. The *Z*’ factor thus represents a parameter that can be utilized for quality assessment in assay development and optimization. The *Z* factor is calculated including the signal of screened compounds of a screening assay [37]. For the conducted screening in duplicates, a *Z* factor of 0.68 and 0.57 could be achieved. The *Z* factors were in our case higher than *Z*’ because they were calculated based on the average signal of the compounds screened in duplicates. Screening in duplicates thus led to a higher robustness of the assay readout. This demonstrates the high-throughput capability of the assays. Due to the simplicity of the assays, they are suitable for automated liquid handling and screening of large compound libraries. Further miniaturization of the current 384-well to a 1536-well format could be feasible to improve throughput and reduce protein and compound consumption. However, in the current format, the assays can also be considered as HTS-*capable*.
2. PAINs can be detected by the developed assays. If the donor emission is also reduced concomitantly with a decrease in FRET emission, these compounds can be identified as interfering with the readout signal and not disrupting the PPI per se. In this way, PAINs can be excluded from further investigation already at the stage of first screening in a simple way. The recognition of EGCG as a PAIN illustrates this capability.
3. For the potential discovery of new antifungal drugs, selectivity for the pathogenic target structure is of high importance for circumventing host toxicity and reducing the risk of adverse effects. With the assays, identified hit structures can be characterized for their species selectivity by determining the *IC*_50,rel_ values of both fungal and human protein interactions.
4. Upon hit finding, we propose checking for ATP-competitiveness by assessing *IC*_50,rel_ values by varying the ATP concentration in the presence of a fixed hit compound concentration with the FRET assays and/or evaluating the influence of the hit substance on HSP90 ATPase activity with the ADP Glo™ assay. Furthermore, the effects of identified APIs on HSP90-Sba1 complex formation as well as HSP90 homodimerization could be analyzed via cross-linking SDS-PAGE.
5. With the assays, accurate *K*_d_ values of both *C. albicans* HSP90-Sba1 and *human* HSP90α-p23 binding could be determined.

Proposed workflows for inhibitor identification and profiling are included in the Supplementary Materials (Figures S8 and S9).

## 4. Materials and Methods

### 4.1. Plasmid Construction and Mutagenesis

The DNA sequences for *C. albicans* HSP90 (UniProtKB: P46598), Sba1 (A0A1D8PQ94) and Aha1 (A0A1D8PU72) were generated from the amino acid sequence with EMBOSS Backtranseq and codon-optimized for expression in *E. coli*. Gene strings of these sequences were ordered from Invitrogen Thermo Fisher Scientific/Life Technologies GmbH (Darmstadt, Germany) and Twist Bioscience (South San Francisco, CA, USA). The sequence for *human* p23 (Q15185) was obtained from Addgene plasmid 108225 (Addgene HQ, Watertown, NY, USA). The DNA sequence for HSP90α (P07900) was obtained from a plasmid generated in a previous work of our lab. All sequences for proteins of interest were cloned into the expression vector pET-11d, with an N-terminal His_6_-tag followed by a sequence coding for an FXa interface, an XhoI restriction site, the sequence of the protein of interest and a KpnI restriction site. For the generation of the HSP90-mNeonGreen plasmid, the sequence of mNeonGreen from *Branchiostoma lanceolatum* [80] was integrated to the 3′ (corresponding to C-terminus in the translated protein sequence) of the HSP90 sequence, connected by a flexible linker (GGGGS). The incorporation of the mNeonGreen sequence was performed by In-Fusion Cloning (Clontech Laboratories, Takara, Saint-Germain-en-Laye, France). Analogously, the sequence of mScarlet-I [21] was fused to the 3′ of Sba1 for the construction of the Sba1-mScarlet-I expression plasmid. All other FRET plasmids were cloned via restriction–ligation cloning based on these plasmids. Codons for the E36A mutation in the sequence of *C. albicans* HSP90 and corresponding E47A substitution in *human* HSP90α sequence were introduced by site-directed mutagenesis.

### 4.2. Protein Purification

For expression of *C. albicans* His_6_-HSP90, His_6_-HSP90-mNeonGreen, His_6_-HSP90E36A-mNeonGreen, His_6_-Aha1, His_6_-Sba1 and His_6_-Sba1-mScarlet-I, *E. coli* BL21(DE3) cells containing the corresponding plasmids were cultivated in TB medium (15 g tryptone, 30 g yeast extract, 5 mL glycerol per liter) at 37 °C, 200 rpm, to an optical density of 0.6–0.8. The plasmids for the *human* homologs His_6_-HSP90α-mNeonGreen, His_6_-HSP90αE47A-mNeonGreen, His_6_-p23 and His_6_-p23-mScarlet-I were transformed into *E. coli* Rosetta2(DE3) pLysS (71403 Novagen, Merck Darmstadt, Germany) cells and cultivated under the same conditions. Protein expression was induced with 0.5 mM IPTG, and cells were then incubated for another 24–72 h at 18 °C, 180 rpm. For the expression of His_6_-mNeonGreen and His_6_-mScarlet-I, corresponding plasmids were transformed into *E. coli* BL21(DE3) cells and cultivated at 37 °C in LB medium to an optical density of 0.5. Protein expression was induced by the addition of 1 mM IPTG. Cells were further cultivated for 4 h at 30 °C, 200 rpm. Cells were harvested by centrifugation. The cell pellet was resuspended in HEPES buffer (40 mM HEPES/KOH pH 7.5, 20 mM KCl). Lysozyme (0.2 g/L), DNAseI (0.05 g/L), benzamidine (1 mM) and PMSF (1 mM) were added to the cell suspension, and the cells were incubated for 20 min on ice. Cells were lysed by ultrasonification and centrifuged at 100 000× *g* for 30 min at 4 °C. The supernatant was applied to Ni-NTA columns that had previously been equilibrated with HEPES buffer containing 10 mM imidazole. The column was washed thoroughly with HEPES buffer containing 20 mM imidazole to remove unspecific protein binding. Elution of the desired protein was performed with HEPES buffer containing 500 mM imidazole. As a second purification step, this elution fraction was further purified by gel filtration with a HiLoad 26/600 Superdex 200 pg column and an Äkta Start (Cytiva Marlborough, MA, USA) using the HEPES buffer as a mobile phase. After gel filtration, elution fractions containing the desired protein size and the respective fluorescent properties in the case of fluorescent fusion proteins were united and concentrated using an Amicon^®^ Ultra-15 filter unit (MWCO: 10 kDa). The quality of protein purification was assessed via SDS-PAGE analysis. Protein concentration was determined with a nanophotometer (Pearl, Implen GmbH; Munich, Germany) at 280 nm. For long-term storage, the protein solution was aliquoted, flash-frozen in liquid nitrogen and stored at −80 °C. mNeonGreen and mScarlet-I were purified solely via His_6_-tag purification.

### 4.3. FRET Measurements

FRET experiments were carried out in 384-well microtiter plates (Art. No. 3766 Corning, NY, USA, or Art. No. 781906, Greiner Bio-One GmbH, Frickenhausen, Germany) containing a final volume of 20 µL and measured with a microplate reader Infinite M200Pro (Tecan, Männedorf, Switzerland). The fluorescence was measured in three channels: excitation (ex.): 488 nm/emission (em.): 525 nm (mNeonGreen fluorescence), ex.: 561 nm/em.: 610 nm (mScarlet-I fluorescence) and the FRET channel ex.: 488 nm/em.: 610 nm. The excitation and emission bandwidths were set to 9 and 20 nm, respectively. Experiments for the determination of the equilibrium dissociation constants were performed with a filter-based microplate reader Infinite F200Pro (Tecan, Männedorf, Switzerland). The fluorescence was measured in three channels using the following filters: ex.: 485 ± 20 nm/em.: 535 ± 20 nm (mNeonGreen channel), ex.: 535 ± 20 nm/em.: 612 ± 10 nm (mScarlet-I channel) and the FRET channel ex.: 485 ± 20 nm/em.: 612 ± 10 nm. FRET emission (*Em*_FRET_) was calculated according to Song et al. 2011 [81]:

(1) $${Em}_{FRET}=\left({FL}_{DA}\right)-\left(x\times {FL}_{DD}\right)-(y\times {FL}_{AA})$$

where *FL*_DA_ is the fluorescence signal of the FRET channel and *FL*_DD_ and *FL*_AA_ are fluorescence signals from the mNeonGreen and mScarlet-I channels, respectively. The correction factors *x* and *y* were calculated as follows:

(2) $$x=\frac{{FL}_{DA}}{{FL}_{DD}}$$

for samples containing only the donor fluorophore or fluorescent fusion proteins with mNeonGreen and

(3) $$y=\frac{{FL}_{DA}}{{FL}_{AA}}$$

for samples containing only the acceptor fluorophore or fluorescent fusion proteins with mScarlet-I in the same concentration as samples containing both the donor and acceptor.

All experiments were carried out in reaction buffer (40 mM HEPES/KOH pH 7.5, 20 mM KCl, 5 mM MgCl_2_, 20 mM Na_2_MoO_4_, Triton-X 100 0.1%). HEPES and KCl were purchased from AppliChem, Darmstadt, Germany. KOH and MgCl_2_ were obtained from VWR Chemicals, Darmstadt, Germany. Na_2_MoO_4_ was from Merck, Darmstadt, Germany. Triton-X 100 was purchased from Serva, Heidelberg, Germany.

#### 4.3.1. FRET Binding Assays

For FRET binding specificity experiments, 1 µM of a donor (*C. albicans* HSP90-mNeonGreen, *human* HSP90α-mNeonGreen or mNeonGreen) and variable concentrations (0–2 250 nM, lowest non-zero concentration 53 nM) of an acceptor (Sba1-mScarlet-I, p23-mScarlet-I or mScarlet-I) were mixed in reaction buffer containing 5 mM ATP. For the determination of the equilibrium dissociation constant, for *C. albicans* HSP90E36A–Sba1 and *human* HSP90αE47A–p23, 200 nM of a donor (HSP90E36A-mNeonGreen or HSP90αE47A-mNeonGreen or mNeonGreen serving as control) was mixed with varying concentrations of an acceptor (Sba1-mScarlet-I, p23-mScarlet-I) in a range of 0–3000 nM (lowest non-zero concentration 10 nM) in reaction buffer containing 5 mM ATP. The ATP-hydrolysis-defective HSP90 mutants were used to ensure that an equilibrium of HSP90–co-chaperone binding independent of ATP concentrations can be obtained. The *Em*_FRET_ of three technical triplicates was determined. The *Em*_FRET_ values of the control mNeonGreen/Sba1-mScarlet-I or mNeonGreen/p23-mScarlet-I were subtracted from the *Em*_FRET_ of HSP90E36A-mNeonGreen/Sba1-mScarlet-I or HSP90αE47A-mNeonGreen/p23-mScarlet-I, respectively. The resulting *Em*_FRET_ was normalized to the unbound fraction (*R*). The unbound fraction is calculated based on the maximum FRET emission, assuming that all of the co-chaperone is bound to HSP90 at these acceptor concentrations. The *K*_d_ values were calculated by nonlinear regression with GraphPad Prism 5 (GraphPad Software, La Jolla, CA, USA) using the following formula:

(4) $$R=-\frac{K_{d}+T_{0}-L_{0}}{{2L}_{0}}+\sqrt{\left(\frac{K_{d}+T_{0}-L_{0}}{{2L}_{0}}\right)+\frac{K_{d}}{L_{0}}}$$

where *L*_0_ is the limiting component concentration (*C. albicans* HSP90E36A-mNeonGreen or *human* HSP90αE47A-mNeonGreen) and *T*_0_ is the varied component concentration (Sba1-mScarlet-I/p23-mScarlet-I). For the calculation of *K*_d_, the concentrations of both *L*_0_ and *T*_0_ were corrected for the purity of the protein samples as assessed by SDS-PAGE and ImageJ analysis (see Figure S10). To derive accuracy confidence intervals (ACIs), errors for concentrations of *L*_0_ and *T*_0_ were estimated by measuring the protein concentrations with a nanophotometer (Pearl, Implen GmbH; Munich, Germany) at 280 nm ten times. From these ten concentrations, the relative standard error was calculated and used as input for ACI generation. Detailed descriptions of ACI calculation are described elsewhere [29].

#### 4.3.2. Characterization of Model *C. albicans* HSP90–Sba1 and *Human* HSP90α–p23 Binding Inhibitors via FRET

For *C. albicans* Sba1 competition experiments for HSP90-mNeonGreen–Sba1-mScarlet-I binding, 1 µM HSP90-mNeonGreen and 1 µM Sba1-mScarlet-I were incubated with varying concentrations of Sba1 in a range of 0–25,000 nM (lowest non-zero concentration 12 nM) in reaction buffer containing 5 mM ATP. The experiment was performed twice in nonaplicates. For *human* p23 competition experiments for HSP90α-mNeonGreen–p23-mScarlet-I binding, 1 µM HSP90α-mNeonGreen and 1 µM p23-mScarlet-I were incubated with varying concentrations of p23 in a range of 0–50 000 nM (lowest non-zero concentration 23 nM) in reaction buffer containing 5 mM ATP. The experiment was performed twice in hexaplicates. Displayed is one of two hexaplicate measurements. Both resulted in the same *IC*_50,rel_. For the determination of the geldanamycin *IC*_50,rel_, 1 µM *C. albicans* HSP90E36A-mNeonGreen and 2 µM Sba1-mScarlet-I were mixed with geldanamycin in varying concentrations from 0–400 µM (lowest non-zero concentration 1.4 µM) in reaction buffer containing 5 mM ATP, 1% DMSO. The experiment was performed thrice in triplicates. The experiments for the determination of the geldanamycin *IC*_50,rel_ for *human* HSP90α–p23 binding were carried out analogously. Prior to the measurement, samples were incubated for 15 min at 37 °C. The experiments were performed twice in triplicates. The *IC*_50,rel_ values were calculated using the equation log (inhibitor) vs. response-variable slope (four parameters) in GraphPad Prism 5. *K*_i_ values for inhibition of HSP90-mNeonGreen–Sba1-mScarlet-I/p23-mScarlet-I binding by non-labeled Sba1/p23 were calculated with One Site—Fit *K*_i_ in GraphPad Prism 5. Geldanamycin was purchased from Carl Roth (Karlsruhe, Germany). For ATP *EC*_50,rel_ determination experiments, 1 µM HSP90E36A-mNeonGreen and 2 µM Sba1-mScarlet-I were mixed with ATP in a concentration range of 0–12,500 µM (lowest non-zero concentration 3 µM). The experiment was performed twice in triplicates. HSP90αE47A-mNeonGreen (1 µM) was incubated with p23-mScarlet-I (2 µM) in reaction buffer. ATP was titrated in a concentration range of 0–6250 µM (lowest non-zero concentration 3 µM). The experiment was performed twice in triplicates. The *EC*_50,rel_ values were calculated using the log (agonist) vs. response-variable slope (four parameters) equation in GraphPad Prism 5.

#### 4.3.3. FRET Inhibitor Screening Assays

For screening for the identification of ATP-competitive *C. albicans* HSP90 and HSP90-Sba1 binding inhibitors, 10 µL of HSP90-mNeonGreen (2 µM) and Sba1-mScarlet-I (4 µM) in HEPES buffer (40 mM HEPES/KOH pH 7.5, 20 mM KCl) was mixed with 10 µL of 200 µM compound solution in 2x reaction buffer (40 mM HEPES/KOH pH 7.5, 20 mM KCl, 10 mM MgCl_2_, 40 mM Na_2_MoO_4_, Triton-X 100 0.2%), 6 mM ATP, 2% DMSO, resulting ultimately in constant HSP90-mNeonGreen concentrations of 1 µM and Sba1-mScarlet-I 2 µM in reaction buffer. Samples for binding control (3 mM ATP, final concentration) and non-binding control (no ATP) were prepared in the same manner without compounds. The samples were incubated for 15 min at 30 °C prior to measurement. The assay for the *human* HSP90α–p23 binding inhibitor identification was performed analogously, with the exception that samples were incubated for 15 min at 37 °C prior to measurement. Since binding samples show both a high Em_FRET_ as well as a reduction in donor emission (*FL*_DD_), we calculated the quotient (*Em*_FRET_/*FL*_DD_) to derive an improved signal separation and robustness. The nucleoside-mimetics library was obtained from Enamine (catalog No. NML-320-X-20, Enamine Ltd., Kyiv, Ukraine). *Z*′ and *Z* factors were calculated based on Zhang et al. [39].

### 4.4. ADP-Glo™ Assay

The ADP-Glo™ assay (Promega GmbH, Waldorf, Germany) was performed according to the manufacturer’s protocol. Briefly, the ATPase reaction was performed in 5 µL volume containing 2.5 µM HSP90 and varying concentrations of Aha1 (1 µM, 2 µM, 20 µM) or Sba1 (1 µM, 2.5 µM, 20 µM) in reaction buffer (40 mM HEPES/KOH pH 7.5, 20 mM KCl, 5 mM MgCl_2_, 20 mM Na_2_MoO_4_, Triton-X 100 0.1%) and either 100 µM ATP or 1 mM ATP, respectively. The reaction mixture was incubated for 1 h at 30 °C. After 5 min re-tempering to room temperature, 5 µL of ADP-Glo reagent was added to the samples to terminate the kinase reaction and deplete the remaining ATP for 40 min at room temperature. Afterward, 10 µL Kinase Detection Reagent was added to convert the generated ADP back to ATP. In that way, synthesized ATP was measured in a luciferase/luciferin reaction. Luminescence was detected with an Infinite M200 Pro (Tecan, Männedorf, Switzerland) in a 384-well microtiter plate (Art. No. 781075, Greiner Bio-One GmbH, Frickenhausen, Germany) after 40 min for 100 µM ATP samples, or 60 min for 1 mM ATP samples. The luminescence intensity of the sample containing only HSP90 was set to 100%, and the luminescence intensities of the other samples were related to it.

### 4.5. Cross-Linking SDS-PAGE Analysis of C. albicans HSP90 dimers and HSP90Sba1 Complex with Glutaraldehyde

Cross-linking was performed according to an adapted protocol from Richter et al. 2001 [46]. For this purpose, 10 µL of 10 µM of HSP90 and an equimolar amount of Sba1 or mScarlet-I serving as a negative control were incubated in reaction buffer (40 mM HEPES/KOH pH 7.5, 20 mM KCl, 5 mM MgCl_2_, 20 mM Na_2_MoO_4_, Triton X-100 0.1%) for 15 min at 30 °C in the presence of 5 mM ATP or 2 mM AMP-PNP as indicated. Cross-linking was induced by adding 0.5 µL of a 1:10 dilution of 25% glutaraldehyde (Merck Darmstadt, Germany) to reaction buffer and incubating it for 2 min at room temperature. The reaction was stopped by adding 2.5 µL of 1 M Tris-HCl, pH 8. Prior to the SDS-PAGE, 4 µL of 4x SDS-loading dye was added. The proteins were separated on a precast 4–15% SDS-Polyacrylamide gel (Bio-Rad Laboratories Inc., Hercules, USA). The protein ladder used was PageRuler™ Unstained (ThermoScientific, Life Technologies GmbH Darmstadt). After the completion of gel electrophoresis, photos of the gel were taken under UV-light exposure to visualize mScarlet-I fluorescence, and the gel was subsequently stained with Coomassie Brilliant Blue.

### 4.6. K_d_ Determination of AMP-PNP Binding to C. albicans HSP90 via Microscale Thermophoresis

HSP90-mNeonGreen (*C. albicans*) or mNeonGreen serving as the negative control was adjusted to 100 nM with Sba1 buffer (40 mM HEPES/KOH pH 7.5, 20 mM KCl). The ligand AMP-PNP was dissolved in 2x reaction buffer (40 mM HEPES/KOH pH 7.5, 20 mM KCl, 10 mM MgCl_2_, 40 mM Na_2_MoO_4_, 0.2% Triton-X 100), and a series of 16 1:1 dilutions was prepared using the same buffer, producing ligand concentrations ranging from 305 to 10 000 nM. For the measurement, each ligand dilution was mixed with one volume of HSP90-mNeonGreen or mNeonGreen, which led to a final concentration of 50 nM and final ligand concentrations ranging from 153 nM to 5 mM. After 5 min incubation at room temperature, the samples were loaded into Monolith NT.115 Capillaries (NanoTemper Technologies, Munich, Germany). MST was measured using a Monolith NT.115 at an ambient temperature of 25 °C. Instrument parameters were adjusted to 35% LED power and a high MST power of 80%. Data of three independently pipetted measurements were analyzed (MO.Affinity Analysis software version 2.1.3, NanoTemper Technologies, Munich, Germany) using the signal from an MST-on time of 30 s.

## 5. Conclusions

In summary, the assays presented in this study are potentially applicable for both antifungal drug development and the drug discovery of potential new scaffolds for anticancer therapy. To identify new lead structures in both areas, large compound libraries should be screened or rational approaches in drug design should be pursued. The compound libraries can include targeted PPI libraries without limitation as well as larger nucleoside-mimetics libraries. Certainly, non-targeted diversity-based libraries could be screened as well. Discovery and development of new antifungal compounds that target HSP90 and its binding to co-chaperones could provide additional therapeutical options for the treatment of drug-resistant fungal infections in clinical practice. Furthermore, the combination of these new drugs with already existing, well-established drugs could have synergistic effects and reduce the potential for the emergence of drug resistance. Due to the importance of HSP90 in many pathways regulating cell growth, division and survival, the development of new anticancer drugs targeting HSP90 would potentially provide new treatment options for a variety of cancerous manifestations. Additionally, combinatorial treatment of HSP90-targeting drugs with other established drugs could potentially enable a dosage reduction and thus result in a reduction in toxic adverse effects common in anticancer treatment.

## Data Availability

All relevant data are included in the publication and Supplementary Materials.

## Supplementary Materials

The following supporting information can be downloaded at https://www.mdpi.com/article/10.3390/ph17040516/s1, Figure S1: Equilibrium check of *C. albicans* HSP90–Sba1 and *human* HSP90α–p23 binding; Figure S2: AMP-PNP binding affinity to *C. albicans* HSP90; Figure S3: EGCG is a PAIN; Figure S4: Screening of nucleoside-mimetics library. Individual samples; Figure S5: Confirmatory cross-linking SDS-PAGE of *C. albicans* HSP90 dimers and HSP90–Sba1 binding; Figure S6: Potential for selective *C. albicans* Sba1 inhibitor development; Figure S7: The nucleotide-binding site of *C. albicans* and *human* isoforms shows high degree of conservation and high structural similarity; Figure S8: Workflow: Identification and profiling of inhibitors of *C. albicans* HSP90-Sba1 binding; Figure S9: Workflow: Identification and profiling of inhibitors of *human* HSP90α-p23 binding; Figure S10: SDS-PAGE and purity assessment of fusion proteins for *K*_d_ experiments; Table S1: Characteristics of screened HSP90 inhibitors and their influence on HSP90–Sba1/p23 binding according to the literature; Table S2: Compound list of nucleoside-mimetic library; pdf file: ACI-Kd report_C. albicans HSP90-Sba1 binding; pdf file: ACI-Kd report HSP90a-p23 binding. References [82,83,84,85,86,87,88] are cited in the Supplementary Materials.
