# Supplementary material for: FRET Assays for the Identification of C. albicans HSP90-Sba1 and Human HSP90α-p23 Binding Inhibitors
Source: Pharmaceuticals (Basel). 2024 Apr 17;17(4):516. doi: 10.3390/ph17040516 (PMC11053944; doi:10.3390/ph17040516)
Supplement: Supplementary file 1 [file pharmaceuticals-17-00516-s001.zip › Supplementary Materials_Figures and Tables.pdf]

pharmaceuticals

MDPI

Supplementary Materials

Figures

A C. *albicans*

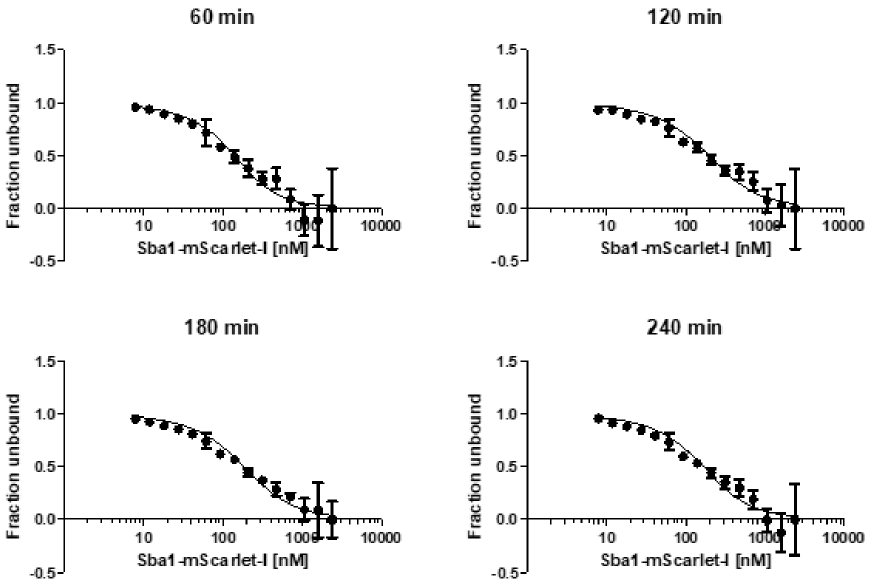

Time point (min)	K_d (nM)	PCI 95.5% (nM)	ACI 95.5% (nM)
60	50	30-80	50-70
120	100	80-130	100-140
180	100	70-120	100-130
240	80	50-110	80-110

B *human* – HSP90α

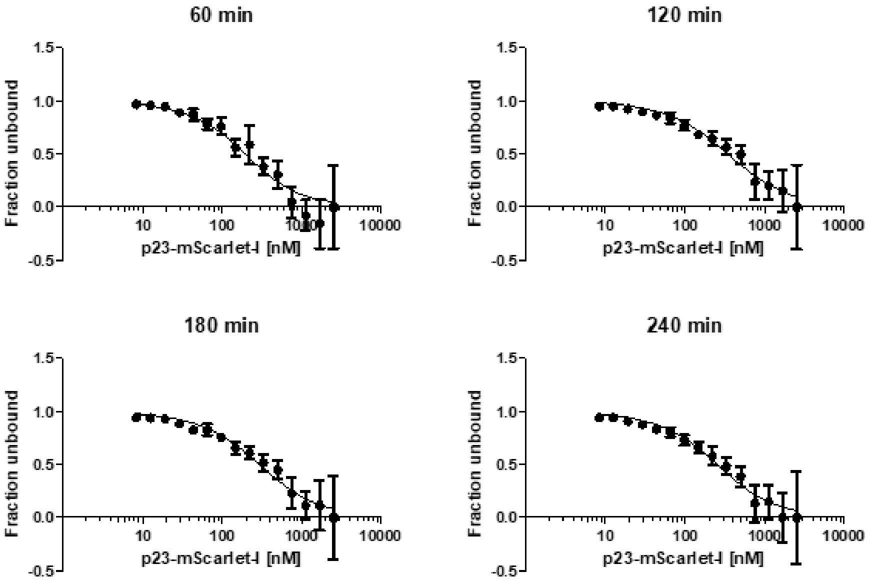

Time point (min)	K_d (nM)	PCI 95.5% (nM)	ACI 95.5% (nM)
60	120	70-170	90-140
120	270	220-320	240-320
180	220	180-260	200-270
240	180	140-210	150-220

Figure S1. Equilibrium check of *C. albicans* HSP90 – Sba1 and *human* HSP90α – p23 binding. 200 nM donor molecule was incubated at 30 °C (*C. albicans* HSP90E36A-mNeonGreen) or 37 °C (*human* HSP90αE47A-mNeonGreen) with 0-3 000 nM (lowest non-zero concentration 10 nM) of acceptor molecule (*C. albicans* Sba1-mScarlet-I) or (*human* p23-mScarlet-I) and measured every 60 min for up to 4 h. The equilibrium dissociation constant (K_d) of the interaction was calculated for all time points with GraphPad Prism5. Precision and accuracy confidence interval (PCI and ACI, respectively) at a 95.5% confidence level were determined according to the method of Wang et al. [29].

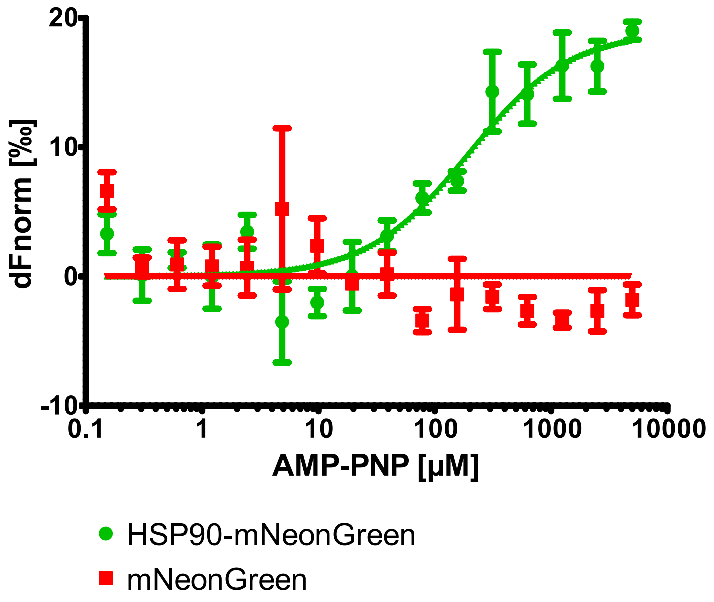

Figure S2. AMP-PNP binding affinity to *C. albicans* HSP90. In this MST experiment the concentration of HSP90-mNeonGreen (green circles) as well as for the negative control mNeonGreen (red squares) was kept constant (50 nM), while the concentration of the non-labeled ligand (AMP-PNP) was varied between 153-5 000 nM. After 5 min incubation at room temperature, the samples were loaded into Monolith NT.115 Capillaries (NanoTemper Technologies, Munich, Germany) and the MST measurement was performed at room temperature using the Monolith NT.115 (NanoTemper Technologies, Munich, Germany) at 35% LED power and high (80%) MST power. An MST-on time of 30 s was used for analysis, and a K_d of 200 ± 70 µM was derived. n = 3 independent measurements, error bars represent the standard deviation.

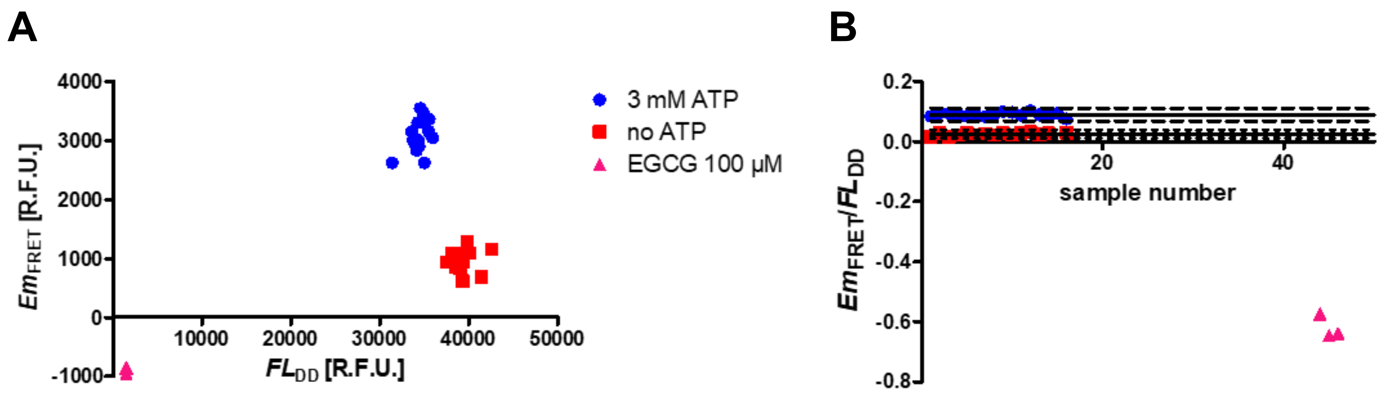

Figure S3. EGCG is a PAIN compound. **(A)** Incubation of *human* HSP90α-mNeonGreen (1 µM) and p23-mScarlet-I (2 µM) with 100 µM EGCG (pink triangles) in reaction buffer containing 3 mM ATP leads to strong decrease in FRET emission (Em_{FRET}) as well as donor emission (FL_{DD}) compared to binding control (blue circles) and non-binding control (red squares). Both binding and non-binding control contained donor and acceptor in the same concentrations with 3 mM ATP or no ATP included in the buffer. **(B)** This leads to a negative Em_{FRET}/FL_{DD} quotient. If a compound prevents binding of HSP90α and p23, both decrease in FRET emission and increase in donor emission occurs. The here observed effect of EGCG is attributed to unspecific effects such as covalent protein modifications. The same effect was observed on the binding of *C. albicans* HSP90 – Sba1 (data not shown). [R.F.U.] relative fluorescence units.

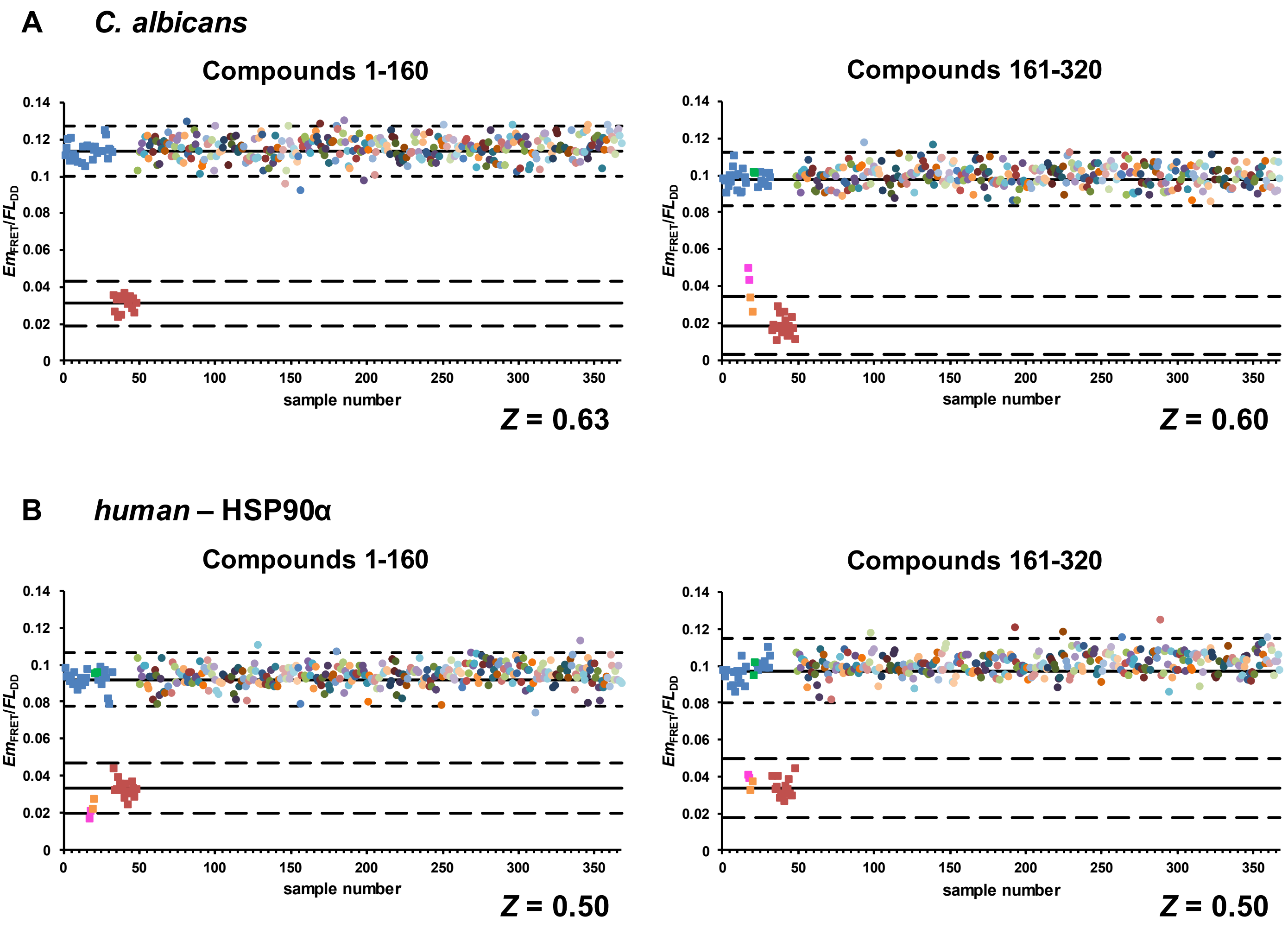

Figure S4. Screening of nucleoside-mimetics library. Individual samples. **(A)** *C. albicans* HSP90-mNeonGreen (1 µM) and Sba1-mScarlet-I (2 µM) were incubated with compounds (100 µM, colored circles) in reaction buffer 1% DMSO, 3 mM ATP for 15 min at 30 °C prior to measurement. 3 mM ATP containing samples without compound (blue squares) and no ATP samples (red squares) served as binding and non-binding controls, respectively. The inhibition controls geldanamycin (pink squares) and luminespib (orange squares) were screened at concentrations of 100 µM and 10 µM, respectively. The non-inhibition control withaferin A (green squares) was screened at 10 µM. **(B)** Screening for inhibition of *human* HSP90α-mNeonGreen – p23-mScarlet-I binding was performed analogous, except for incubating at 37 °C prior to measurement. Each color represents the signal of a compound that was screened in duplicate. The shown Z-values were calculated based on individual well signal.

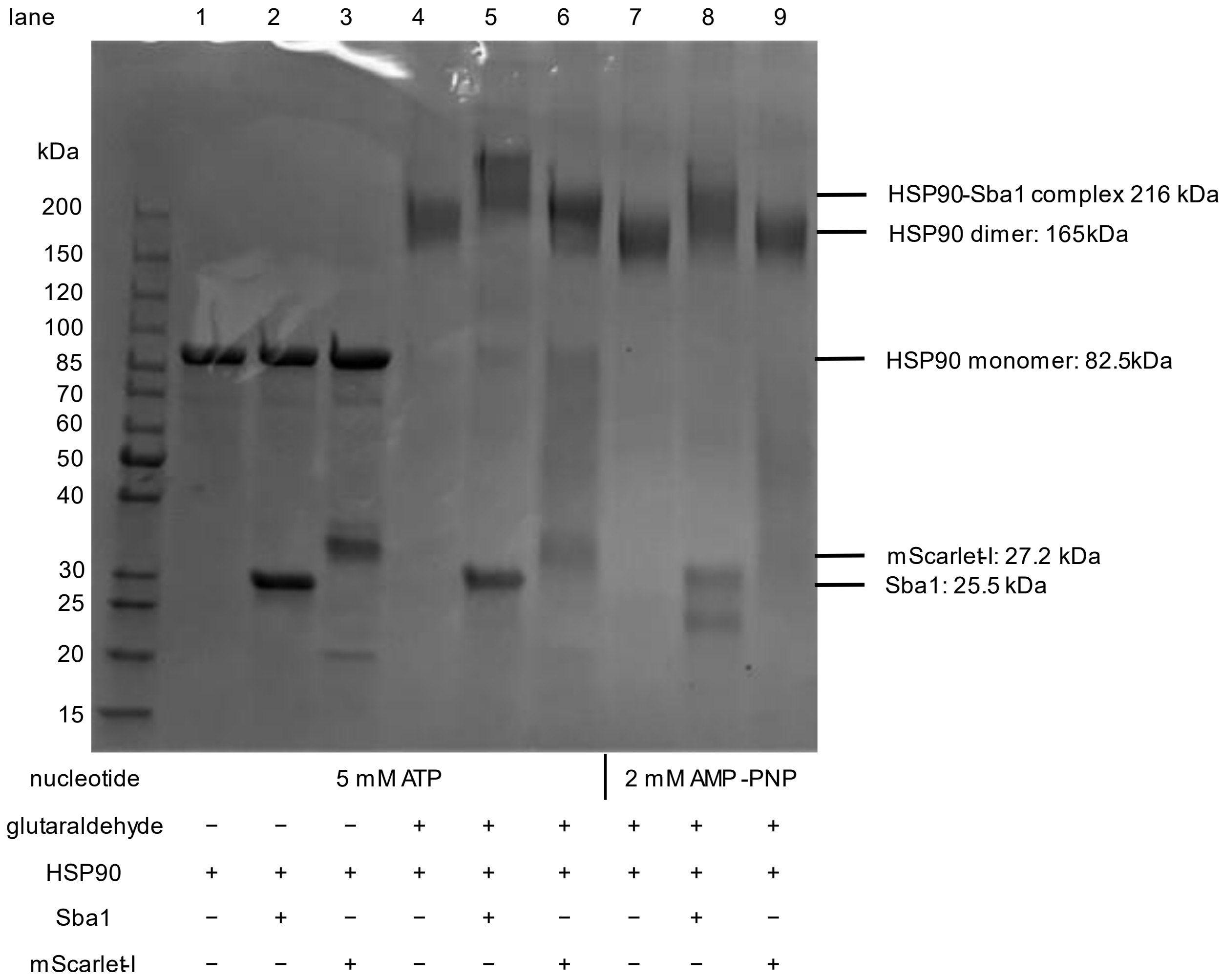

Figure S5. Confirmatory cross-linking SDS-PAGE of *C. albicans* HSP90 dimers and HSP90—Sba1 binding. The experiment was conducted analogously to Figure 6. Proteins were separated by SDS-PAGE without prior heating of samples followed by Coomassie staining. Lanes 1-3 not treated with glutaraldehyde show monomeric HSP90 (82.5 kDa) as well as Sba1 (25.5 kDa, lane 2) and mScarlet-I (27.2 kDa, lane 3). mScarlet-I was used as a negative control to show that cross-linking was specific for interacting proteins. Upon addition of glutaraldehyde higher molecular weight bands corresponding to homodimers of HSP90 (lane 4 and 7) as well as for HSP90-Sba1 complex (lane 5 and 8) could be observed, whereas no interaction between HSP90 and mScarlet-I was apparent (lane 6 and 9).

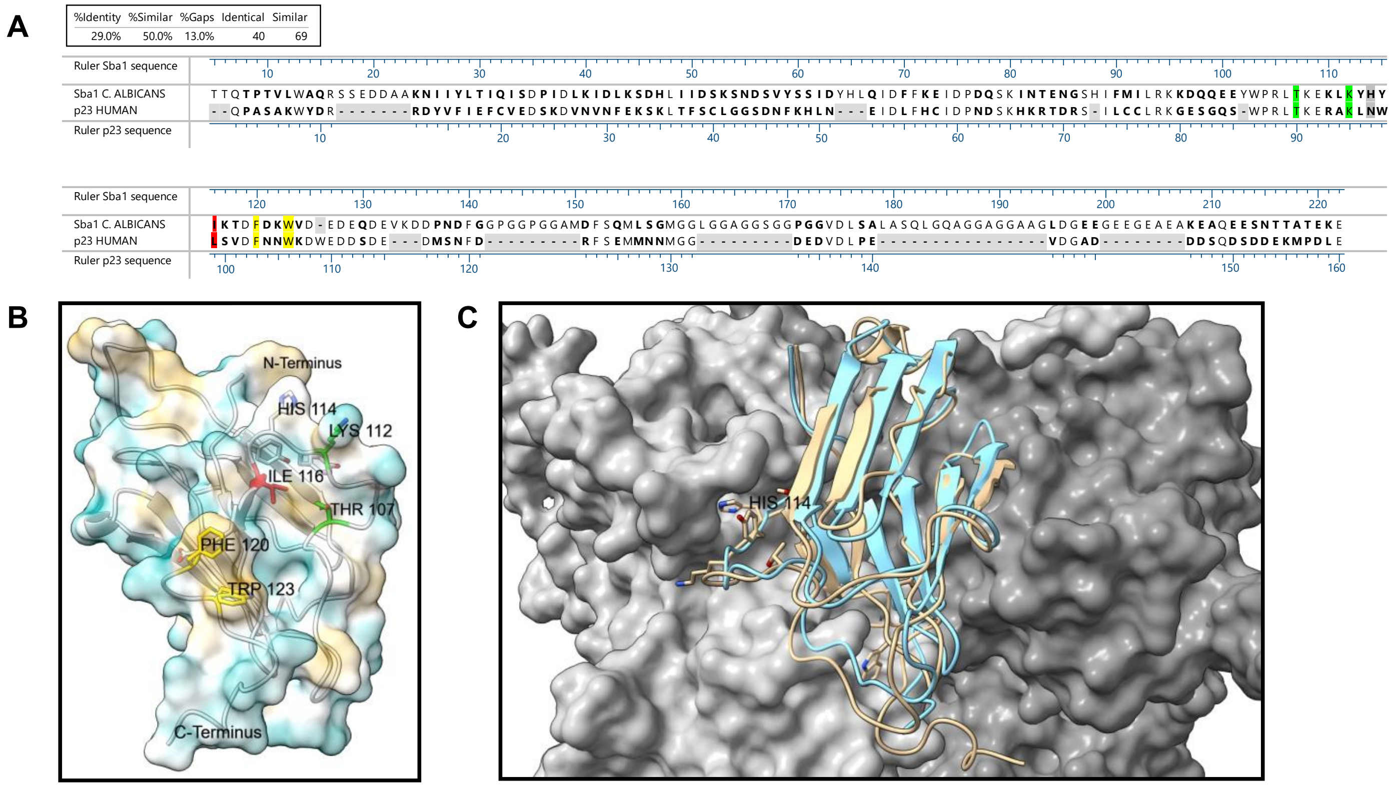

Figure S6. Potential for selective *C. albicans* Sba1 inhibitor development. **(A)** Sequence alignment of *C. albicans* Sba1 (UniProtKB: A0A1D8PQ94) with *human* p23 (Q15185). Amino acids that differ between compared sequences in bold. Sequences show low sequence identity and similarity between species. Residues important for p23/Sba1 binding to HSP90 marked yellow [55] and red [56]. Amino acids that are putatively involved in binding with the Sba1/p23 inhibitor gedunin highlighted in green [57]. **(B)** Homology model of *C. albicans* Sba1 based on crystal structure 2CG9. Hot spot amino acids involved in HSP90 or gedunin binding are colored in accordance with **(A)**. (**C)** Superimposition of homology model of *C. albicans* HSP90:Sba1 (based on 2CG9) with cryo-EM structure of *human* HSP90:p23 complex (7L7J). Whereas HSP90 sequence and structure are heavily conserved across species, Sba1 (beige) and p23 (light blue) show marked structural differences. HSP90 monomers shown in gray and dark gray. *C. albicans* His114 reaches into buried pocket of HSP90 and is not conserved, as are the amino acids in its vicinity (112-116). This could potentially be a target site for selective inhibitor development.

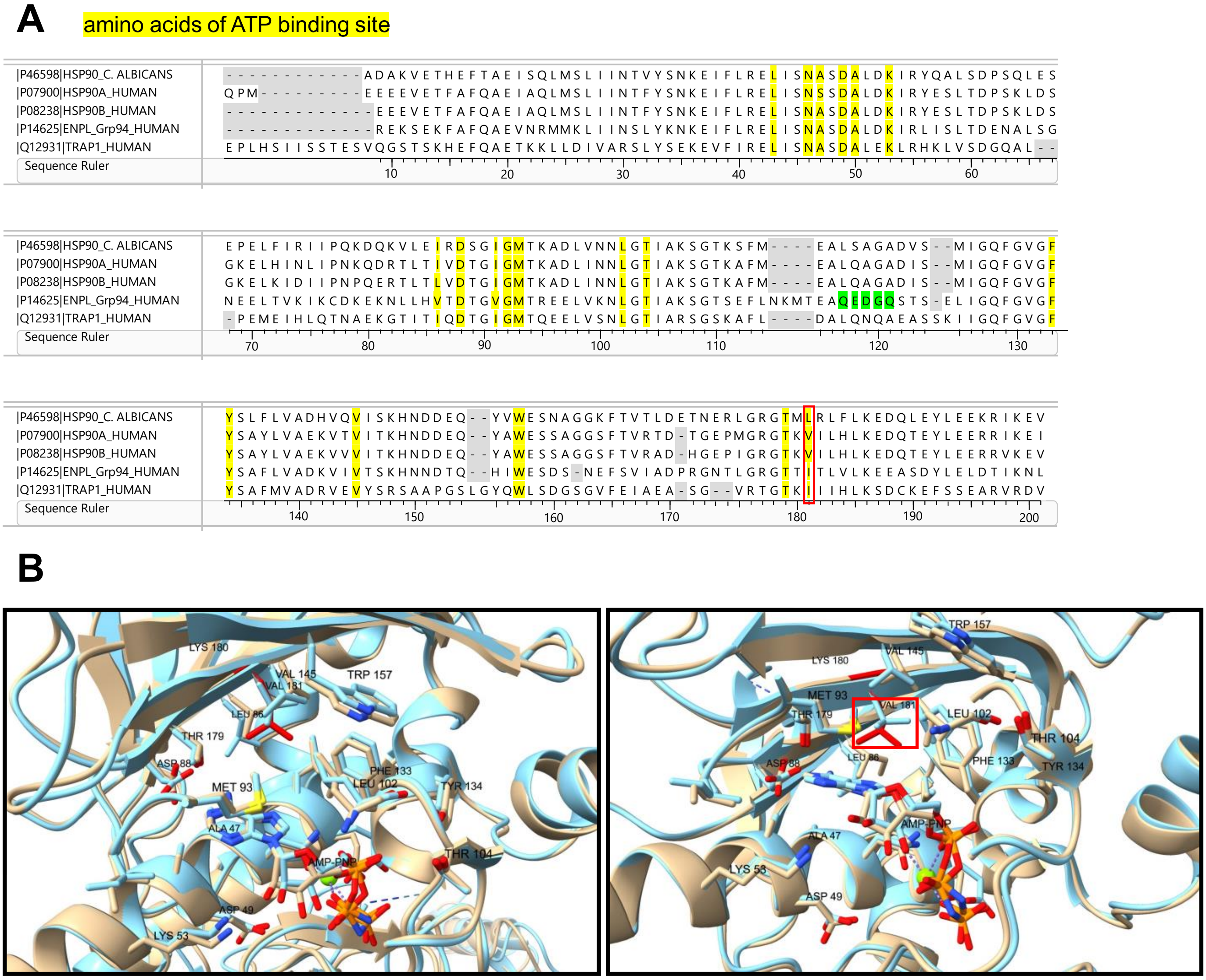

Figure S7. The nucleotide-binding site of *C. albicans* and *human* isoforms shows high degree of conservation and high structural similarity. **(A)** Sequence alignment of *C. albicans* HSP90 and the *human* isoforms of HSP90α, β, Grp94 and TRAP-1. Amino acids of the ATP binding site are highlighted in yellow according to Mishra *et al.* 2021 [71]. Only one amino acid of the binding site differs in *C. albicans* HSP90 when compared to the *human* isoforms (L176, corresponding to V181 for *human* HSP90β). This amino acid is highlighted with a red box. Five amino acid QEDGQ insertion into primary sequence of Grp94 highlighted in green. This insertion leads to a conformational change within the ATP-binding pocket of Grp94 that was exploited to develop an isoform-selective Grp94 inhibitor [72]. **(B)** Superimposition of cryo-EM structures of *S. cerevisiae* HSP90 (beige, PDB: 6XLC) and *human* HSP90β (light blue, PDB: 8EOB). Both structures were solved with HSP90 in the N-terminally dimerized conformation when bound to the non-hydrolysable ATP analogue AMP-PNP. Generally, also a high three-dimensional similarity in the conformation of involved amino acids is observed. Images show different angles of the ATP-binding site. The amino acid L176 is highlighted in red and marked with a red box in the second image. Analysis was performed with ChimeraX. **(A), (B)** Shown numeration of amino acids based on the *human* HSP90β sequence.

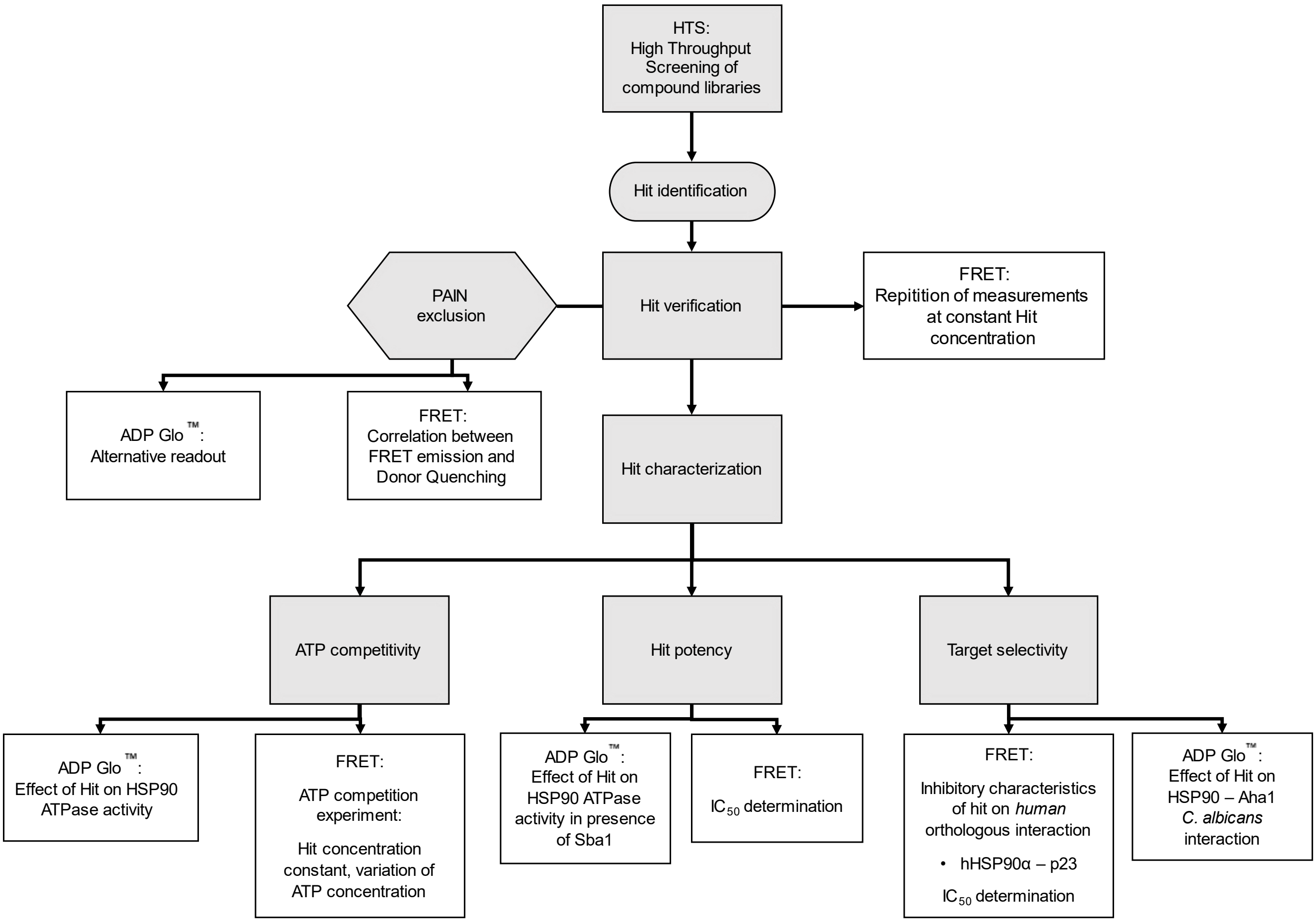

Figure S8. Workflow: Identification and profiling of inhibitors of *C. albicans* HSP90-Sba1 binding.

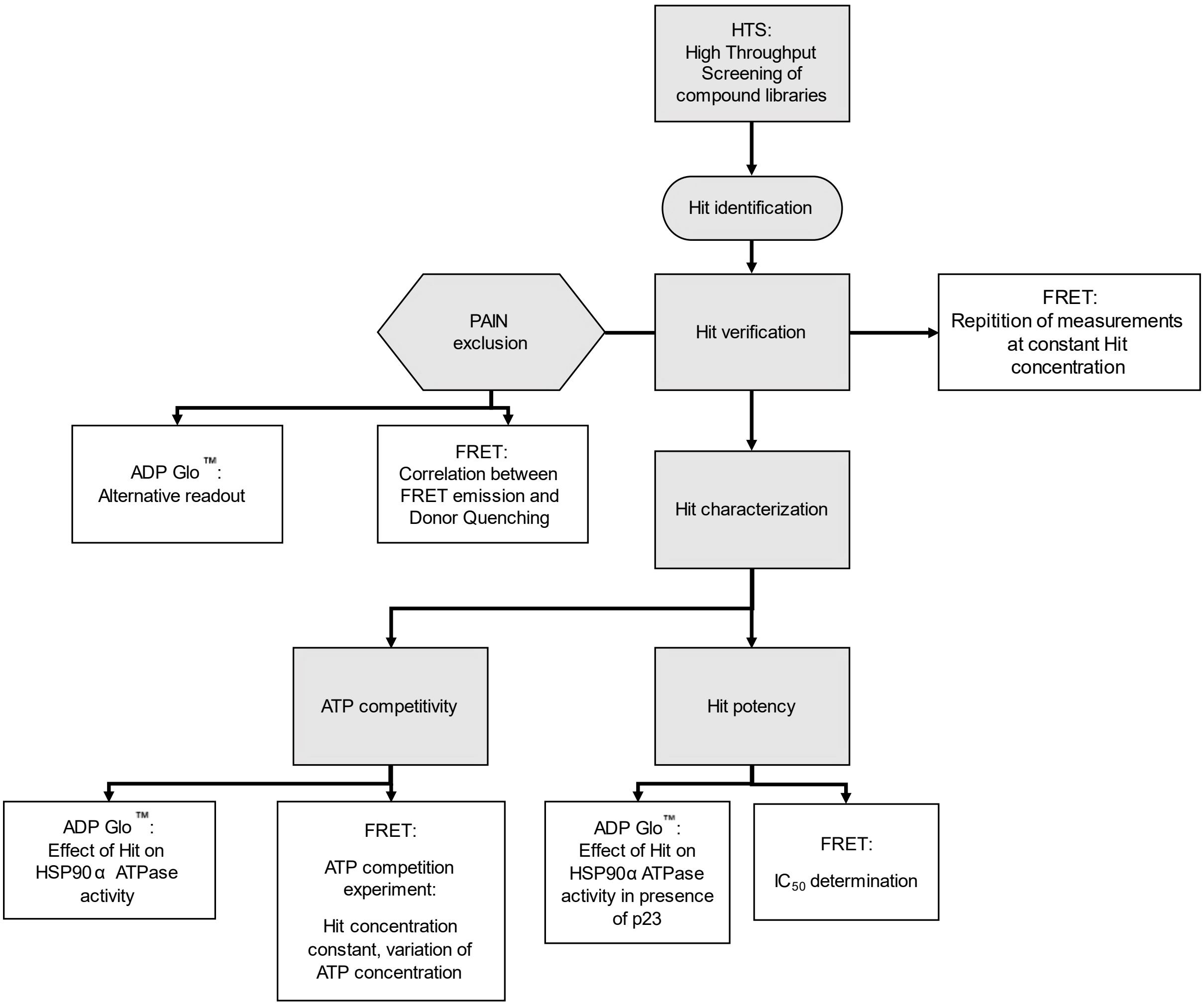

Figure S9. Workflow: Identification and profiling of inhibitors of *human* HSP90α-p23 binding.

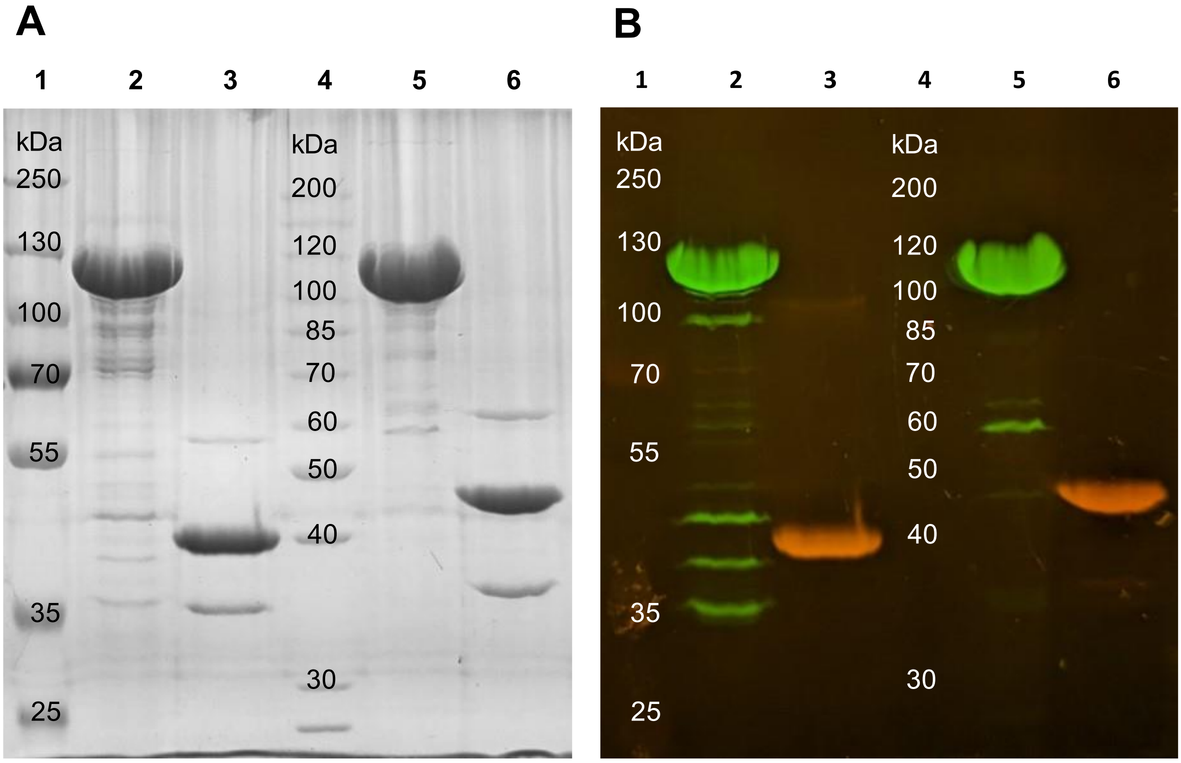

Figure S10. SDS-PAGE and purity assessment of fusion proteins for K_d experiments. Shown is the polyacrylamide gel (10%) after SDS-PAGE after Coomassie brilliant blue staining **(A)** and prior to staining under UV light **(B)** visualizing the fluorescence of the fluorescent fusion proteins. The purity of protein solutions was assessed via densitometric analysis of the Coomassie stained gel with ImageJ. The concentrations of L_0 and T_0 were corrected for the assessed purity prior to calculation of K_d. Lane 1: PAGE Ruler Prestained Plus. Lane 2: HSP90αE47A-mNeonGreen (113 kDa), purity 78.5%. Lane 3: p23-mScarlet-I (47 kDa), purity 84.1%. Lane 4: PAGE Ruler Unstained. Lane 5: HSP90E36A-mNeonGreen (109 kDa), purity 91.5%. Lane 6: Sba1-mScarlet-I (52 kDa), purity 79.5%. Due to the proteins being at least still partly folded, the migration pattern on the gel can slightly differ from their actual molecular weights.

Tables

Table S1. Characteristics of screened HSP90 inhibitors and their influence on HSP90 - Sba1/p23 binding according to literature.

**name**	**K_d value [nM]**	**ATP competitive**	**Influence on p23/Sba1 binding**	**HSP90 binding site (domain)**	**Reference(s)**
Geldanamycin	1200	Yes	Yes	NTD	[82]
Celastrol	NA	No	No	CTD	[40], [83]
Radicicol	19	Yes	Yes	NTD	[82]
NVP-AUY922 (luminespib)	1.7	Yes	Yes	NTD	[76], [84]
Snx-5422	16	Yes	Yes	NTD	[78]
BIIB021	1.7 ± 0.4 (K_i)	Yes	Yes	NTD	[85]
Silibinin	NA	NA	NA	CTD	[86]
Deguelin	NA	No	NA	CTD	[87], [88]
Withaferin A	NA	No	No	CTD	[41]
(−)-Epigallocatechin-3-gallate (EGCG)	NA	No	Yes	CTD	[42], [43]

NA: not available; NTD: N-terminal domain; CTD: C-terminal domain

Table S2. Compound list of nucleoside-mimetic library

#	Structure
1	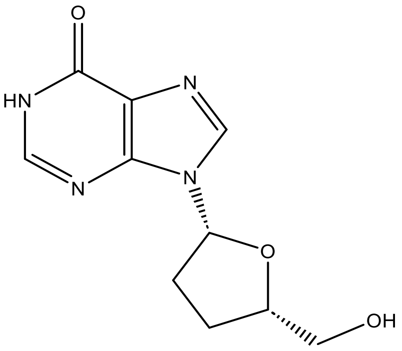<br>OC[C@@H]1CC[C@@H](O1)N2C=NC=3C(=O)NC=NC23
2	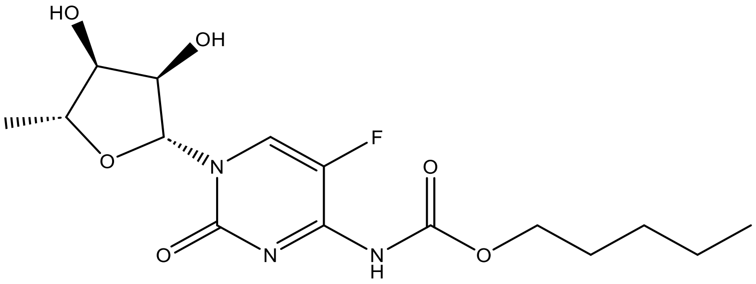<br>CCCCCOC(=O)NC1=NC(=O)N(C=C1F)[C@@H]2O[C@H](C)[C@@H](O)[C@H]2O
3	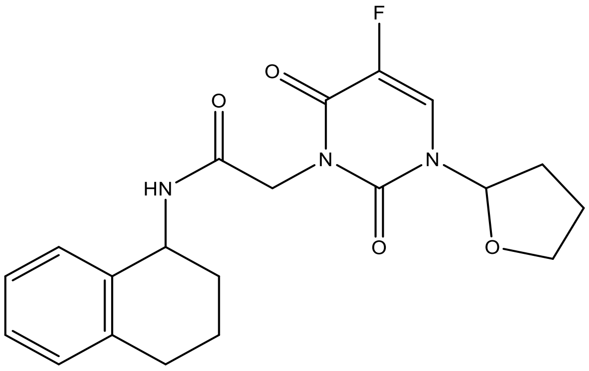<br>FC1=CN(C2CCCO2)C(=O)N(CC(=O)NC3CCCC=4C=CC=CC34)C1=O
4	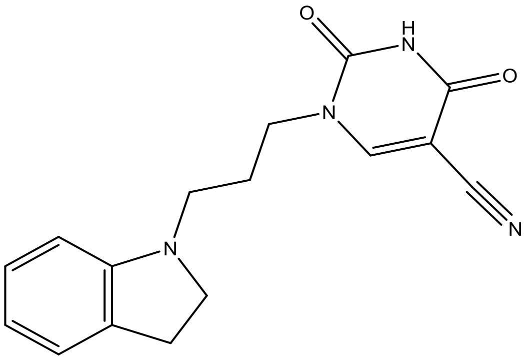<br>O=C1NC(=O)N(CCCN2CCC=3C=CC=CC23)C=C1C#N
5	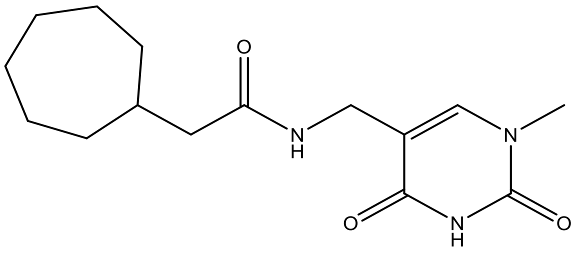<br>CN1C=C(CNC(=O)CC2CCCCCC2)C(=O)NC1=O

6	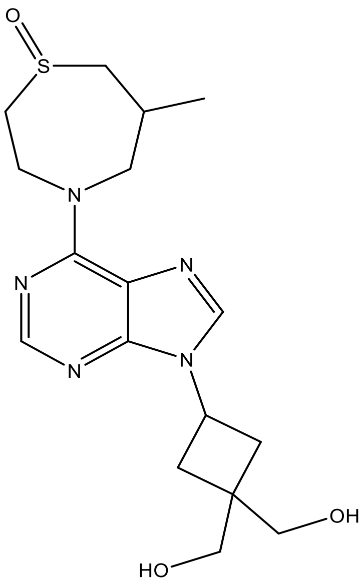<br>CC1CN(CCS(=O)C1)C=2N=CN=C3N(C=NC23)C4CC(CO)(CO)C4
7	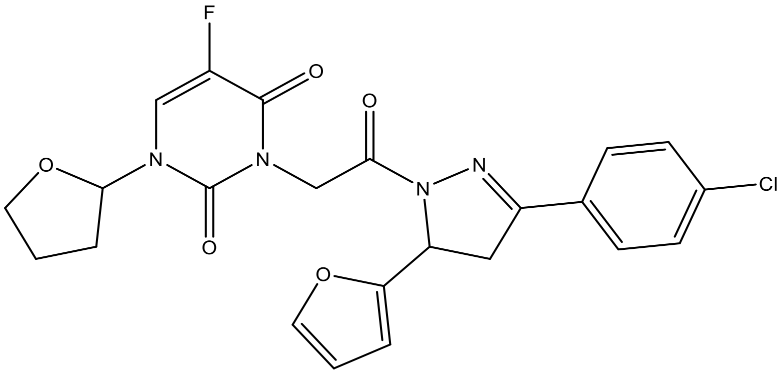<br>FC1=CN(C2CCCO2)C(=O)N(CC(=O)N3N=C(CC3C4=CC=CO4)C=5C=CC(Cl)=CC5)C1=O
8	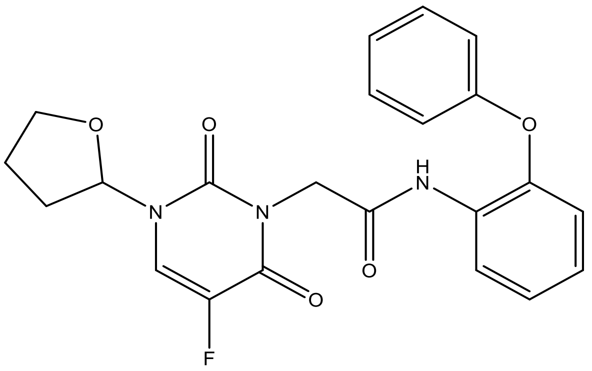<br>FC1=CN(C2CCCO2)C(=O)N(CC(=O)NC=3C=CC=CC3OC=4C=CC=CC4)C1=O
9	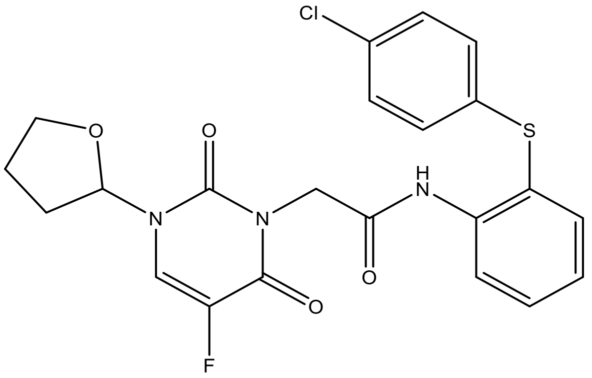<br>FC1=CN(C2CCCO2)C(=O)N(CC(=O)NC=3C=CC=CC3SC=4C=CC(Cl)=CC4)C1=O
10	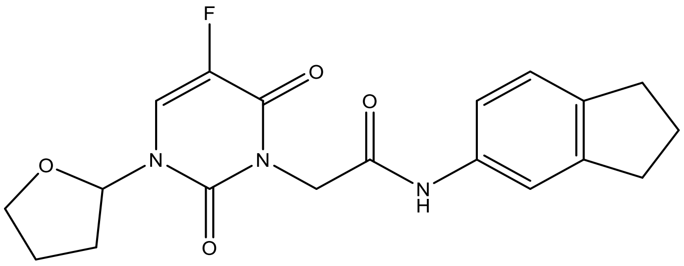<br>FC1=CN(C2CCCO2)C(=O)N(CC(=O)NC=3C=CC=4CCCC4C3)C1=O

11	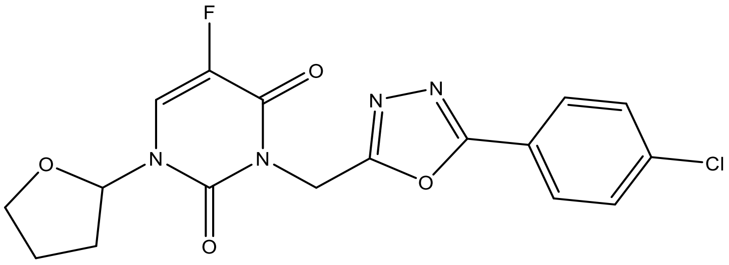<br>FC1=CN(C2CCCO2)C(=O)N(CC3=NN=C(O3)C=4C=CC(Cl)=CC4)C1=O
12	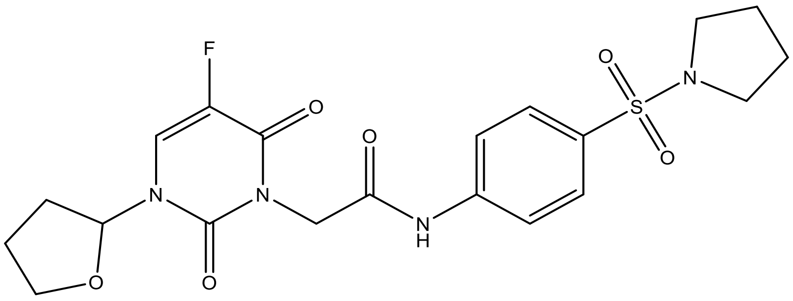<br>FC1=CN(C2CCCO2)C(=O)N(CC(=O)NC=3C=CC(=CC3)S(=O)(=O)N4CCCC4)C1=O
13	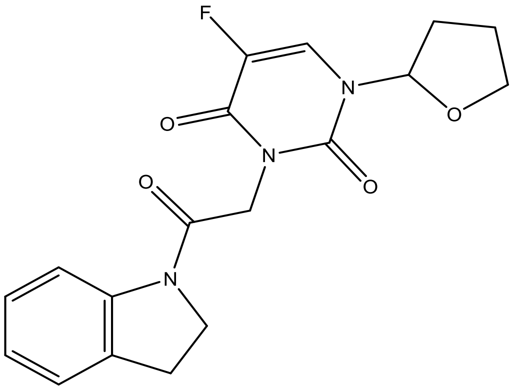<br>FC1=CN(C2CCCO2)C(=O)N(CC(=O)N3CCC=4C=CC=CC34)C1=O
14	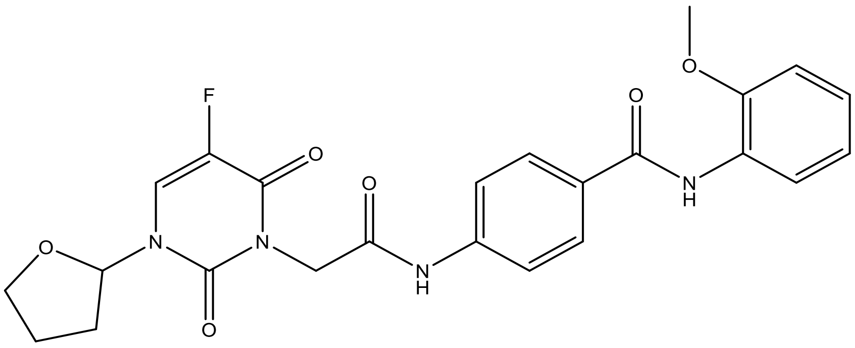<br>COC=1C=CC=CC1NC(=O)C=2C=CC(NC(=O)CN3C(=O)C(F)=CN(C4CCCO4)C3=O)=CC2
15	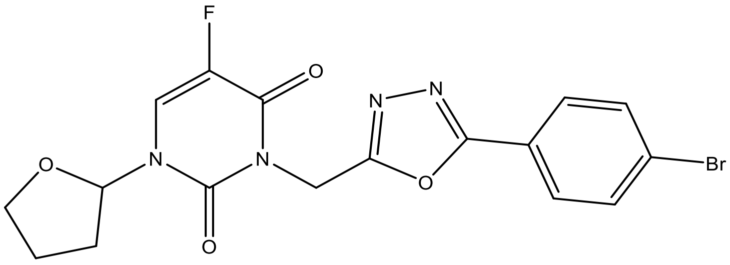<br>FC1=CN(C2CCCO2)C(=O)N(CC3=NN=C(O3)C=4C=CC(Br)=CC4)C1=O
16	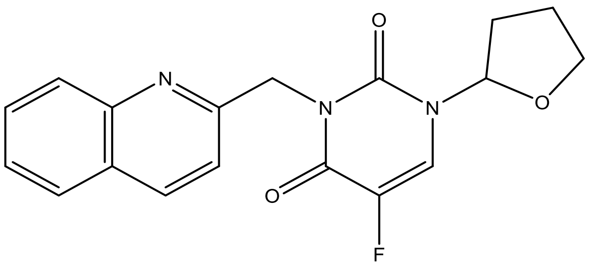<br>FC1=CN(C2CCCO2)C(=O)N(CC=3C=CC=4C=CC=CC4N3)C1=O
17	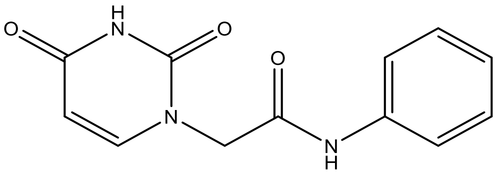<br>O=C(CN1C=CC(=O)NC1=O)NC=2C=CC=CC2

18	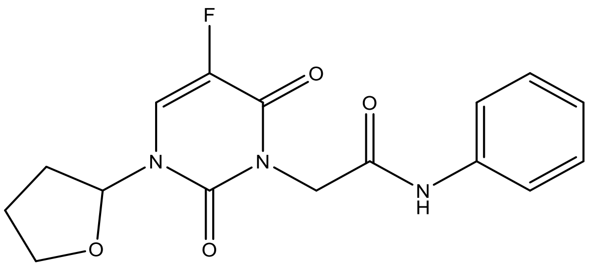 FC1=CN(C2CCCO2)C(=O)N(CC(=O)NC=3C=CC=CC3)C1=O
19	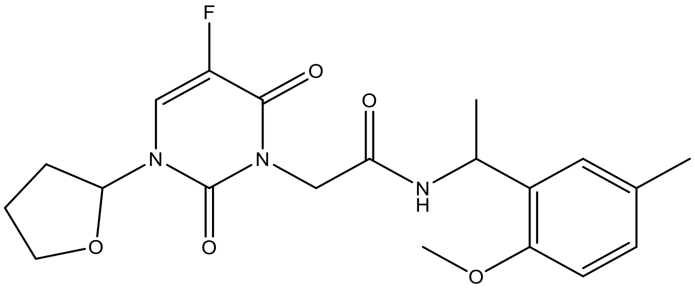 COC=1C=CC(C)=CC1C(C)NC(=O)CN2C(=O)C(F)=CN(C3CCCO3)C2=O
20	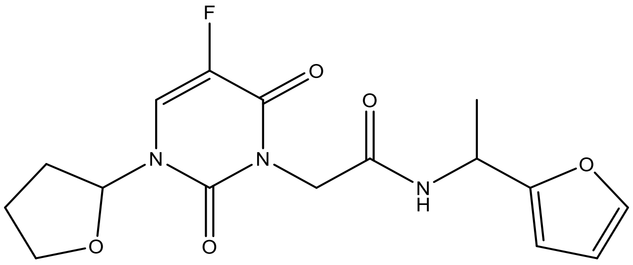 CC(NC(=O)CN1C(=O)C(F)=CN(C2CCCO2)C1=O)C3=CC=CO3
21	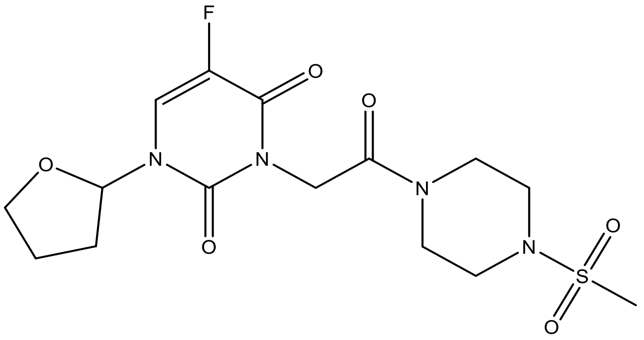 CS(=O)(=O)N1CCN(CC1)C(=O)CN2C(=O)C(F)=CN(C3CCCO3)C2=O
22	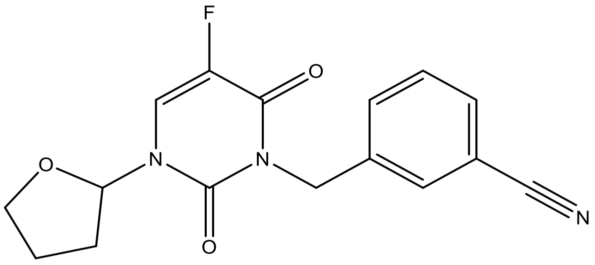 FC1=CN(C2CCCO2)C(=O)N(CC=3C=CC=C(C#N)C3)C1=O
23	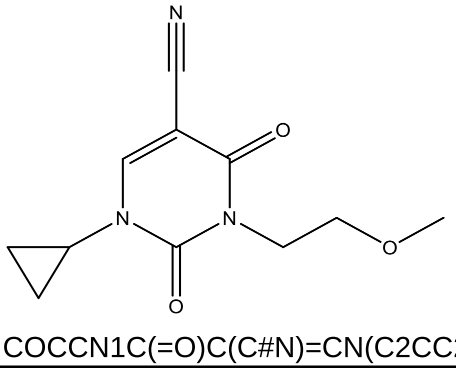 COCCN1C(=O)C(C#N)=CN(C2CC2)C1=O
24	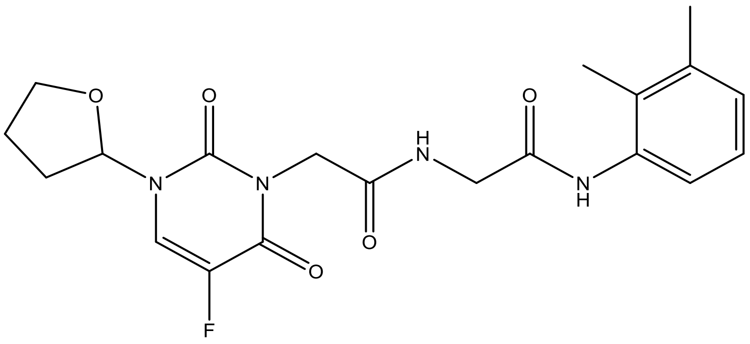 CC=1C=CC=C(NC(=O)CNC(=O)CN2C(=O)C(F)=CN(C3CCCO3)C2=O)C1C

25	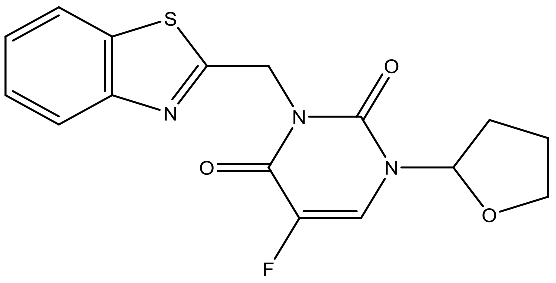<br>FC1=CN(C2CCCO2)C(=O)N(CC3=NC=4C=CC=CC4S3)C1=O
26	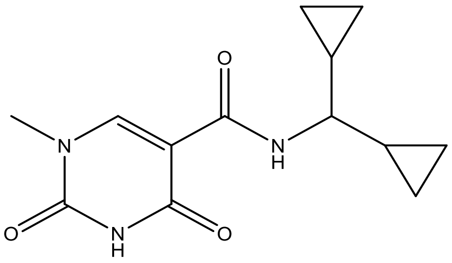<br>CN1C=C(C(=O)NC(C2CC2)C3CC3)C(=O)NC1=O
27	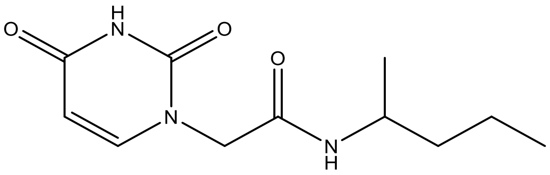<br>CCCC(C)NC(=O)CN1C=CC(=O)NC1=O
28	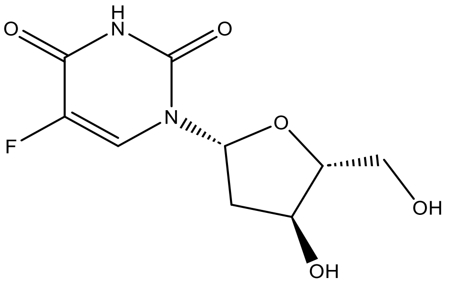<br>OC[C@H]1O[C@H](C[C@@H]1O)N2C=C(F)C(=O)NC2=O
29	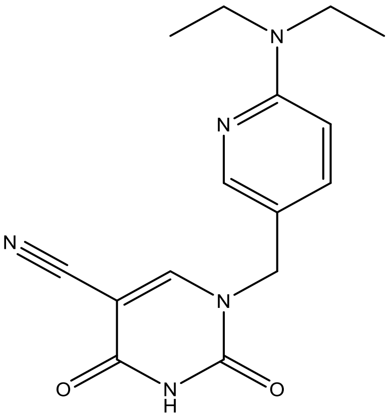<br>CCN(CC)C=1C=CC(CN2C=C(C#N)C(=O)NC2=O)=CN1
30	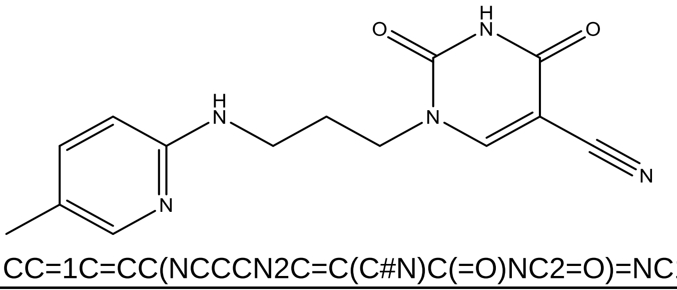<br>CC=1C=CC(NCCCN2C=C(C#N)C(=O)NC2=O)=NC1
31	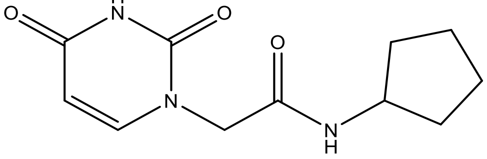<br>O=C(CN1C=CC(=O)NC1=O)NC2CCCC2
32	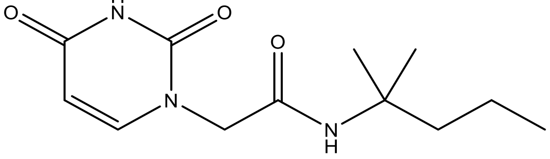<br>CCCC(C)(C)NC(=O)CN1C=CC(=O)NC1=O

33	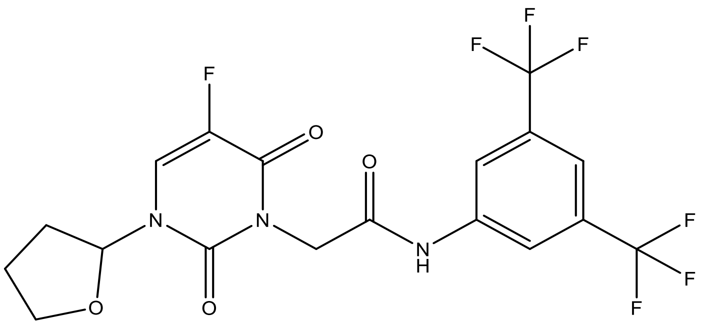 FC1=CN(C2CCCO2)C(=O)N(CC(=O)NC=3C=C(C=C(C3)C(F)(F)F)C(F)(F)F)C1=O
34	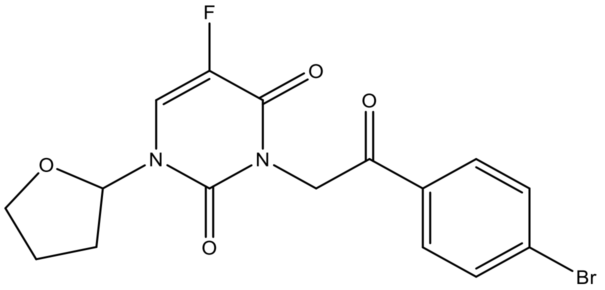 FC1=CN(C2CCCO2)C(=O)N(CC(=O)C=3C=CC(Br)=CC3)C1=O
35	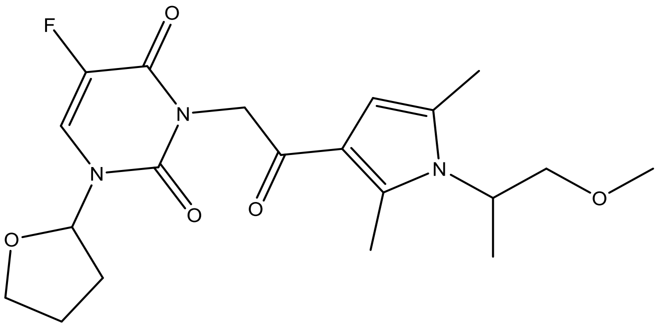 COCC(C)N1C(C)=CC(C(=O)CN2C(=O)C(F)=CN(C3CCCO3)C2=O)=C1C
36	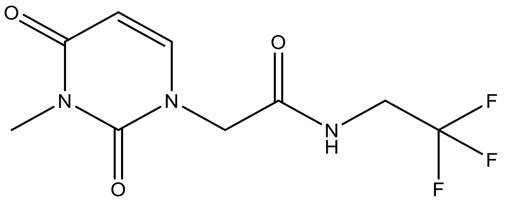 CN1C(=O)C=CN(CC(=O)NCC(F)(F)F)C1=O
37	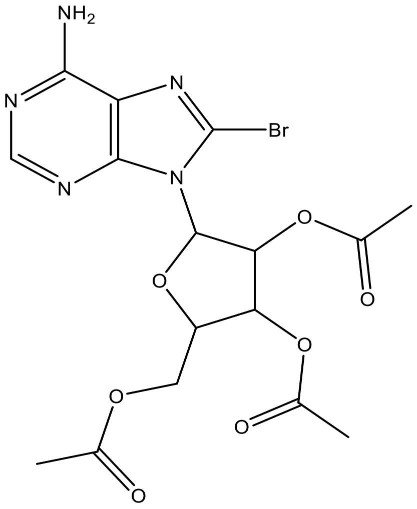 CC(=O)OCC1OC(C(OC(=O)C)C1OC(=O)C)N2C(Br)=NC=3C(N)=NC=NC23

38	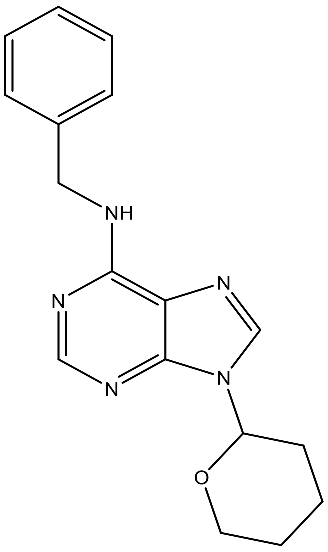<br>C(NC=1N=CN=C2N(C=NC12)C3CCCCO3)C=4C=CC=CC4
39	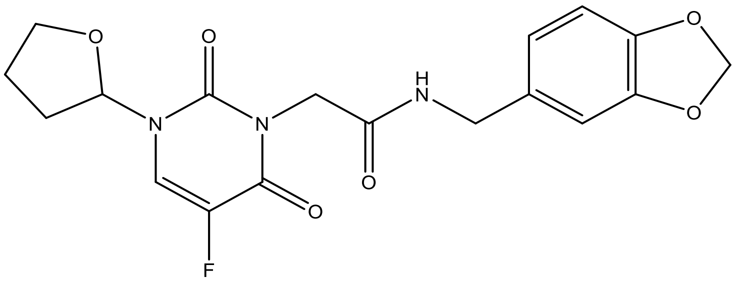<br>FC1=CN(C2CCCO2)C(=O)N(CC(=O)NCC=3C=CC=4OCOC4C3)C1=O
40	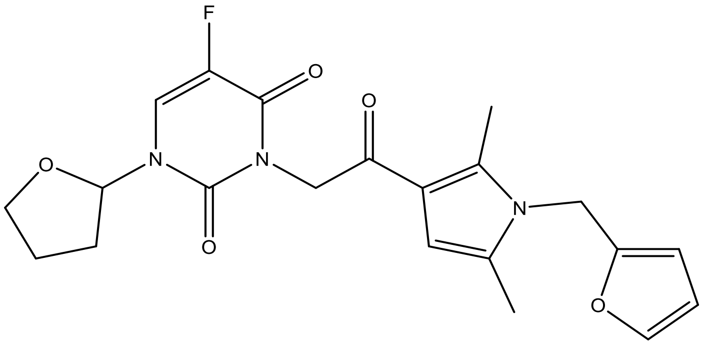<br>CC1=CC(C(=O)CN2C(=O)C(F)=CN(C3CCCO3)C2=O)=C(C)N1CC4=CC=CO4
41	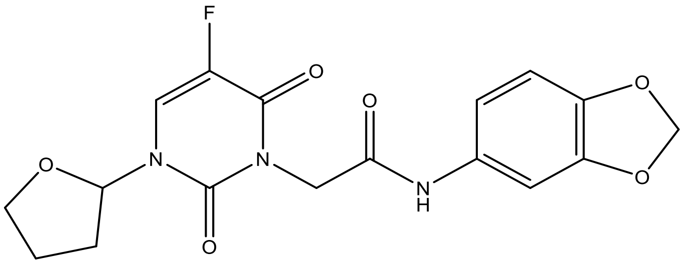<br>FC1=CN(C2CCCO2)C(=O)N(CC(=O)NC=3C=CC=4OCOC4C3)C1=O
42	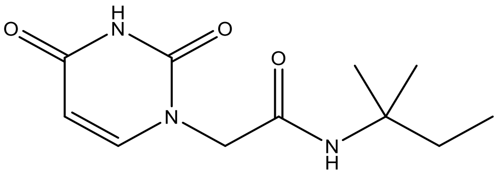<br>CCC(C)(C)NC(=O)CN1C=CC(=O)NC1=O
43	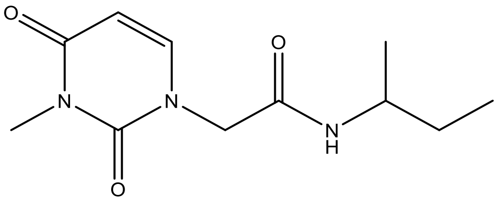<br>CCC(C)NC(=O)CN1C=CC(=O)N(C)C1=O

44	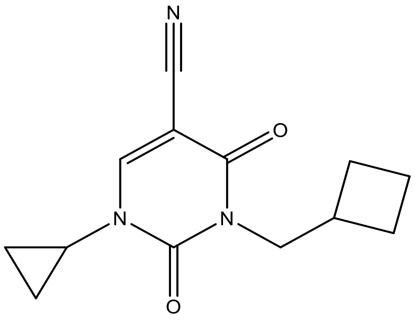<br>O=C1C(C#N)=CN(C2CC2)C(=O)N1CC3CCC3
45	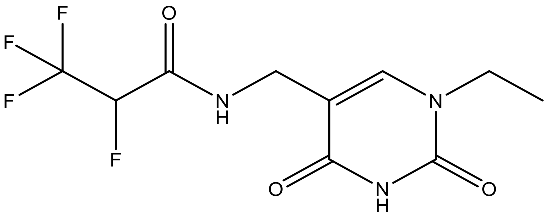<br>CCN1C=C(CNC(=O)C(F)C(F)(F)F)C(=O)NC1=O
46	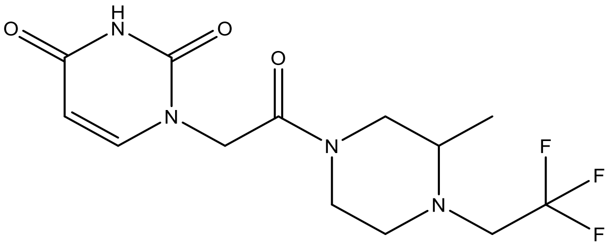<br>CC1CN(CCN1CC(F)(F)F)C(=O)CN2C=CC(=O)NC2=O
47	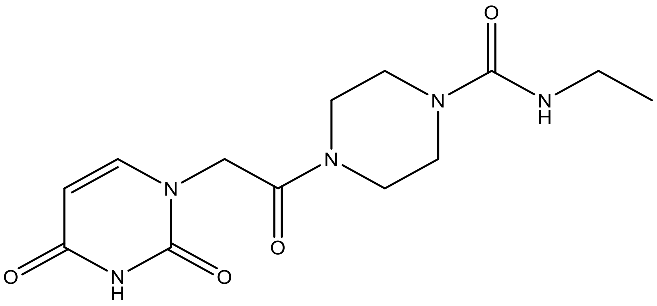<br>CCNC(=O)N1CCN(CC1)C(=O)CN2C=CC(=O)NC2=O
48	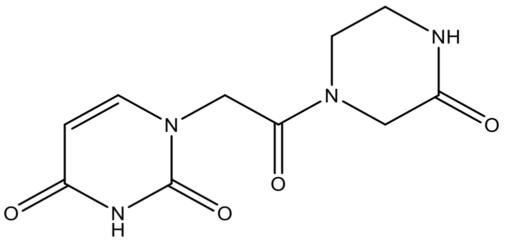<br>O=C(CN1C=CC(=O)NC1=O)N2CCNC(=O)C2
49	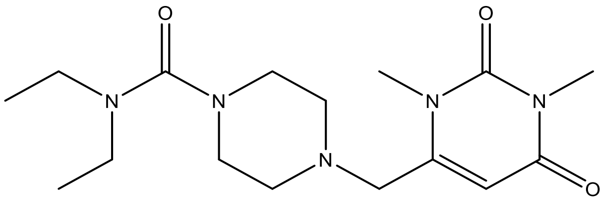<br>CCN(CC)C(=O)N1CCN(CC2=CC(=O)N(C)C(=O)N2C)CC1
50	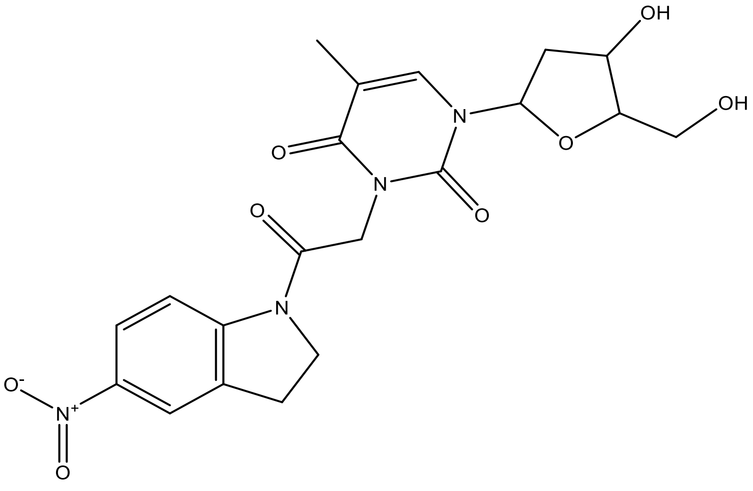<br>CC1=CN(C2CC(O)C(CO)O2)C(=O)N(CC(=O)N3CCC=4C=C(C=CC34)[N+](=O)[O-])C1=O

51	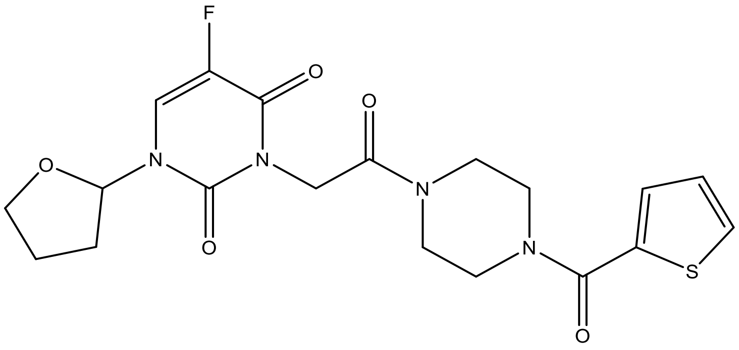<br>FC1=CN(C2CCCO2)C(=O)N(CC(=O)N3CCN(CC3)C(=O)C4=CC=CS4)C1=O
52	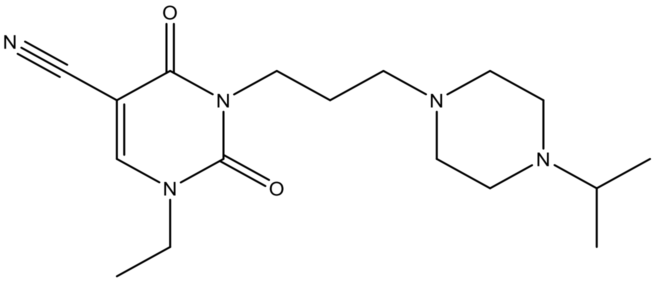<br>CCN1C=C(C#N)C(=O)N(CCCN2CCN(CC2)C(C)C)C1=O
53	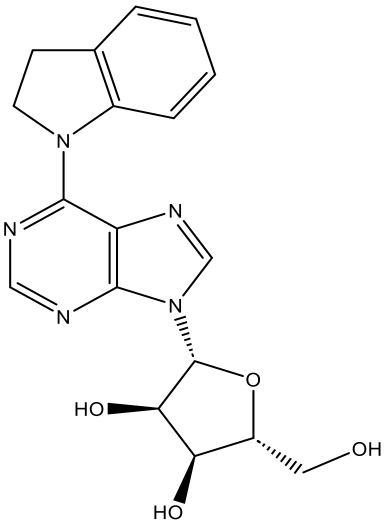<br>OC[C@H]1O[C@H]([C@H](O)[C@@H]1O)N2C=NC=3C(=NC=NC23)N4CCC=5C=CC=CC45
54	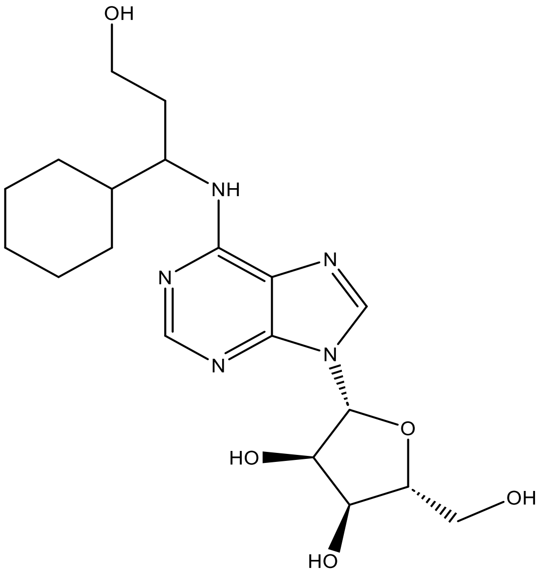<br>OCCC(NC=1N=CN=C2N(C=NC12)[C@@H]3O[C@H](CO)[C@@H](O)[C@H]3O)C4CCCCC4
55	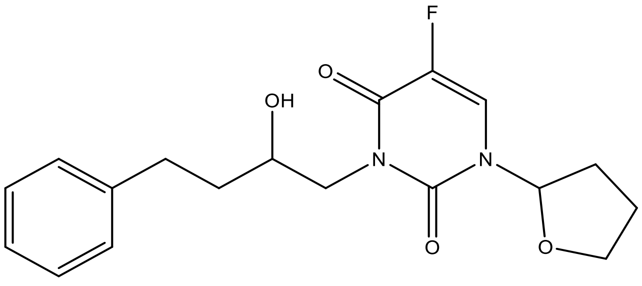<br>OC(CCC=1C=CC=CC1)CN2C(=O)C(F)=CN(C3CCCO3)C2=O

	56	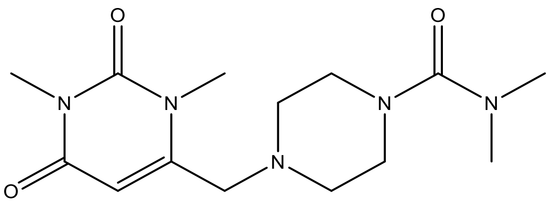 CN(C)C(=O)N1CCN(CC2=CC(=O)N(C)C(=O)N2C)CC1
	57	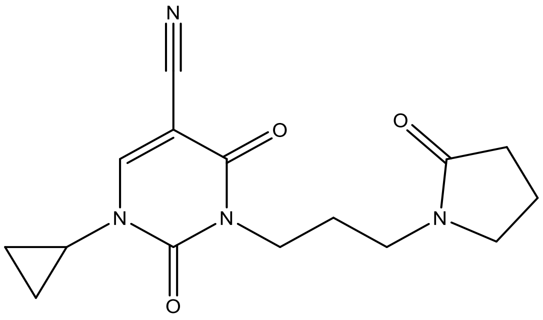 O=C1CCCN1CCCN2C(=O)C(C#N)=CN(C3CC3)C2=O
	58	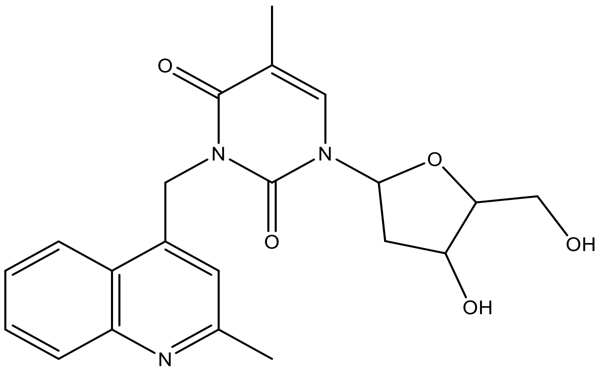 CC=1C=C(CN2C(=O)C(C)=CN(C3CC(O)C(CO)O3)C2=O)C=4C=CC=CC4N1
	59	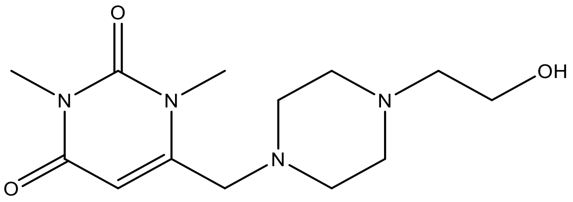 CN1C(CN2CCN(CCO)CC2)=CC(=O)N(C)C1=O
	60	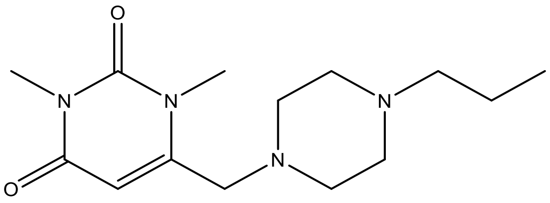 CCCN1CCN(CC2=CC(=O)N(C)C(=O)N2C)CC1
	61	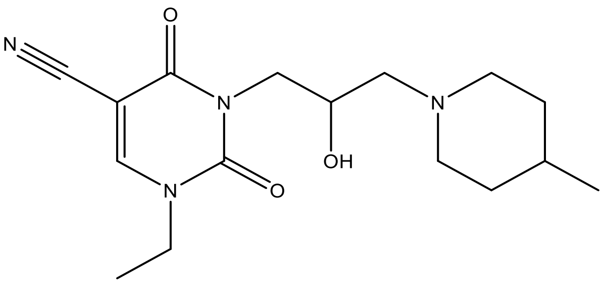 CCN1C=C(C#N)C(=O)N(CC(O)CN2CCC(C)CC2)C1=O
	62	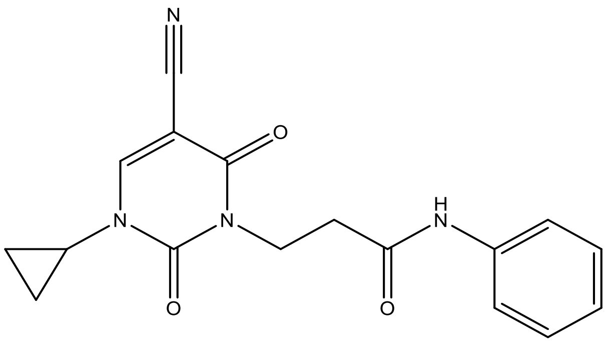 O=C(CCN1C(=O)C(C#N)=CN(C2CC2)C1=O)NC=3C=CC=CC3

63	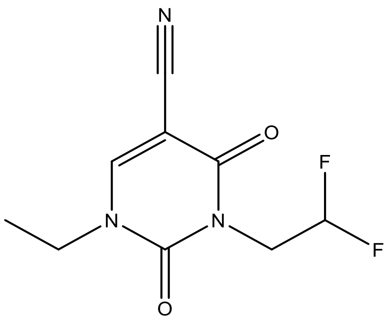<br>CCN1C=C(C#N)C(=O)N(CC(F)F)C1=O
64	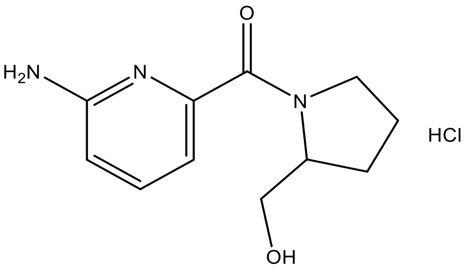<br>Cl.NC=1C=CC=C(N1)C(=O)N2CCCC2CO
65	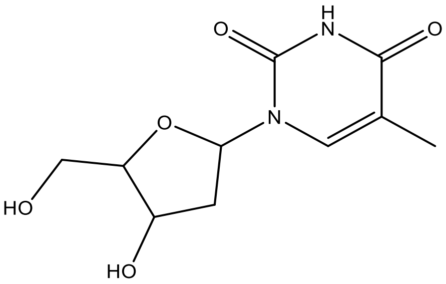<br>CC1=CN(C2CC(O)C(CO)O2)C(=O)NC1=O
66	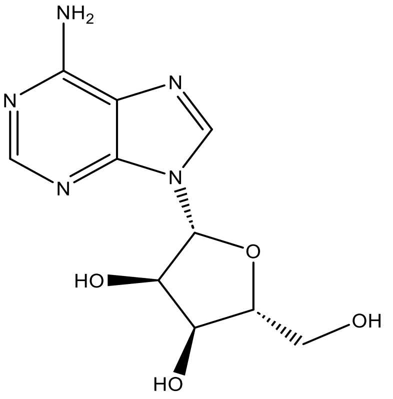<br>NC=1N=CN=C2N(C=NC12)[C@@H]3O[C@H](CO)[C@@H](O)[C@H]3O
67	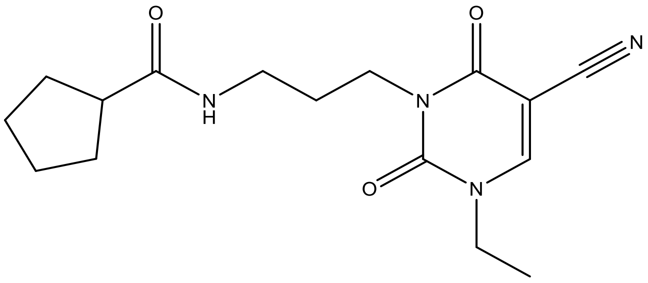<br>CCN1C=C(C#N)C(=O)N(CCCNC(=O)C2CCCC2)C1=O
68	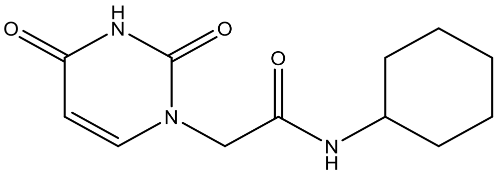<br>O=C(CN1C=CC(=O)NC1=O)NC2CCCCC2
69	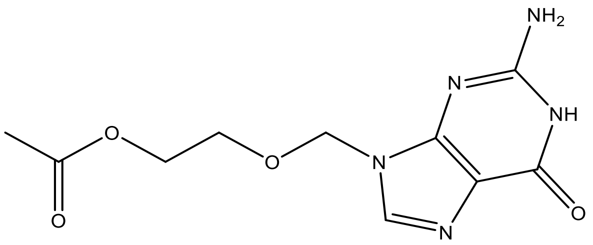<br>CC(=O)OCCOCN1C=NC=2C(=O)NC(N)=NC12

70	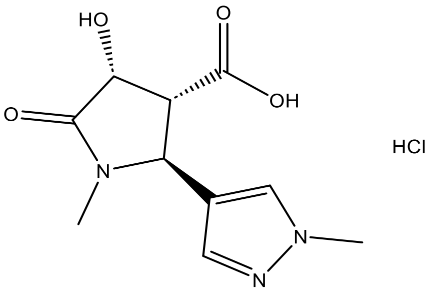<br>Cl.CN1[C@@H]([C@H]([C@@H](O)C1=O)C(=O)O)C=2C=NN(C)C2
71	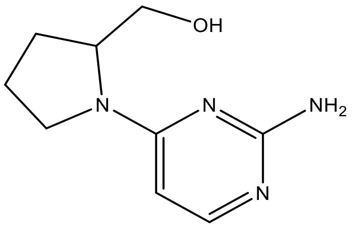<br>NC=1N=CC=C(N1)N2CCCC2CO
72	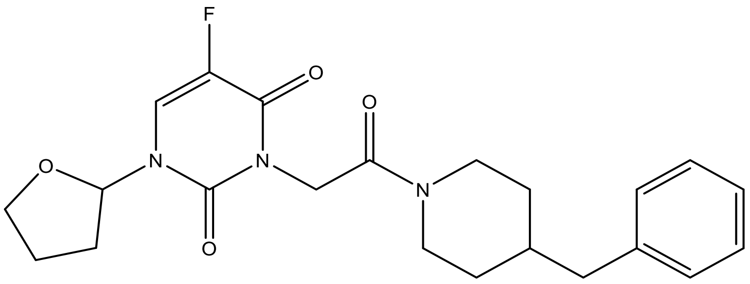<br>FC1=CN(C2CCCO2)C(=O)N(CC(=O)N3CCC(CC=4C=CC=CC4)CC3)C1=O
73	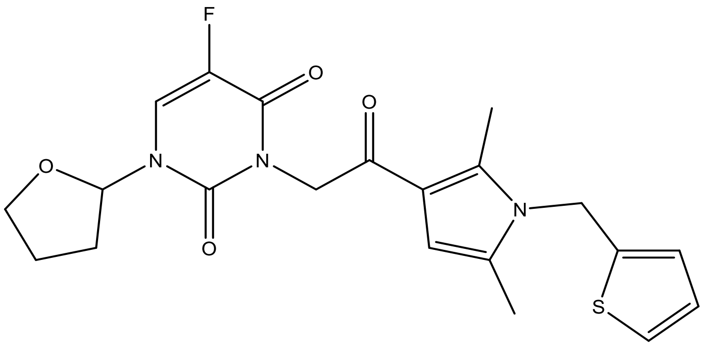<br>CC1=CC(C(=O)CN2C(=O)C(F)=CN(C3CCCO3)C2=O)=C(C)N1CC4=CC=CS4
74	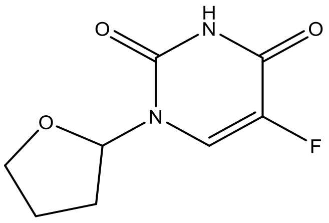<br>FC1=CN(C2CCCO2)C(=O)NC1=O
75	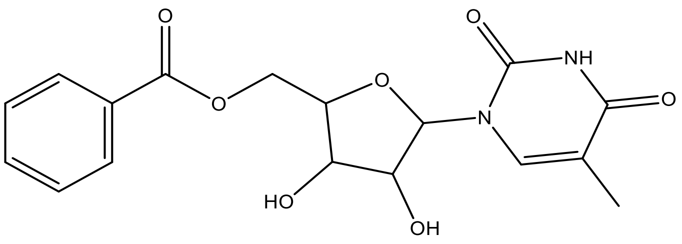<br>CC1=CN(C2OC(COC(=O)C=3C=CC=CC3)C(O)C2O)C(=O)NC1=O
76	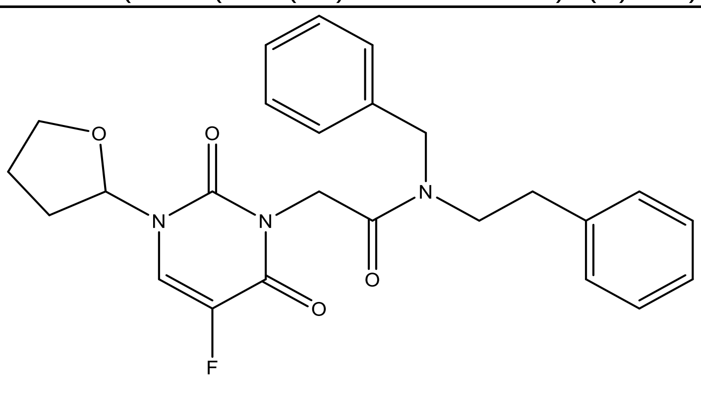<br>FC1=CN(C2CCCO2)C(=O)N(CC(=O)N(CCC=3C=CC=CC3)CC=4C=CC=CC4)C1=O

77	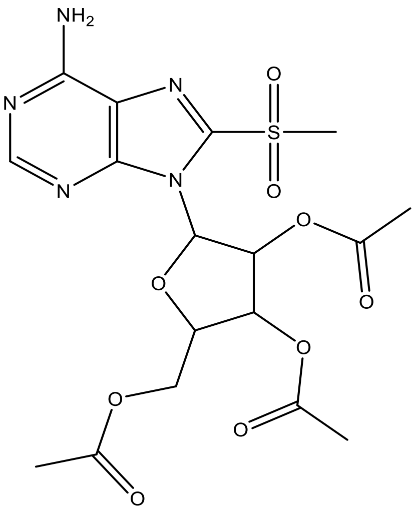 CC(=O)OCC1OC(C(OC(=O)C)C1OC(=O)C)N2C(=NC=3C(N)=NC=NC23)S(=O)(=O)C
78	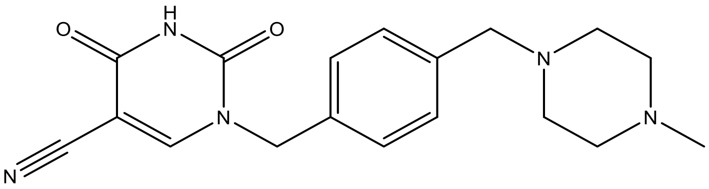 CN1CCN(CC=2C=CC(CN3C=C(C#N)C(=O)NC3=O)=CC2)CC1
79	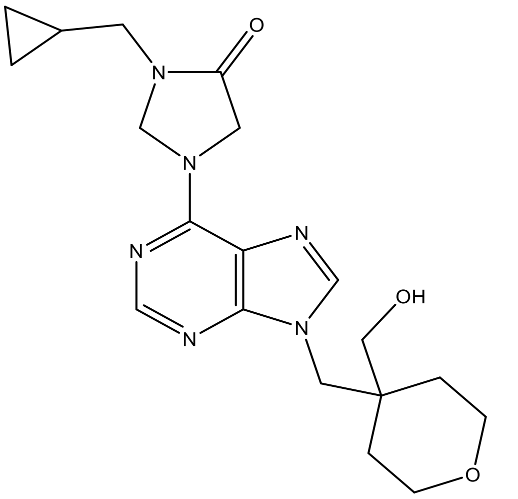 OCC1(CN2C=NC=3C(=NC=NC23)N4CN(CC5CC5)C(=O)C4)CCOCC1
80	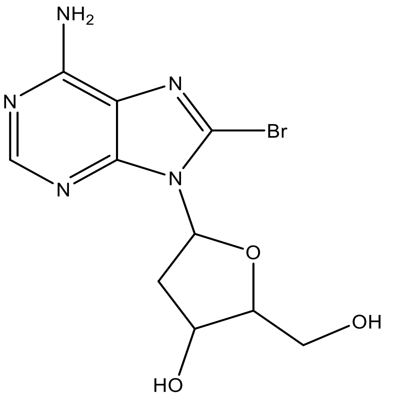 NC=1N=CN=C2N(C3CC(O)C(CO)O3)C(Br)=NC12
81	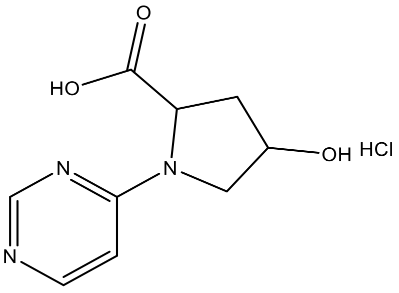 Cl.OC1CC(N(C1)C=2C=CN=CN2)C(=O)O
82	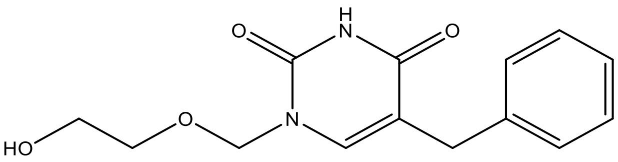 OCCOCN1C=C(CC=2C=CC=CC2)C(=O)NC1=O

83	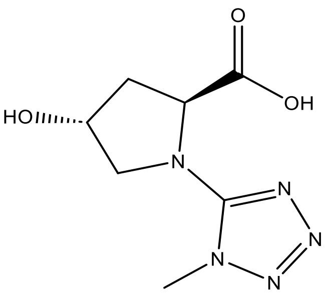<br>CN1N=NN=C1N2C[C@H](O)C[C@H]2C(=O)O
84	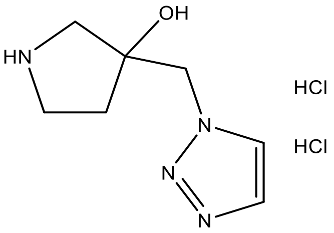<br>Cl.Cl.OC1(CN2C=CN=N2)CCNC1
85	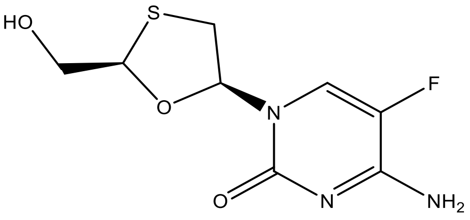<br>NC1=NC(=O)N(C=C1F)[C@@H]2CS[C@H](CO)O2
86	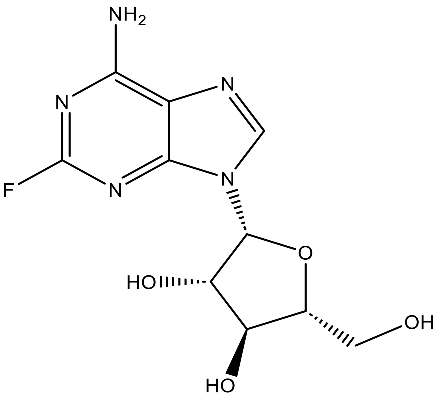<br>NC=1N=C(F)N=C2N(C=NC12)[C@@H]3O[C@H](CO)[C@@H](O)[C@@H]3O
87	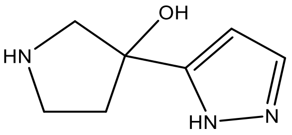<br>OC1(CCNC1)C2=CC=NN2
88	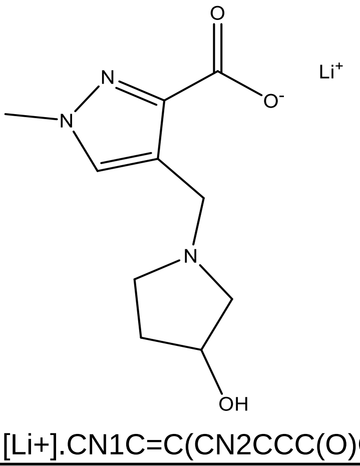<br>[Li+].CN1C=C(CN2CCC(O)C2)C(=N1)C(=O)[O-]
89	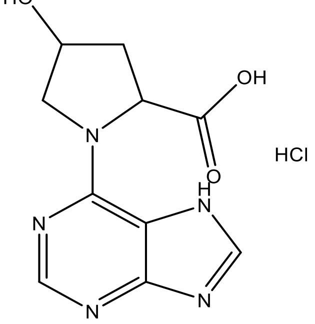<br>Cl.OC1CC(N(C1)C=2N=CN=C3N=CNC23)C(=O)O

90	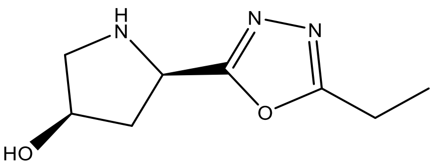<br>CCC1=NN=C(O1)[C@H]2C[C@@H](O)CN2
91	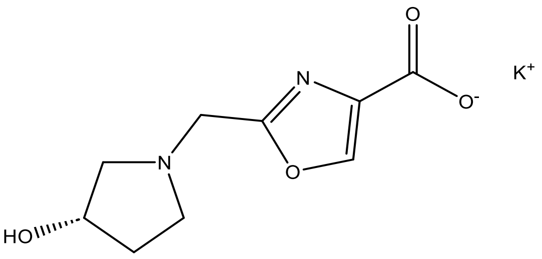<br>[K+].O[C@H]1CCN(CC2=NC(=CO2)C(=O)[O-])C1
92	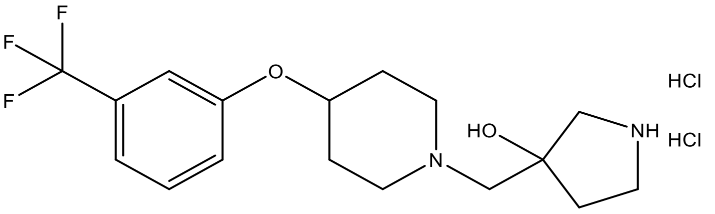<br>Cl.Cl.OC1(CN2CCC(CC2)OC=3C=CC=C(C3)C(F)(F)F)CCNC1
93	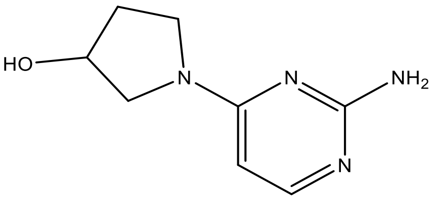<br>NC=1N=CC=C(N1)N2CCC(O)C2
94	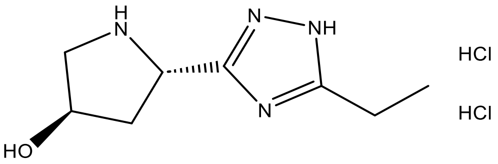<br>Cl.Cl.CCC1=NC(=NN1)[C@@H]2C[C@@H](O)CN2
95	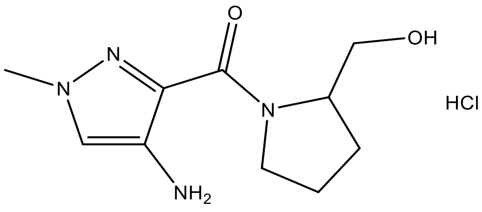<br>Cl.CN1C=C(N)C(=N1)C(=O)N2CCCC2CO
96	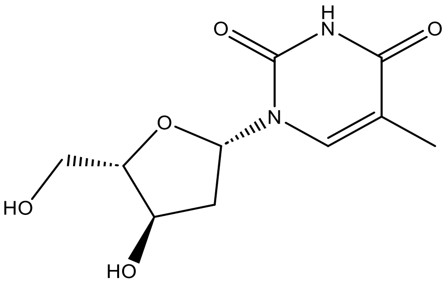<br>CC1=CN([C@@H]2C[C@@H](O)[C@H](CO)O2)C(=O)NC1=O
97	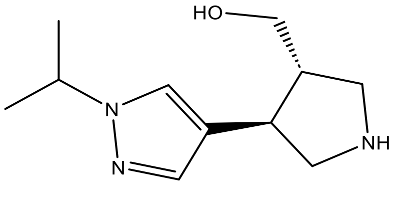<br>CC(C)N1C=C(C=N1)[C@@H]2CNC[C@H]2CO
98	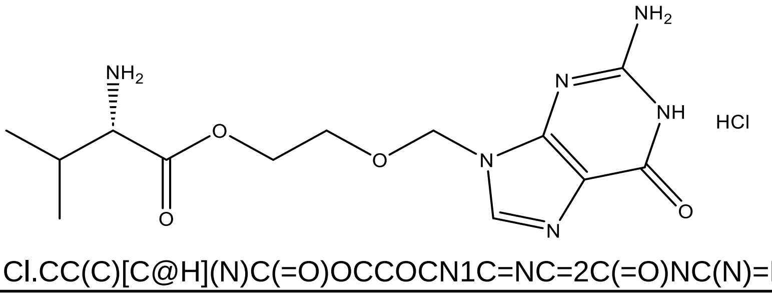<br>Cl.CC(C)[C@H](N)C(=O)OCCOCN1C=NC=2C(=O)NC(N)=NC12

99	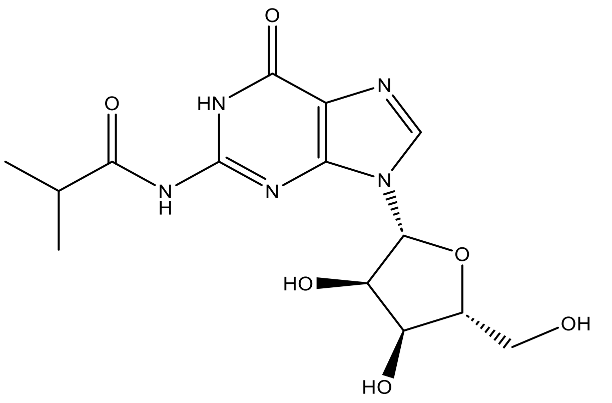<br>CC(C)C(=O)NC1=NC=2N(C=NC2C(=O)N1)[C@@H]3O[C@H](CO)[C@@H](O)[C@H]3O
100	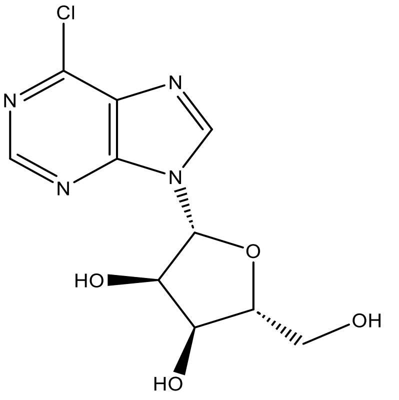<br>OC[C@H]1O[C@H]([C@H](O)[C@@H]1O)N2C=NC=3C(Cl)=NC=NC23
101	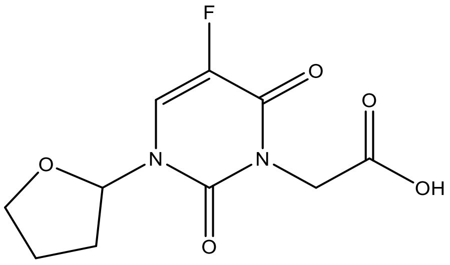<br>OC(=O)CN1C(=O)C(F)=CN(C2CCCO2)C1=O
102	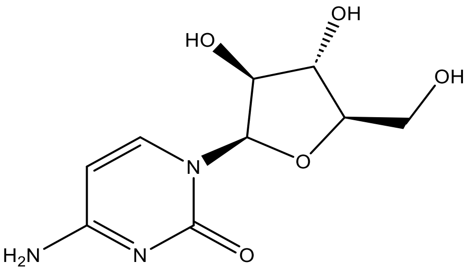<br>NC=1C=CN([C@@H]2O[C@H](CO)[C@@H](O)[C@@H]2O)C(=O)N1
103	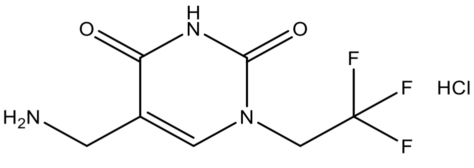<br>Cl.NCC1=CN(CC(F)(F)F)C(=O)NC1=O
104	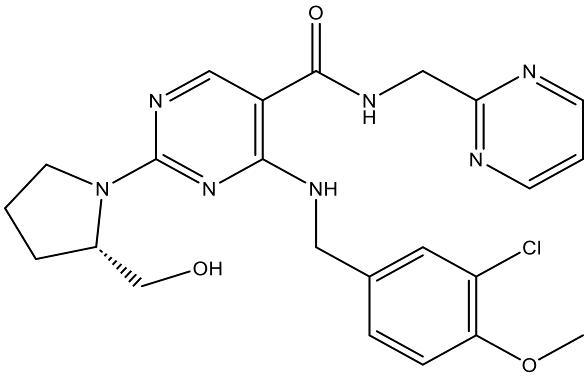<br>COC=1C=CC(CNC=2N=C(N=CC2C(=O)NCC=3N=CC=CN3)N4CCC[C@H]4CO)=CC1Cl
105	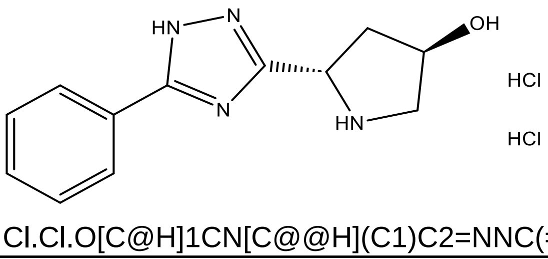<br>Cl.Cl.O[C@H]1CN[C@@H](C1)C2=NNC(=N2)C=3C=CC=CC3

106	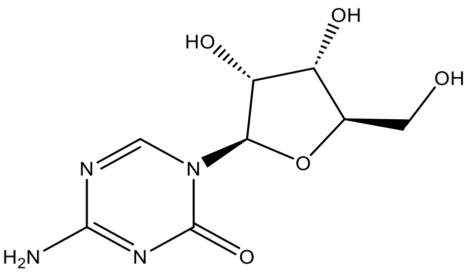 NC=1N=CN([C@@H]2O[C@H](CO)[C@@H](O)[C@H]2O)C(=O)N1
107	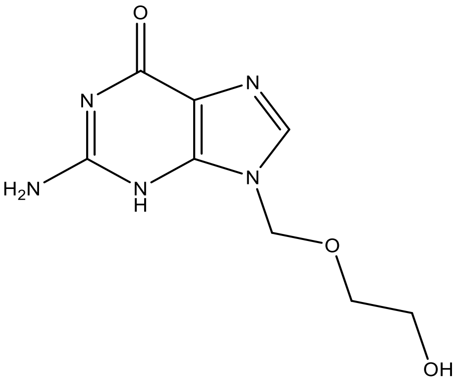 NC1=NC(=O)C=2N=CN(COCCO)C2N1
108	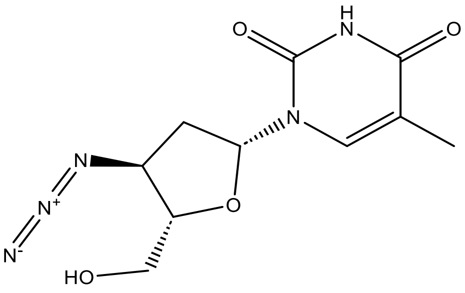 CC1=CN([C@H]2C[C@H](N=[N+]=[N-])[C@@H](CO)O2)C(=O)NC1=O
109	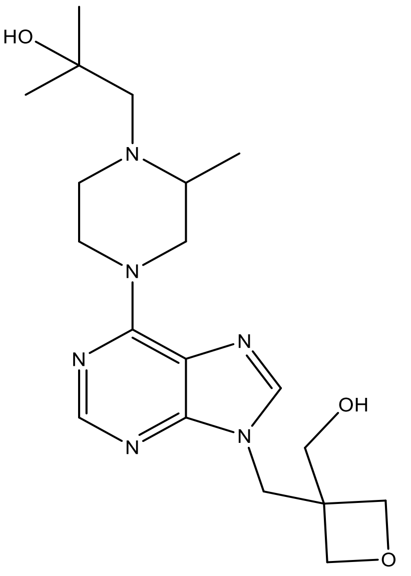 CC1CN(CCN1CC(C)(C)O)C=2N=CN=C3N(CC4(CO)COC4)C=NC23
110	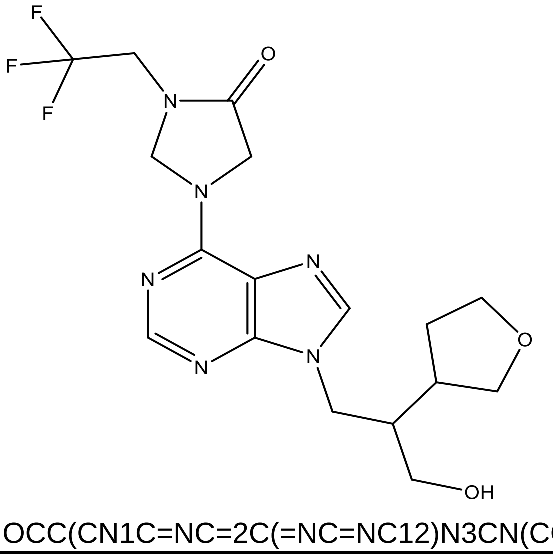 OCC(CN1C=NC=2C(=NC=NC12)N3CN(CC(F)(F)F)C(=O)C3)C4CCOC4

111	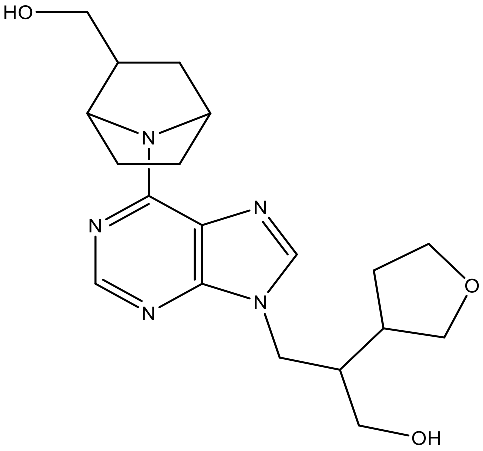<br>OCC(CN1C=NC=2C(=NC=NC12)N3C4CCC3C(CO)C4)C5CCOC5
112	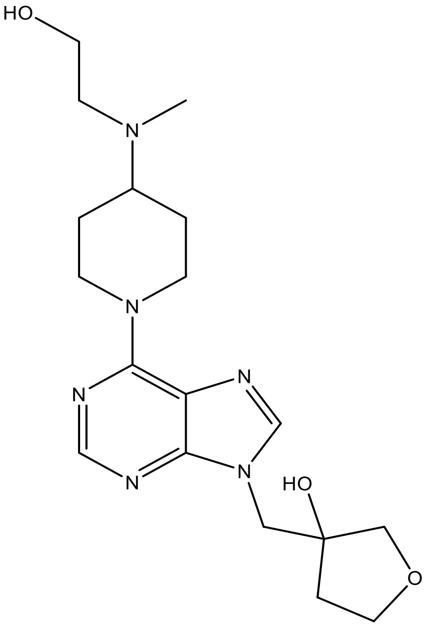<br>CN(CCO)C1CCN(CC1)C=2N=CN=C3N(CC4(O)CCOC4)C=NC23
113	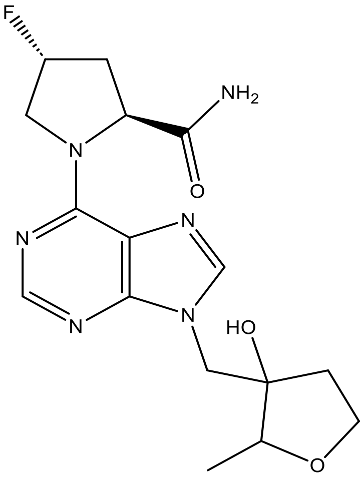<br>CC1OCCC1(O)CN2C=NC=3C(=NC=NC23)N4C[C@H](F)C[C@H]4C(=O)N
114	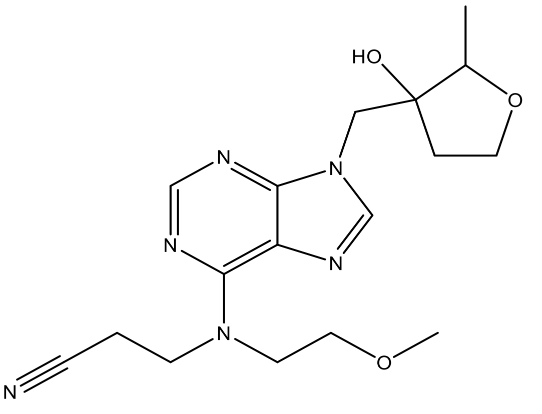<br>COCCN(CCC#N)C=1N=CN=C2N(CC3(O)CCOC3C)C=NC12
115	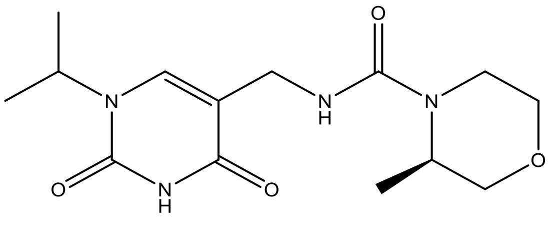<br>CC(C)N1C=C(CNC(=O)N2CCOC[C@H]2C)C(=O)NC1=O

	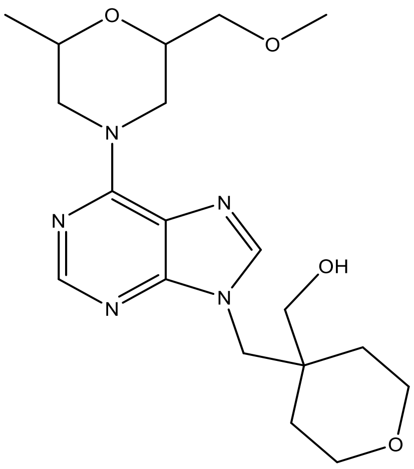
116	COCC1CN(CC(C)O1)C=2N=CN=C3N(CC4(CO)CCOCC4)C=NC23
	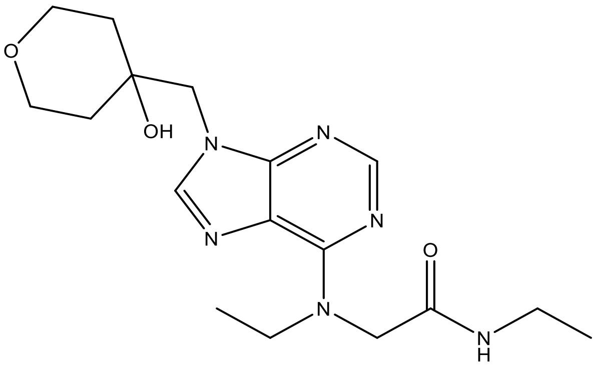
117	CCNC(=O)CN(CC)C=1N=CN=C2N(CC3(O)CCOCC3)C=NC12
	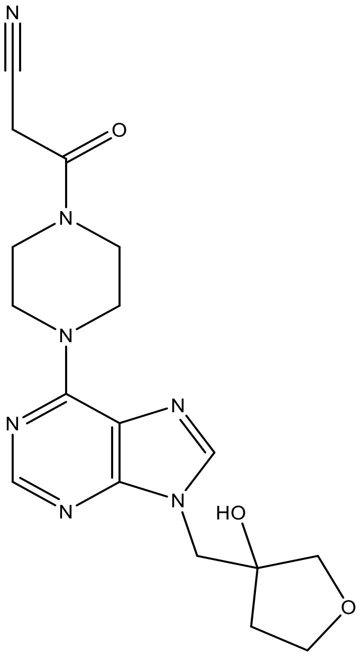
118	OC1(CN2C=NC=3C(=NC=NC23)N4CCN(CC4)C(=O)CC#N)CCOC1
	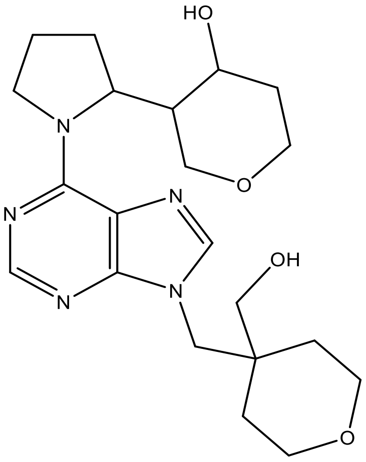
119	OCC1(CN2C=NC=3C(=NC=NC23)N4CCCC4C5COCCC5O)CCOCC1
	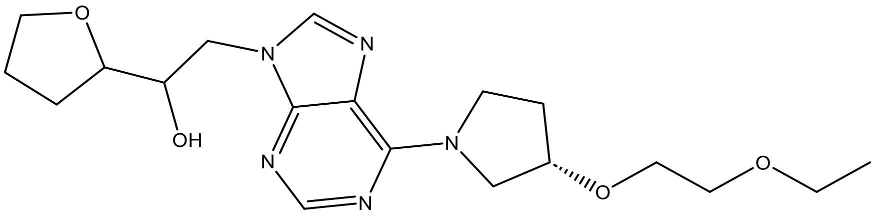
120	CCOCCO[C@H]1CCN(C1)C=2N=CN=C3N(CC(O)C4CCCO4)C=NC23

121	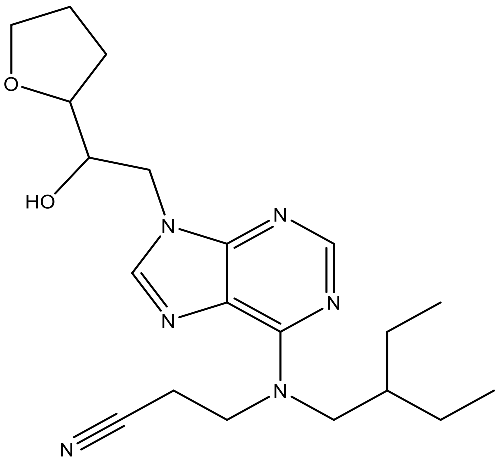<br>CCC(CC)CN(CCC#N)C=1N=CN=C2N(CC(O)C3CCCO3)C=NC12
122	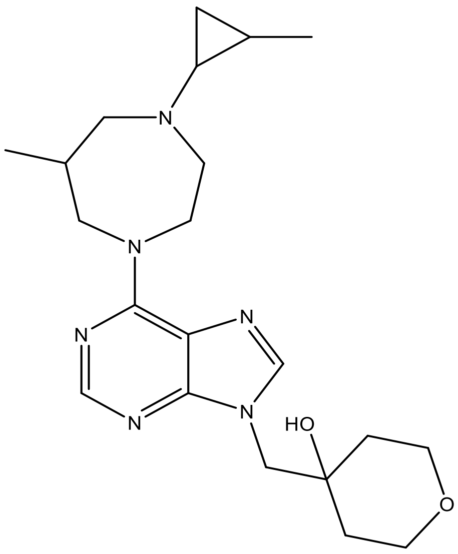<br>CC1CC1N2CCN(CC(C)C2)C=3N=CN=C4N(CC5(O)CCOCC5)C=NC34
123	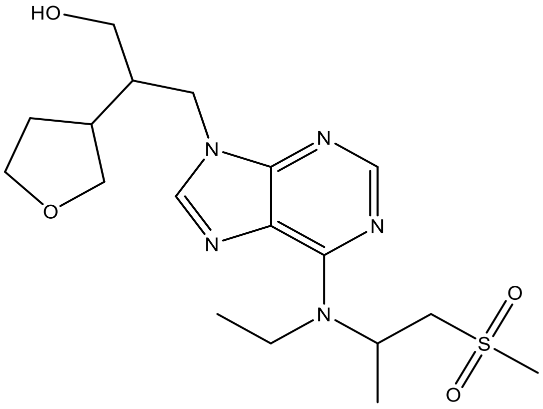<br>CCN(C(C)CS(=O)(=O)C)C=1N=CN=C2N(CC(CO)C3CCOC3)C=NC12
124	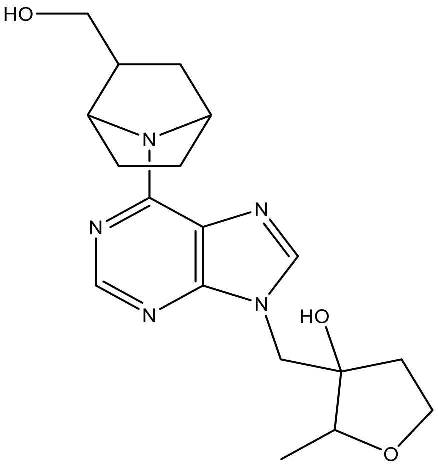<br>CC1OCCC1(O)CN2C=NC=3C(=NC=NC23)N4C5CCC4C(CO)C5

125	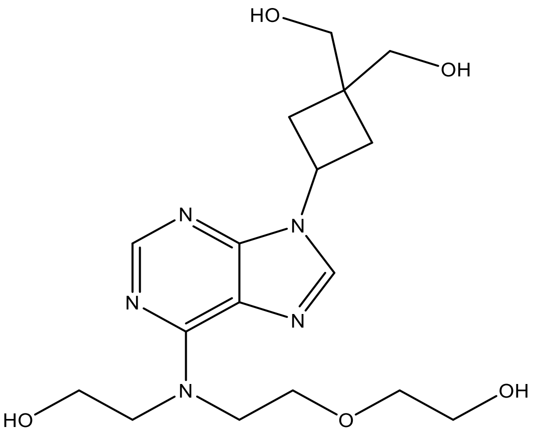<br>OCCOCCN(CCO)C=1N=CN=C2N(C=NC12)C3CC(CO)(CO)C3
126	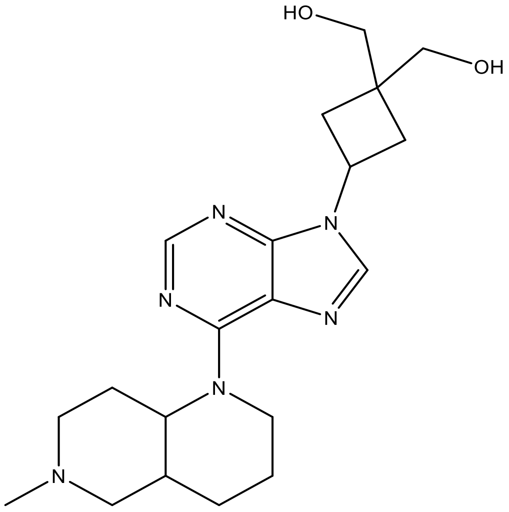<br>CN1CCC2C(CCCN2C=3N=CN=C4N(C=NC34)C5CC(CO)(CO)C5)C1
127	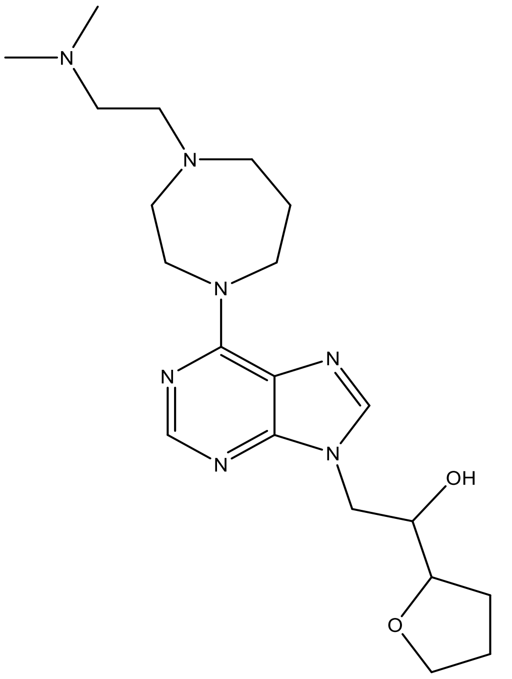<br>CN(C)CCN1CCCN(CC1)C=2N=CN=C3N(CC(O)C4CCCO4)C=NC23
128	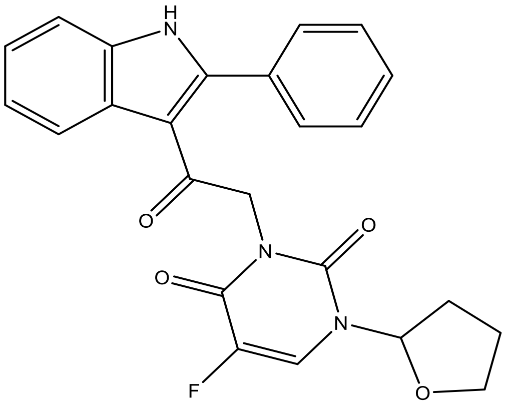<br>FC1=CN(C2CCCO2)C(=O)N(CC(=O)C3=C(NC=4C=CC=CC34)C=5C=CC=CC5)C1=O

129	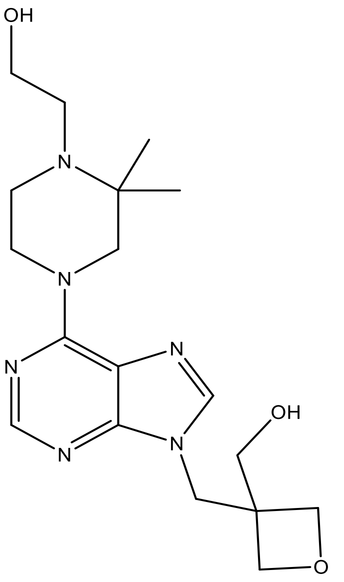 CC1(C)CN(CCN1CCO)C=2N=CN=C3N(CC4(CO)COC4)C=NC23
130	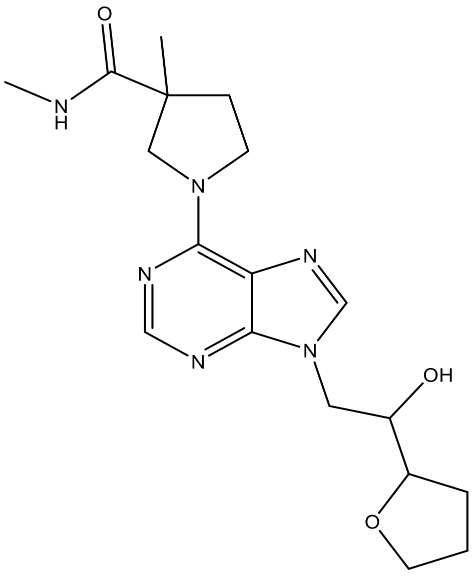 CNC(=O)C1(C)CCN(C1)C=2N=CN=C3N(CC(O)C4CCCO4)C=NC23
131	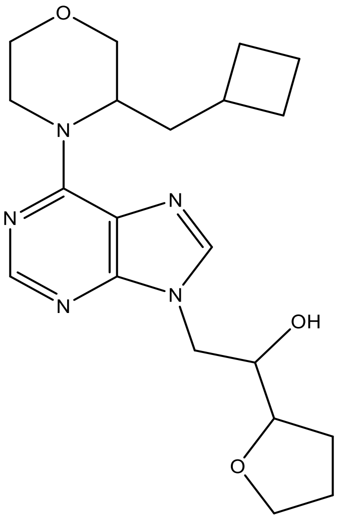 OC(CN1C=NC=2C(=NC=NC12)N3CCOCC3CC4CCC4)C5CCCO5
132	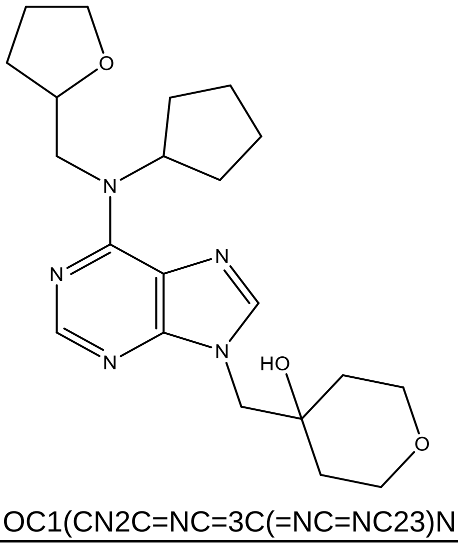 OC1(CN2C=NC=3C(=NC=NC23)N(CC4CCCO4)C5CCCC5)CCOCC1

133	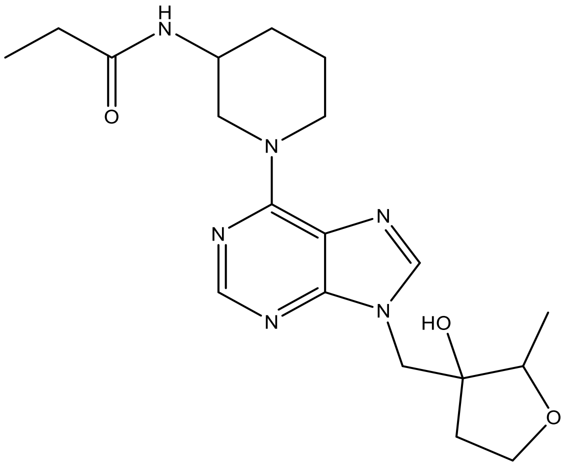<br>CCC(=O)NC1CCCN(C1)C=2N=CN=C3N(CC4(O)CCOC4C)C=NC23
134	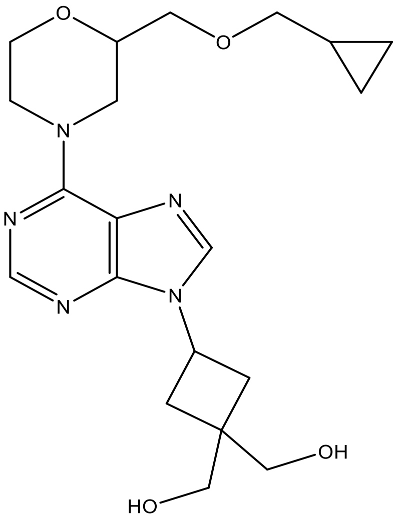<br>OCC1(CO)CC(C1)N2C=NC=3C(=NC=NC23)N4CCOC(COCC5CC5)C4
135	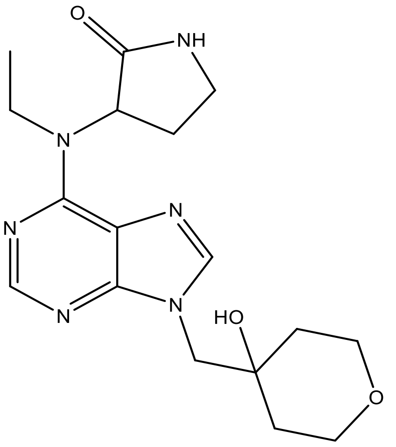<br>CCN(C1CCNC1=O)C=2N=CN=C3N(CC4(O)CCOCC4)C=NC23
136	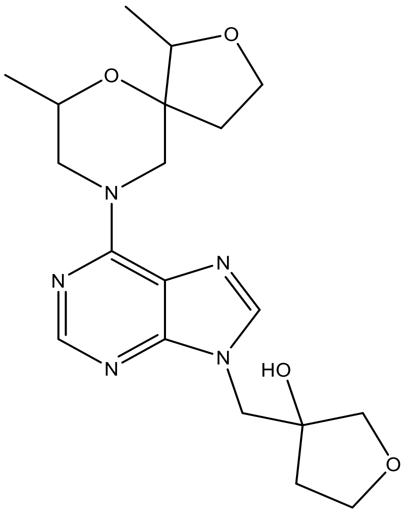<br>CC1OCCC21CN(CC(C)O2)C=3N=CN=C4N(CC5(O)CCOC5)C=NC34

137	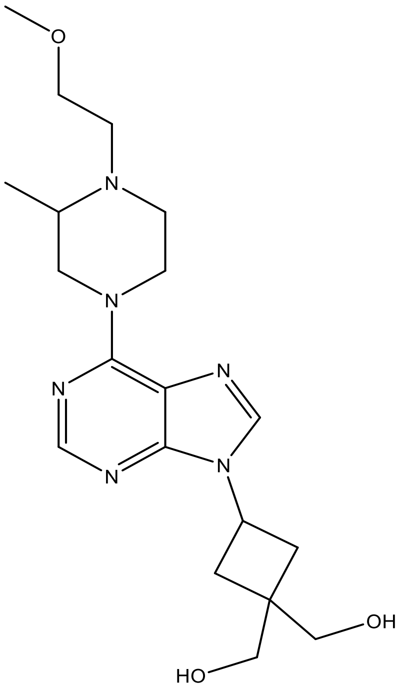 COCCN1CCN(CC1C)C=2N=CN=C3N(C=NC23)C4CC(CO)(CO)C4
138	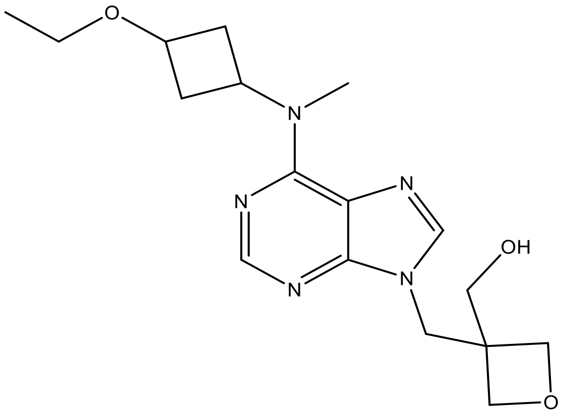 CCOC1CC(C1)N(C)C=2N=CN=C3N(CC4(CO)COC4)C=NC23
139	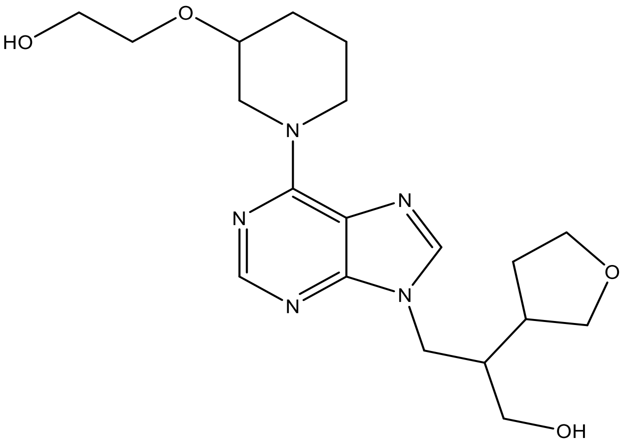 OCCOC1CCCN(C1)C=2N=CN=C3N(CC(CO)C4CCOC4)C=NC23
140	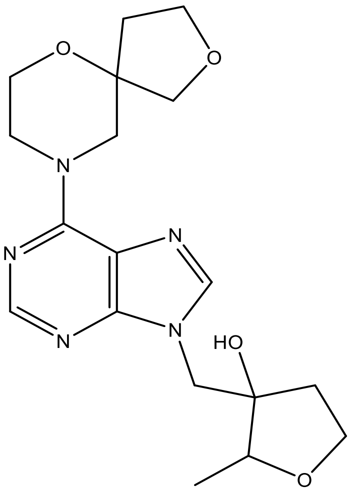 CC1OCCC1(O)CN2C=NC=3C(=NC=NC23)N4CCOC5(CCOC5)C4

141	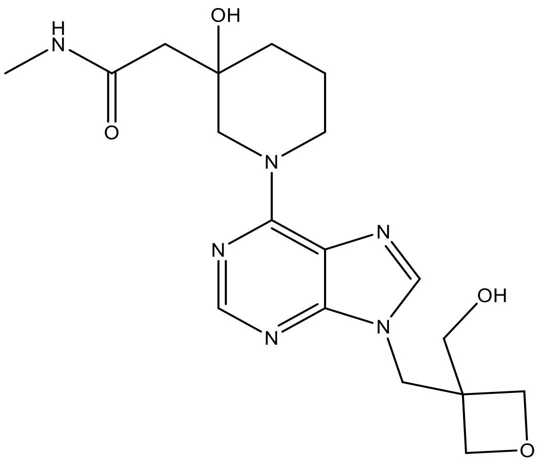<br>CNC(=O)CC1(O)CCCN(C1)C=2N=CN=C3N(CC4(CO)COC4)C=NC23
142	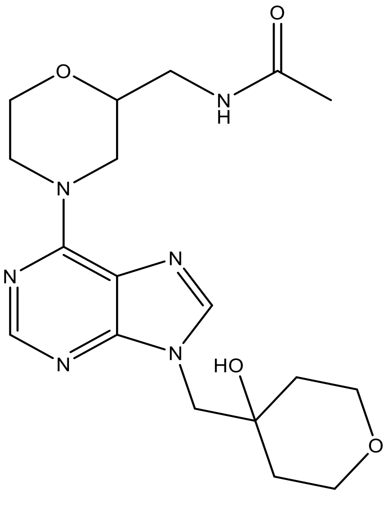<br>CC(=O)NCC1CN(CCO1)C=2N=CN=C3N(CC4(O)CCOCC4)C=NC23
143	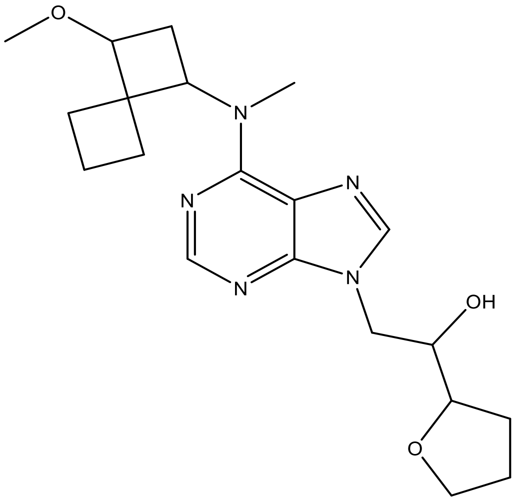<br>COC1CC(N(C)C=2N=CN=C3N(CC(O)C4CCCO4)C=NC23)C51CCC5
144	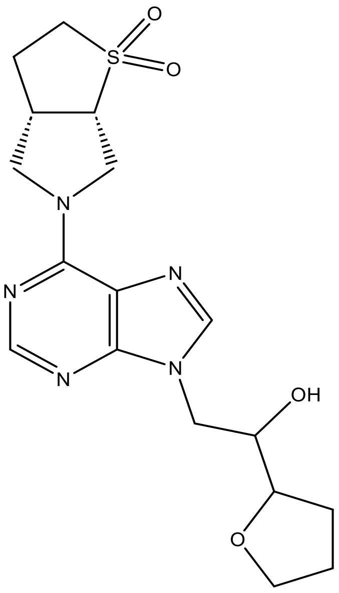<br>OC(CN1C=NC=2C(=NC=NC12)N3C[C@@H]4CCS(=O)(=O)[C@@H]4C3)C5CCCO5

145	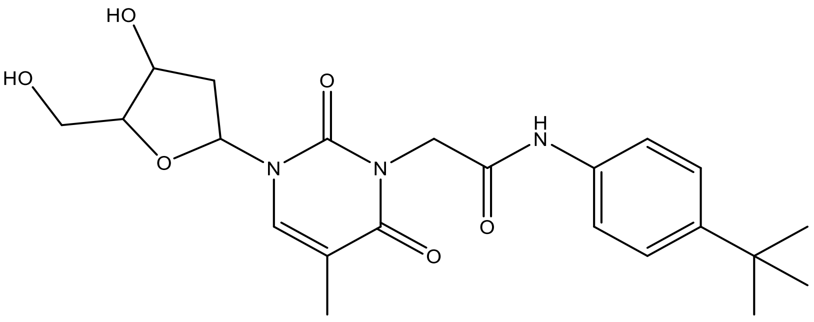 CC1=CN(C2CC(O)C(CO)O2)C(=O)N(CC(=O)NC=3C=CC(=CC3)C(C)(C)C)C1=O
146	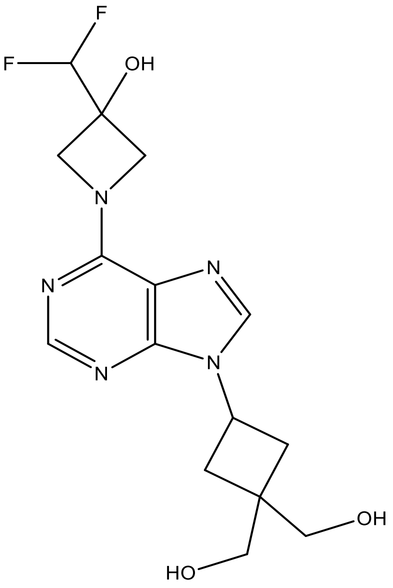 OCC1(CO)CC(C1)N2C=NC=3C(=NC=NC23)N4CC(O)(C4)C(F)F
147	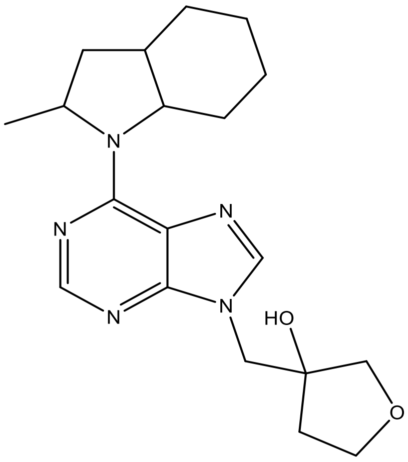 CC1CC2CCCCC2N1C=3N=CN=C4N(CC5(O)CCOC5)C=NC34
148	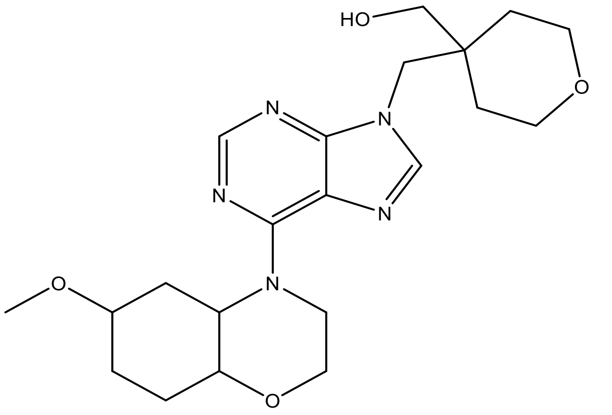 COC1CCC2OCCN(C2C1)C=3N=CN=C4N(CC5(CO)CCOCC5)C=NC34
149	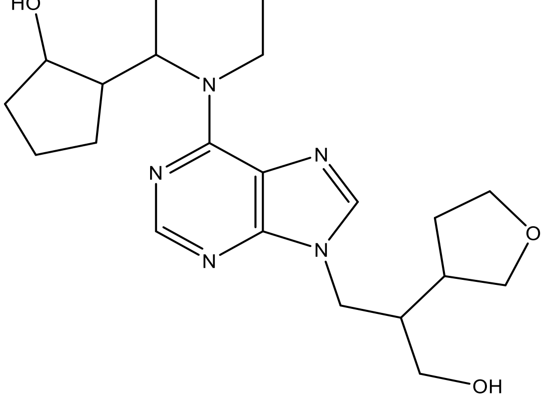 OCC(CN1C=NC=2C(=NC=NC12)N3CCCCC3C4CCCC4O)C5CCOC5

150	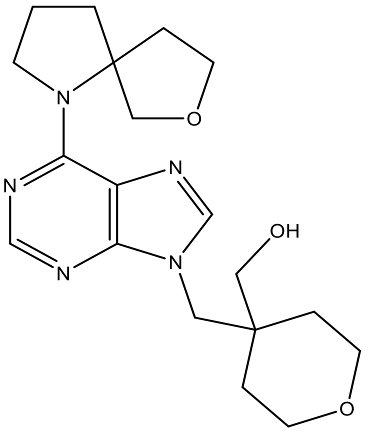<br>OCC1(CN2C=NC=3C(=NC=NC23)N4CCCC54CCOC5)CCOCC1
151	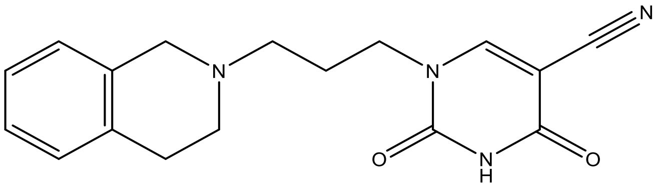<br>O=C1NC(=O)N(CCCN2CCC=3C=CC=CC3C2)C=C1C#N
152	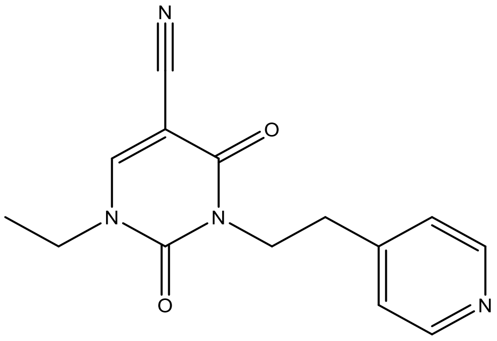<br>CCN1C=C(C#N)C(=O)N(CCC=2C=CN=CC2)C1=O
153	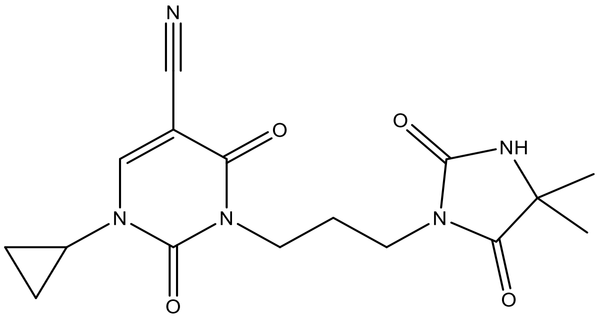<br>CC1(C)NC(=O)N(CCCN2C(=O)C(C#N)=CN(C3CC3)C2=O)C1=O
154	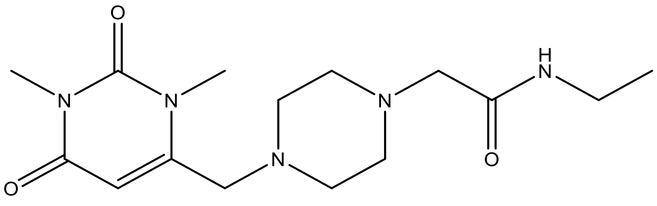<br>CCNC(=O)CN1CCN(CC2=CC(=O)N(C)C(=O)N2C)CC1
155	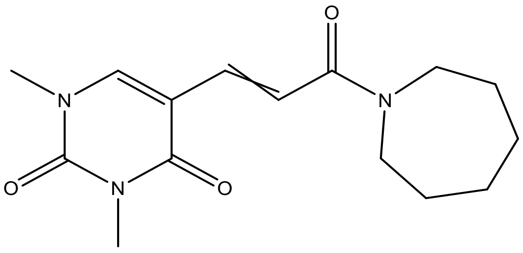<br>CN1C=C(C=CC(=O)N2CCCCCC2)C(=O)N(C)C1=O
156	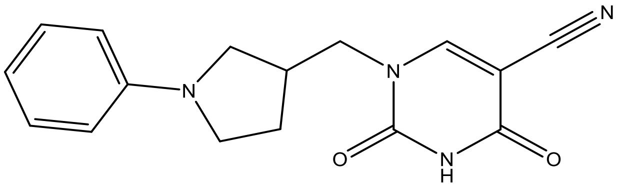<br>O=C1NC(=O)N(CC2CCN(C2)C=3C=CC=CC3)C=C1C#N

157	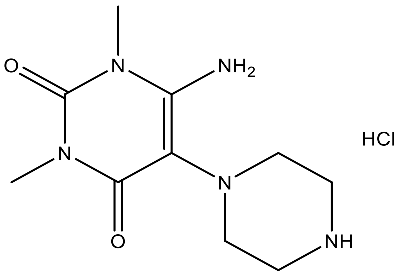<br>Cl.CN1C(N)=C(N2CCNCC2)C(=O)N(C)C1=O
158	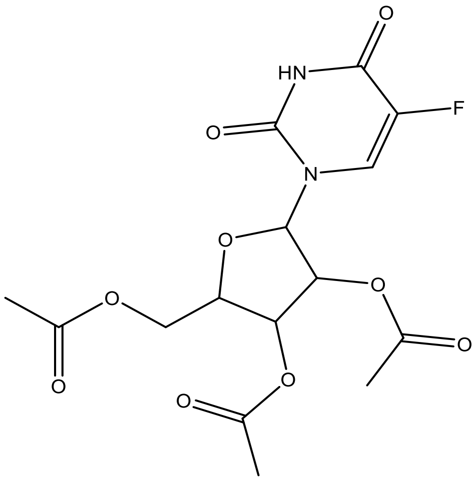<br>CC(=O)OCC1OC(C(OC(=O)C)C1OC(=O)C)N2C=C(F)C(=O)NC2=O
159	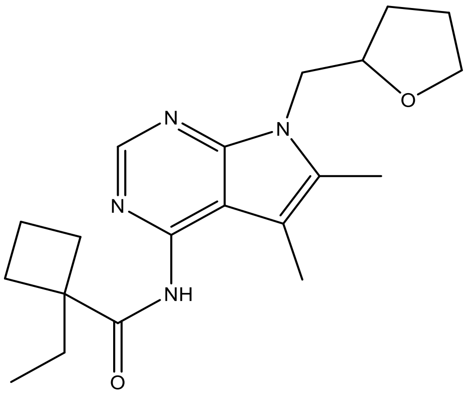<br>CCC1(CCC1)C(=O)NC=2N=CN=C3N(CC4CCCO4)C(C)=C(C)C23
160	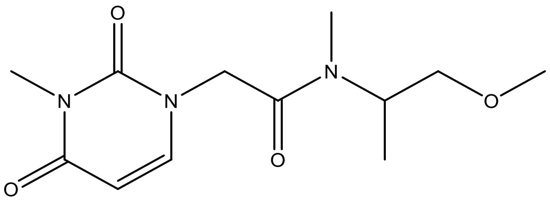<br>COCC(C)N(C)C(=O)CN1C=CC(=O)N(C)C1=O
161	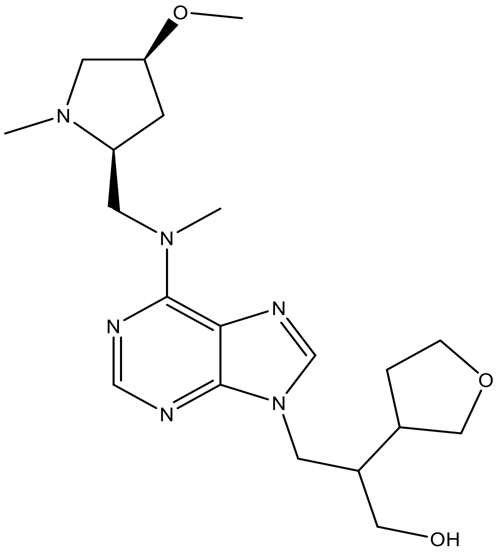<br>CO[C@H]1C[C@@H](CN(C)C=2N=CN=C3N(CC(CO)C4CCOC4)C=NC23)N(C)C1

162	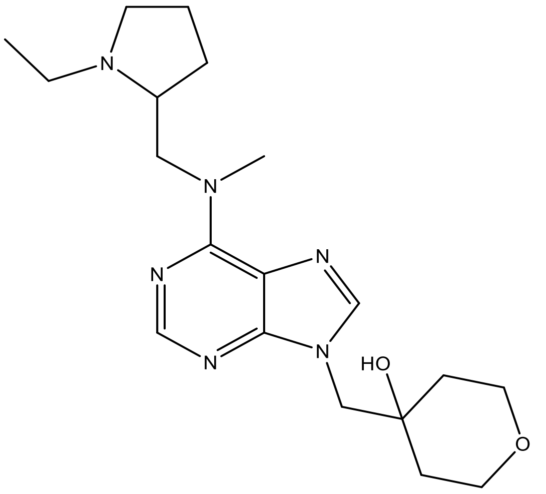<br>CCN1CCCC1CN(C)C=2N=CN=C3N(CC4(O)CCOCC4)C=NC23
163	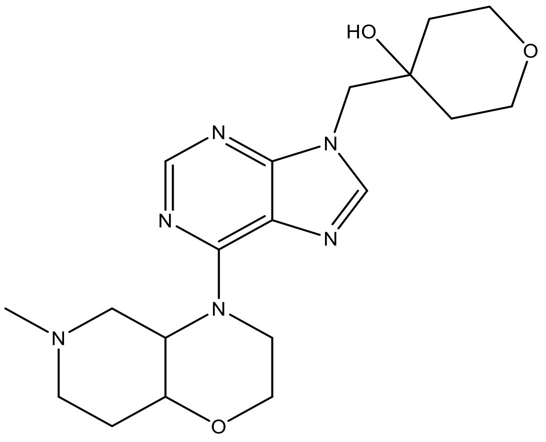<br>CN1CCC2OCCN(C2C1)C=3N=CN=C4N(CC5(O)CCOCC5)C=NC34
164	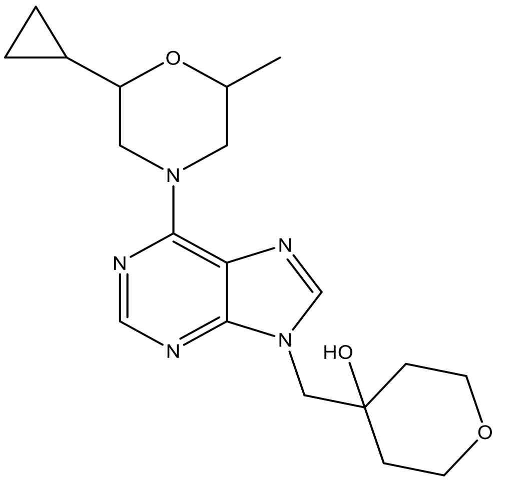<br>CC1CN(CC(O1)C2CC2)C=3N=CN=C4N(CC5(O)CCOCC5)C=NC34
165	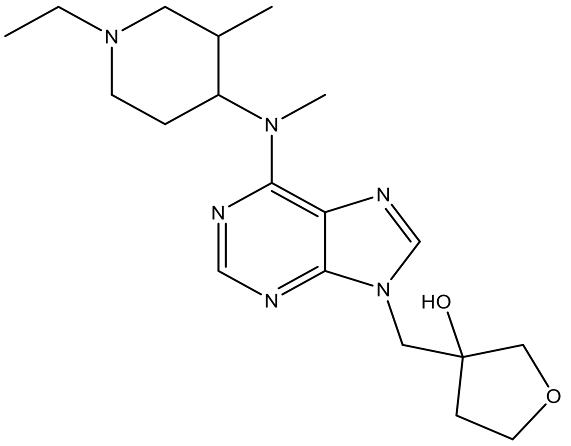<br>CCN1CCC(C(C)C1)N(C)C=2N=CN=C3N(CC4(O)CCOC4)C=NC23

	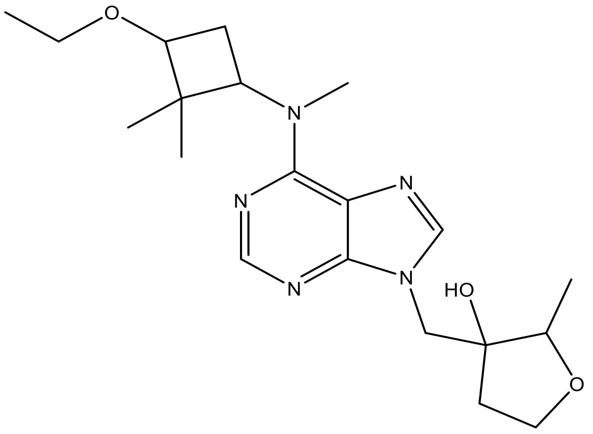
166	CCOC1CC(N(C)C=2N=CN=C3N(CC4(O)CCOC4C)C=NC23)C1(C)C
	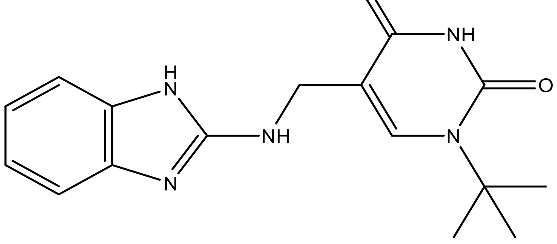
167	CC(C)(C)N1C=C(CNC2=NC=3C=CC=CC3N2)C(=O)NC1=O
	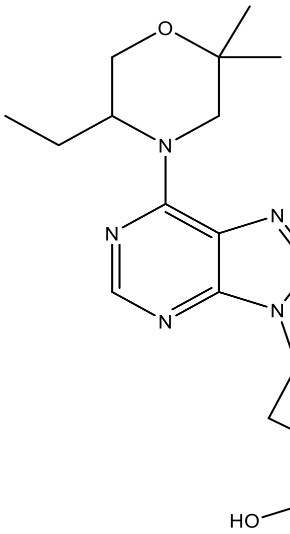
168	CCC1COC(C)(C)CN1C=2N=CN=C3N(C=NC23)C4CC(CO)(CO)C4
	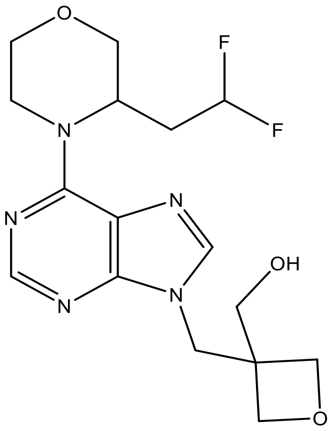
169	OCC1(CN2C=NC=3C(=NC=NC23)N4CCOCC4CC(F)F)COC1
	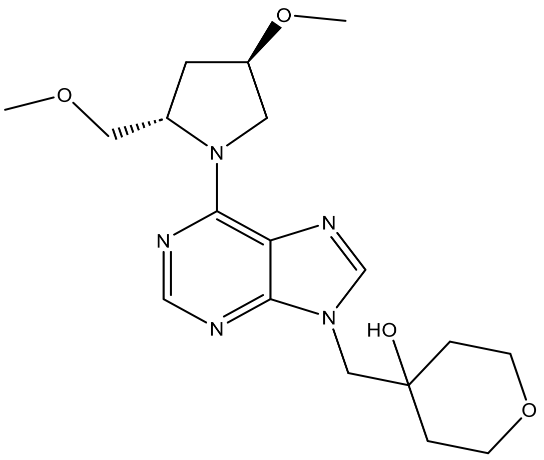
170	COC[C@@H]1C[C@H](CN1C=2N=CN=C3N(CC4(O)CCOCC4)C=NC23)OC

	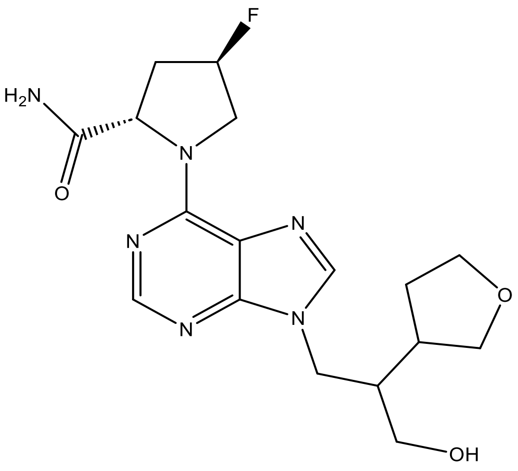
171	NC(=O)[C@@H]1C[C@@H](F)CN1C=2N=CN=C3N(CC(CO)C4CCOC4)C=NC23
	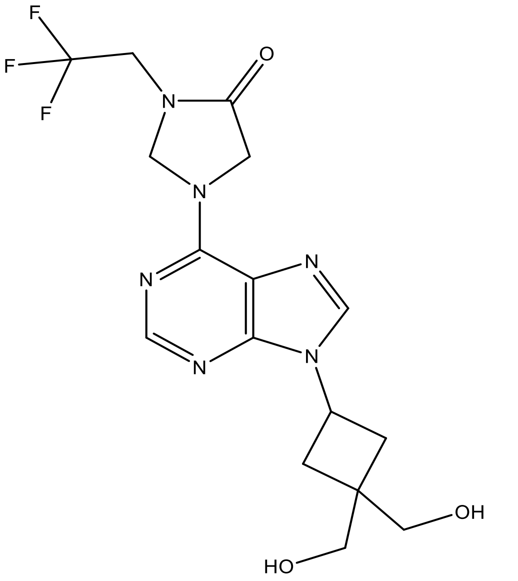
172	OCC1(CO)CC(C1)N2C=NC=3C(=NC=NC23)N4CN(CC(F)(F)F)C(=O)C4
	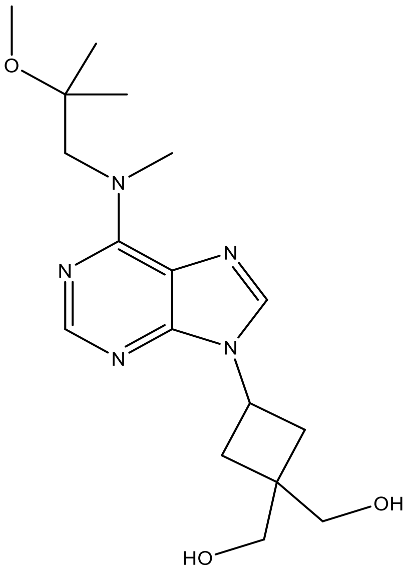
173	COC(C)(C)CN(C)C=1N=CN=C2N(C=NC12)C3CC(CO)(CO)C3
	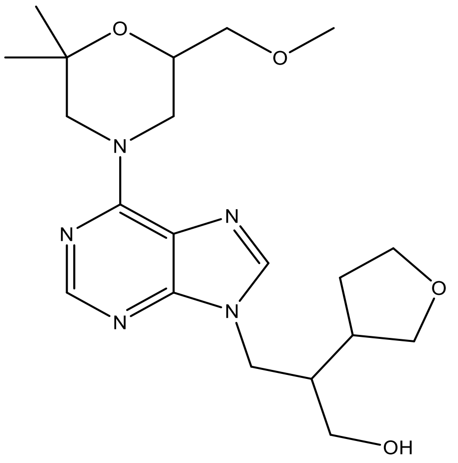
174	COCC1CN(CC(C)(C)O1)C=2N=CN=C3N(CC(CO)C4CCOC4)C=NC23

175	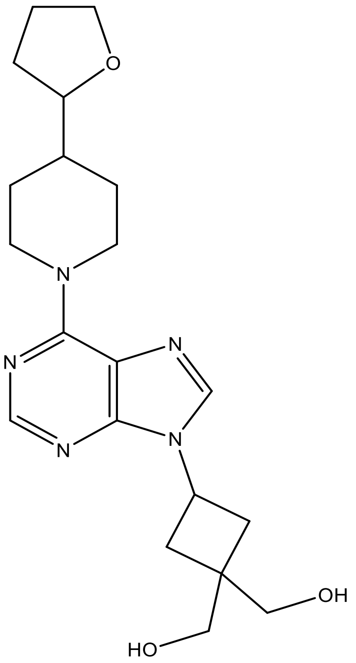 OCC1(CO)CC(C1)N2C=NC=3C(=NC=NC23)N4CCC(CC4)C5CCCO5
176	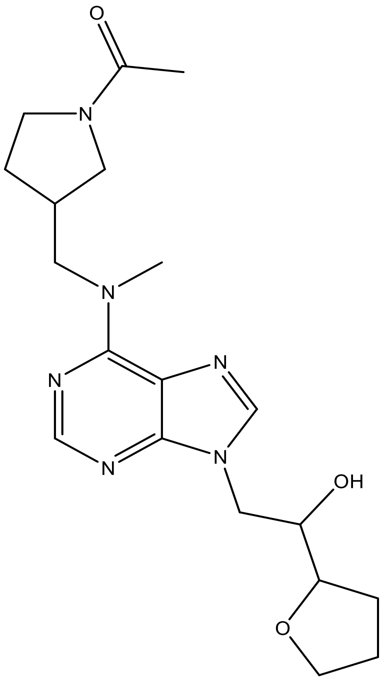 CN(CC1CCN(C1)C(=O)C)C=2N=CN=C3N(CC(O)C4CCCO4)C=NC23
177	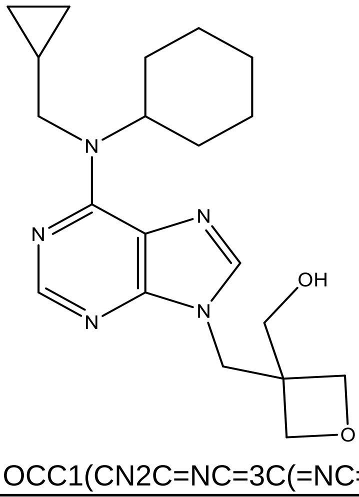 OCC1(CN2C=NC=3C(=NC=NC23)N(CC4CC4)C5CCCCC5)COC1

178	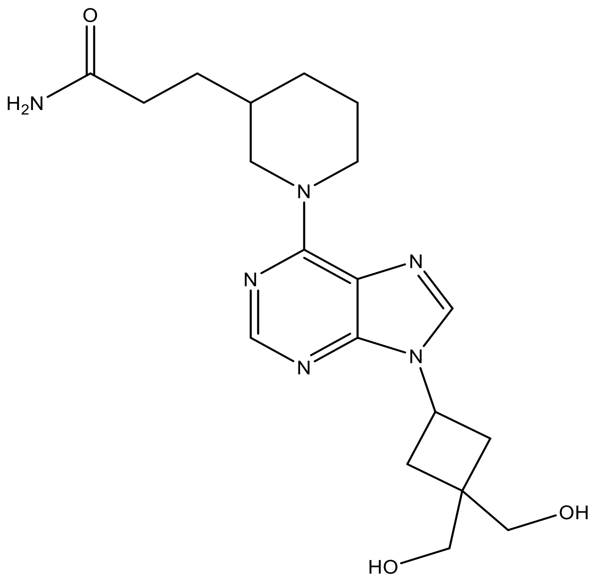 NC(=O)CCC1CCCN(C1)C=2N=CN=C3N(C=NC23)C4CC(CO)(CO)C4
179	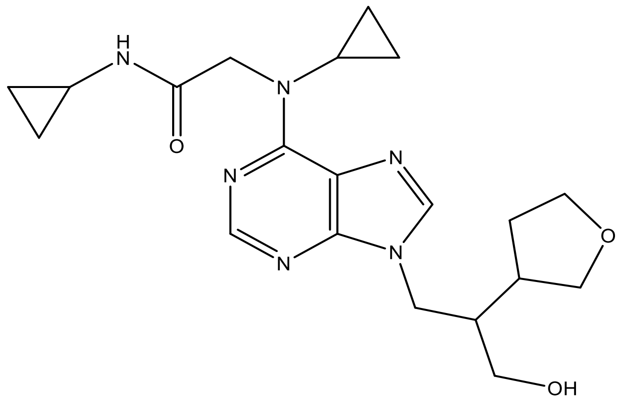 OCC(CN1C=NC=2C(=NC=NC12)N(CC(=O)NC3CC3)C4CC4)C5CCOC5
180	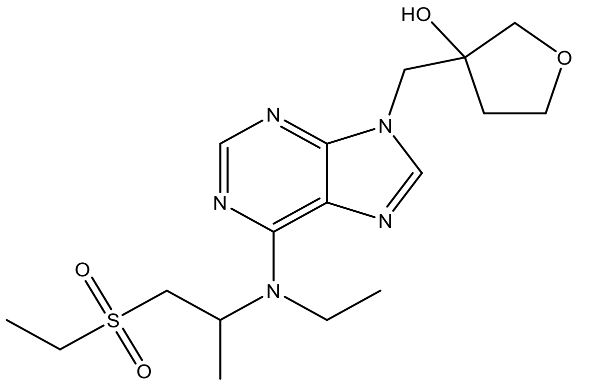 CCN(C(C)CS(=O)(=O)CC)C=1N=CN=C2N(CC3(O)CCOC3)C=NC12
181	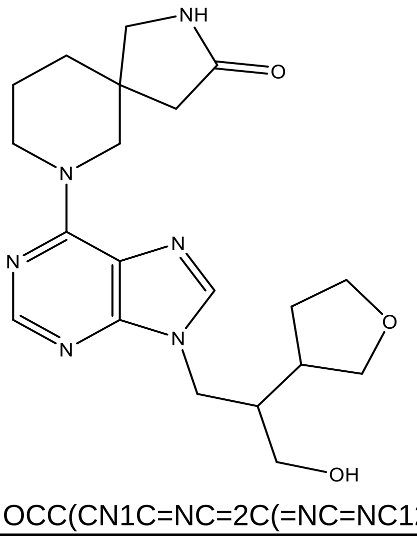 OCC(CN1C=NC=2C(=NC=NC12)N3CCCC4(CNC(=O)C4)C3)C5CCOC5
182	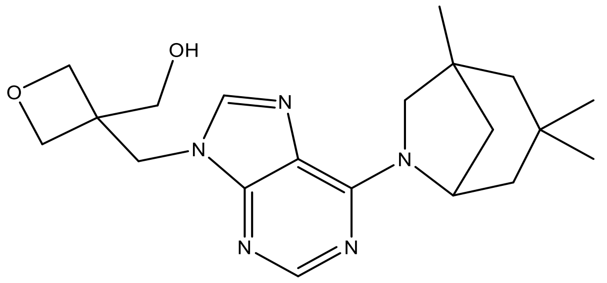 CC1(CC2CC(C)(C)C1)CN2C=3N=CN=C4N(CC5(CO)COC5)C=NC34

183	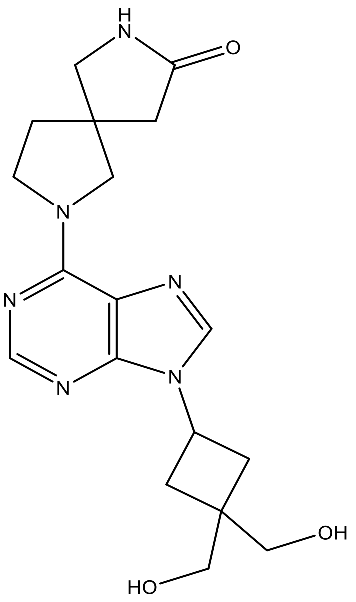 OCC1(CO)CC(C1)N2C=NC=3C(=NC=NC23)N4CCC5(CNC(=O)C5)C4
184	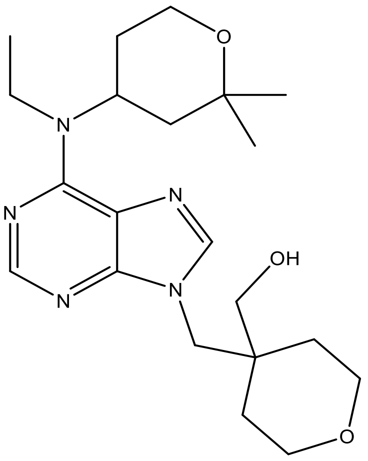 CCN(C1CCOC(C)(C)C1)C=2N=CN=C3N(CC4(CO)CCOCC4)C=NC23
185	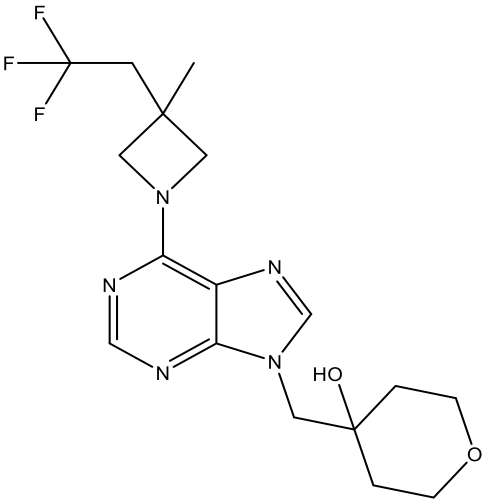 CC1(CC(F)(F)F)CN(C1)C=2N=CN=C3N(CC4(O)CCOCC4)C=NC23
186	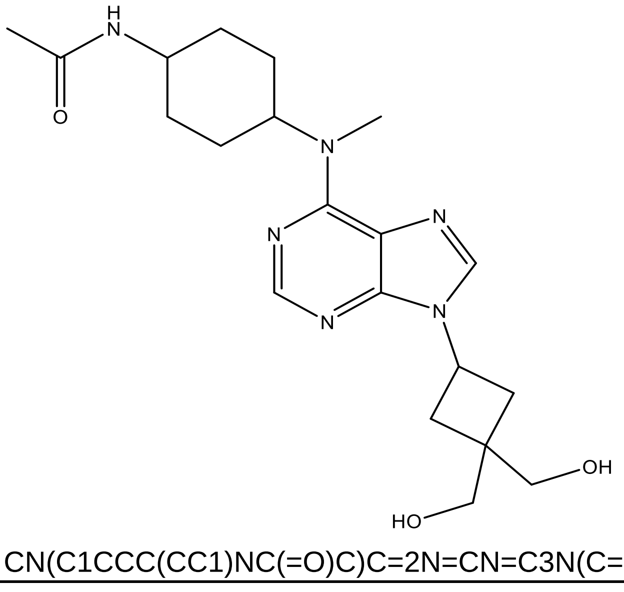 CN(C1CCC(CC1)NC(=O)C)C=2N=CN=C3N(C=NC23)C4CC(CO)(CO)C4

187	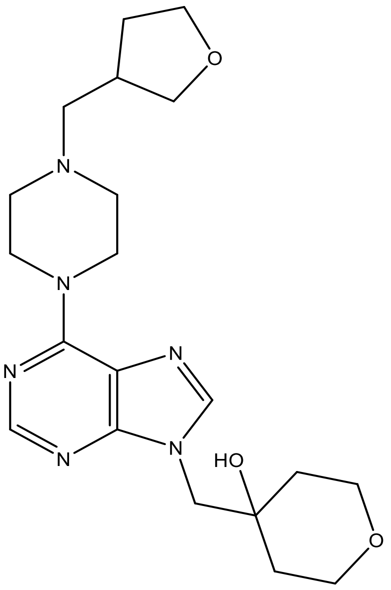 OC1(CN2C=NC=3C(=NC=NC23)N4CCN(CC5CCOC5)CC4)CCOCC1
188	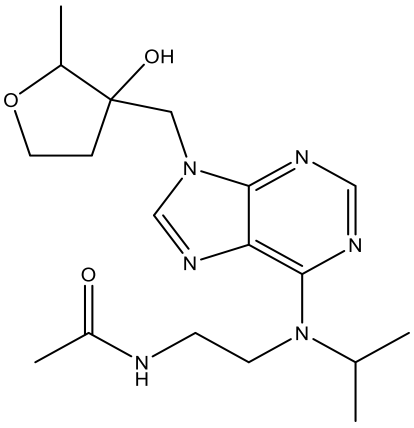 CC(C)N(CCNC(=O)C)C=1N=CN=C2N(CC3(O)CCOC3C)C=NC12
189	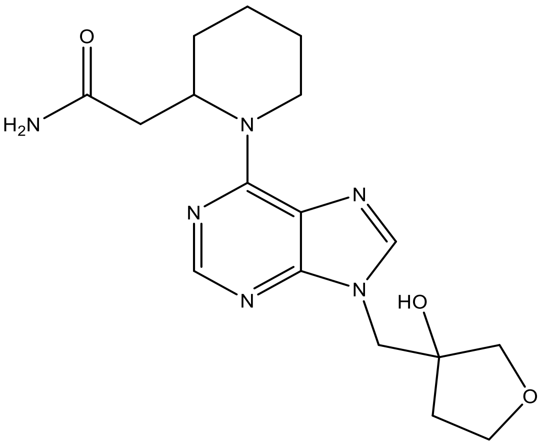 NC(=O)CC1CCCCN1C=2N=CN=C3N(CC4(O)CCOC4)C=NC23
190	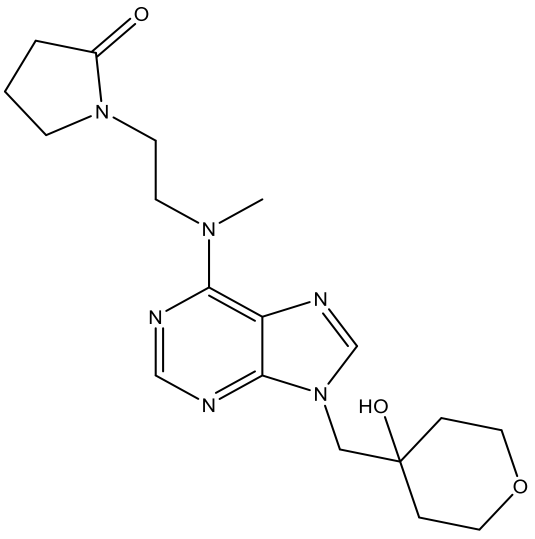 CN(CCN1CCCC1=O)C=2N=CN=C3N(CC4(O)CCOCC4)C=NC23

191	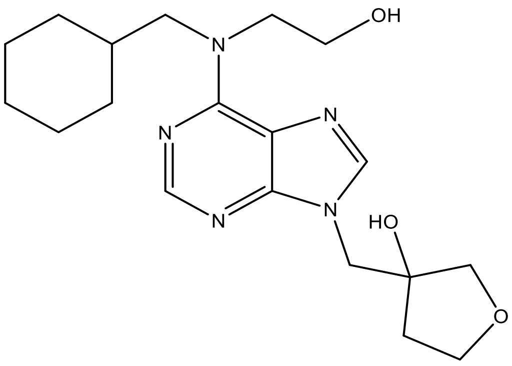<br>OCCN(CC1CCCCC1)C=2N=CN=C3N(CC4(O)CCOC4)C=NC23
192	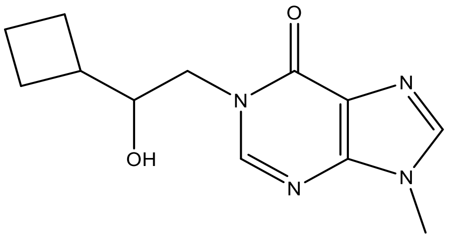<br>CN1C=NC=2C(=O)N(CC(O)C3CCC3)C=NC12
193	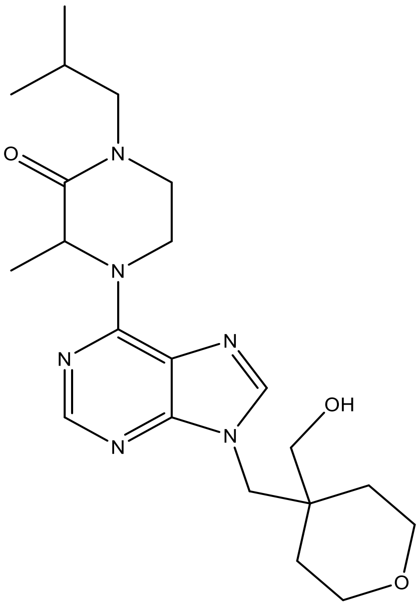<br>CC(C)CN1CCN(C(C)C1=O)C=2N=CN=C3N(CC4(CO)CCOCC4)C=NC23
194	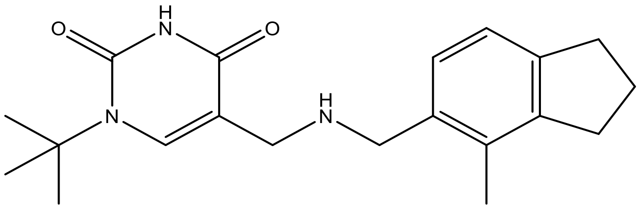<br>CC=1C=2CCCC2C=CC1CNCC3=CN(C(=O)NC3=O)C(C)(C)C
195	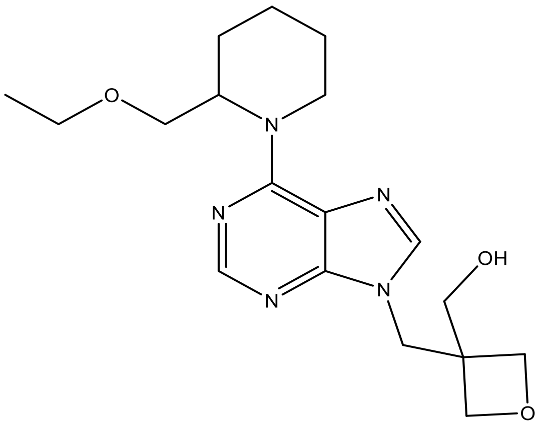<br>CCOCC1CCCCN1C=2N=CN=C3N(CC4(CO)COC4)C=NC23

196	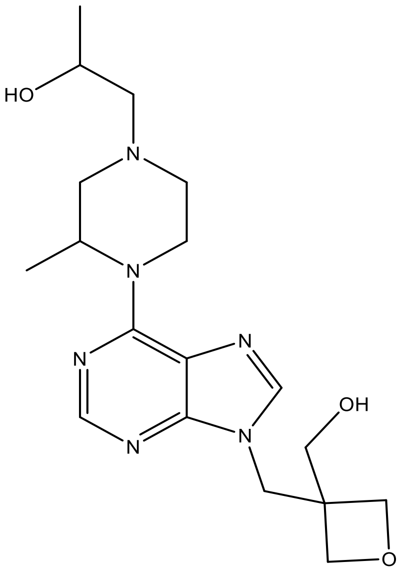<br>CC(O)CN1CCN(C(C)C1)C=2N=CN=C3N(CC4(CO)COC4)C=NC23
197	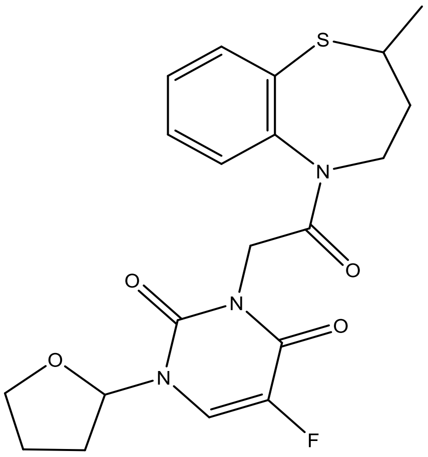<br>CC1CCN(C(=O)CN2C(=O)C(F)=CN(C3CCCO3)C2=O)C=4C=CC=CC4S1
198	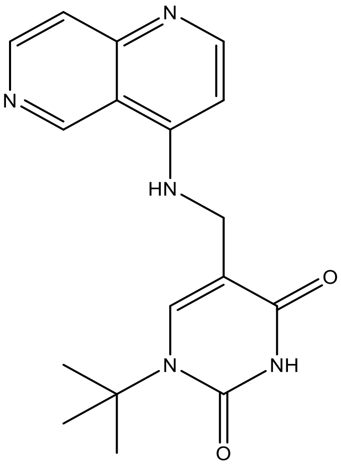<br>CC(C)(C)N1C=C(CNC=2C=CN=C3C=CN=CC23)C(=O)NC1=O
199	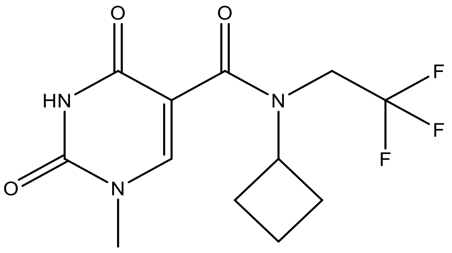<br>CN1C=C(C(=O)N(CC(F)(F)F)C2CCC2)C(=O)NC1=O
200	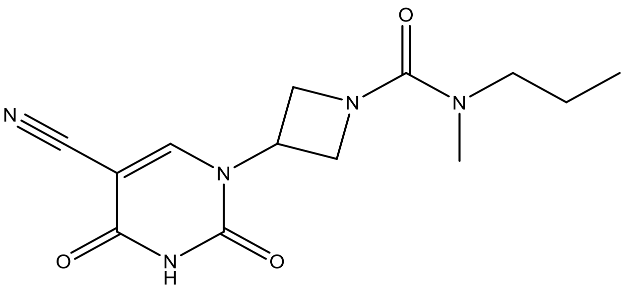<br>CCCN(C)C(=O)N1CC(C1)N2C=C(C#N)C(=O)NC2=O

201	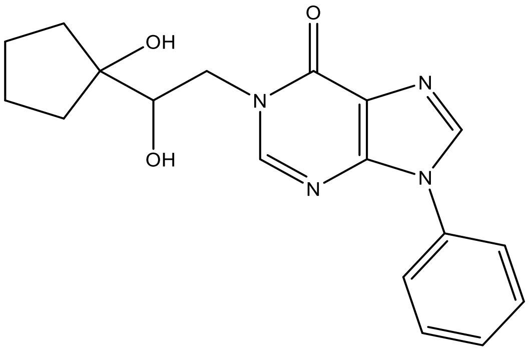<br>OC(CN1C=NC=2N(C=NC2C1=O)C=3C=CC=CC3)C4(O)CCCC4
202	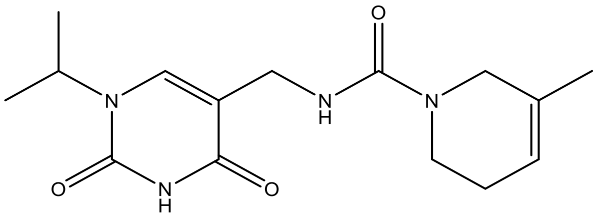<br>CC(C)N1C=C(CNC(=O)N2CCC=C(C)C2)C(=O)NC1=O
203	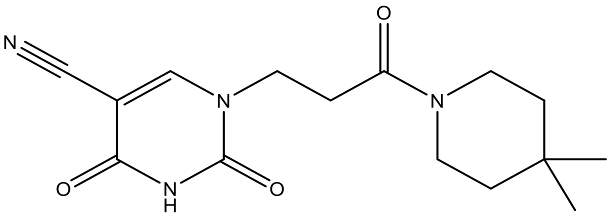<br>CC1(C)CCN(CC1)C(=O)CCN2C=C(C#N)C(=O)NC2=O
204	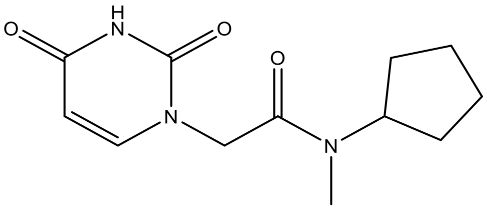<br>CN(C1CCCC1)C(=O)CN2C=CC(=O)NC2=O
205	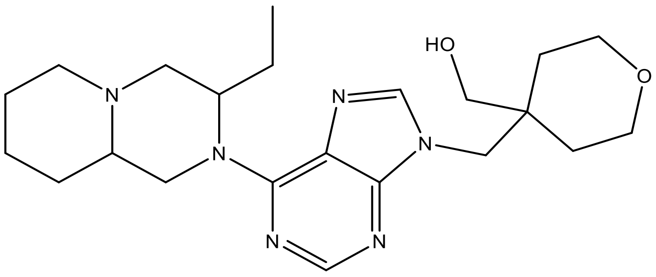<br>CCC1CN2CCCCC2CN1C=3N=CN=C4N(CC5(CO)CCOCC5)C=NC34
206	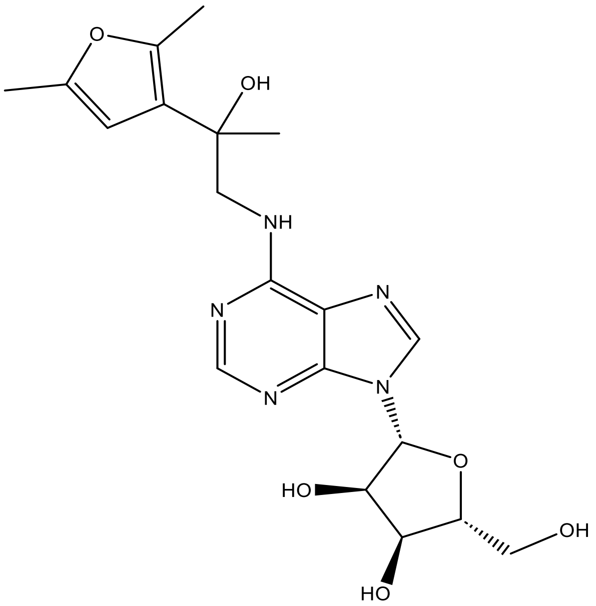<br>CC1=CC(=C(C)O1)C(C)(O)CNC=2N=CN=C3N(C=NC23)[C@@H]4O[C@H](CO)[C@@H](O)[C@H]4O

207	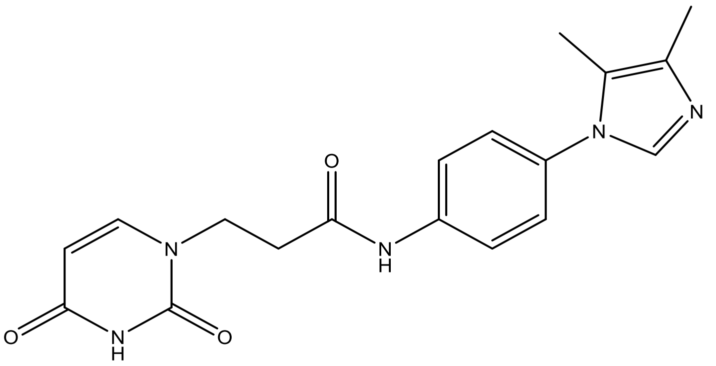 CC=1N=CN(C1C)C=2C=CC(NC(=O)CCN3C=CC(=O)NC3=O)=CC2
208	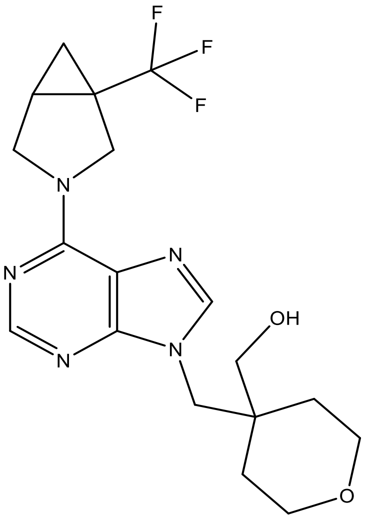 OCC1(CN2C=NC=3C(=NC=NC23)N4CC5CC5(C4)C(F)(F)F)CCOCC1
209	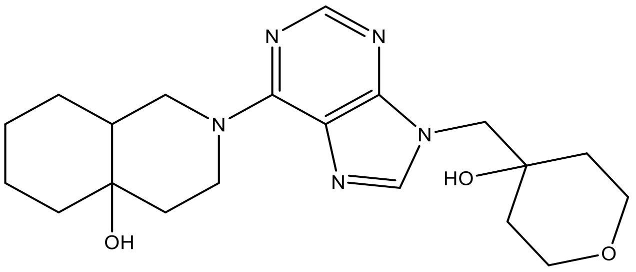 OC1(CN2C=NC=3C(=NC=NC23)N4CCC5(O)CCCCC5C4)CCOCC1
210	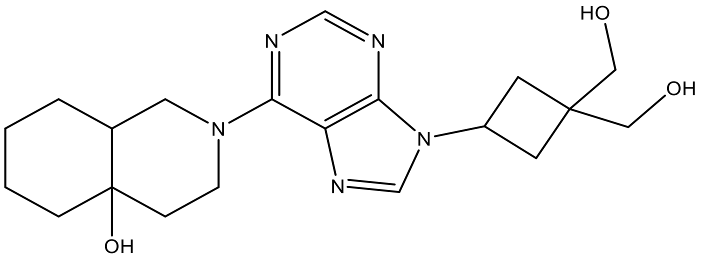 OCC1(CO)CC(C1)N2C=NC=3C(=NC=NC23)N4CCC5(O)CCCCC5C4
211	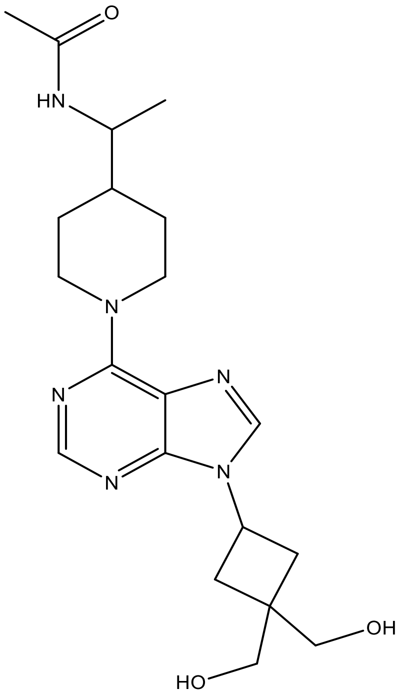 CC(NC(=O)C)C1CCN(CC1)C=2N=CN=C3N(C=NC23)C4CC(CO)(CO)C4

212	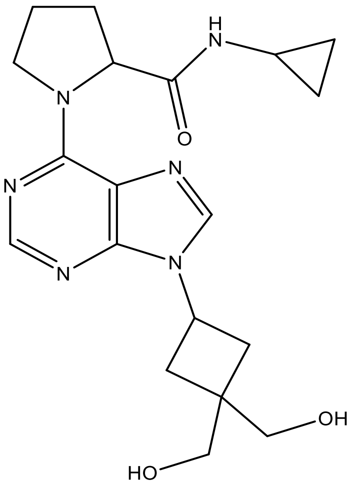<br>OCC1(CO)CC(C1)N2C=NC=3C(=NC=NC23)N4CCCC4C(=O)NC5CC5
213	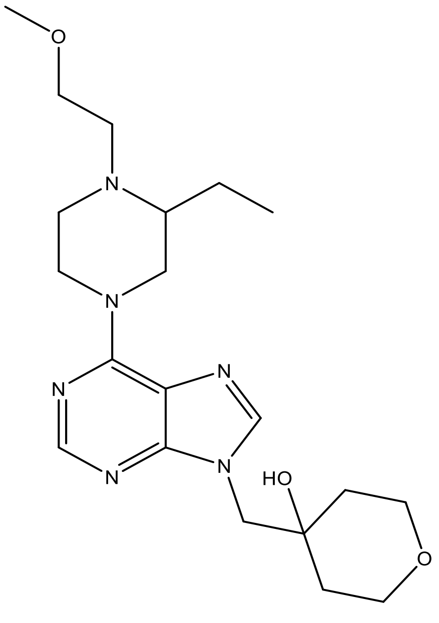<br>CCC1CN(CCN1CCOC)C=2N=CN=C3N(CC4(O)CCOCC4)C=NC23
214	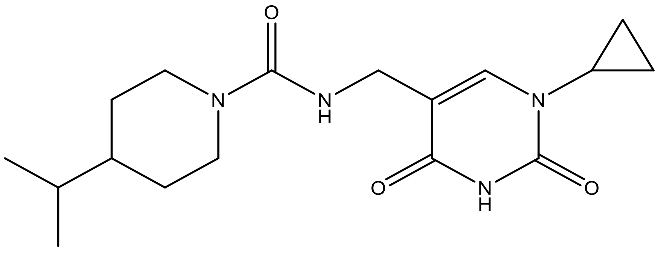<br>CC(C)C1CCN(CC1)C(=O)NCC2=CN(C3CC3)C(=O)NC2=O
215	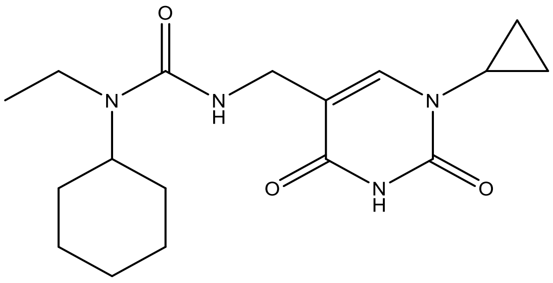<br>CCN(C1CCCCC1)C(=O)NCC2=CN(C3CC3)C(=O)NC2=O
216	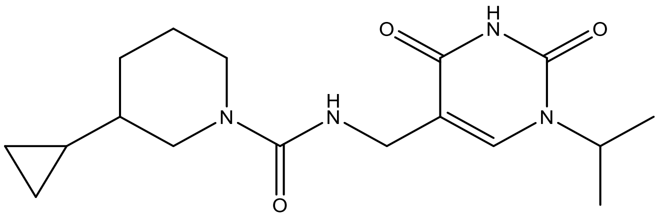<br>CC(C)N1C=C(CNC(=O)N2CCCC(C2)C3CC3)C(=O)NC1=O

217	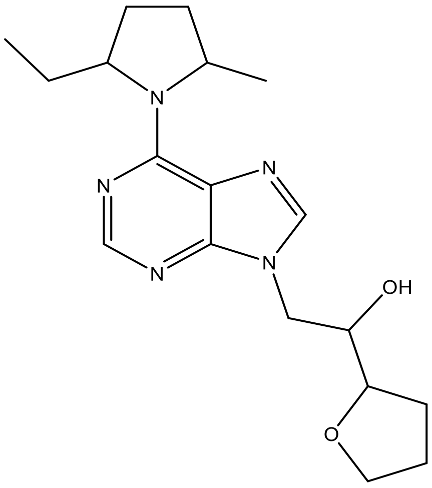 CCC1CCC(C)N1C=2N=CN=C3N(CC(O)C4CCCO4)C=NC23
218	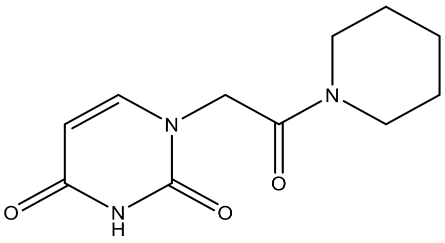 O=C(CN1C=CC(=O)NC1=O)N2CCCCC2
219	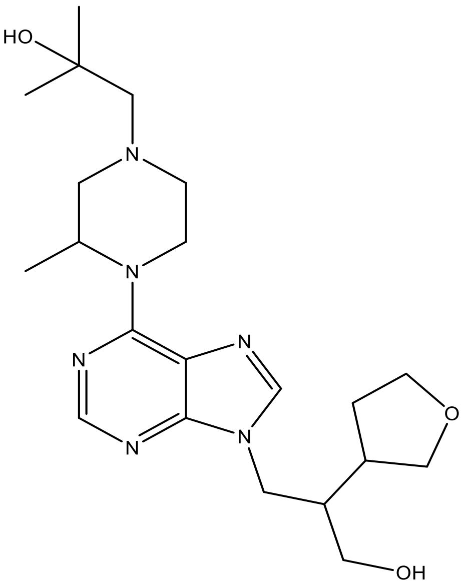 CC1CN(CC(C)(C)O)CCN1C=2N=CN=C3N(CC(CO)C4CCOC4)C=NC23
220	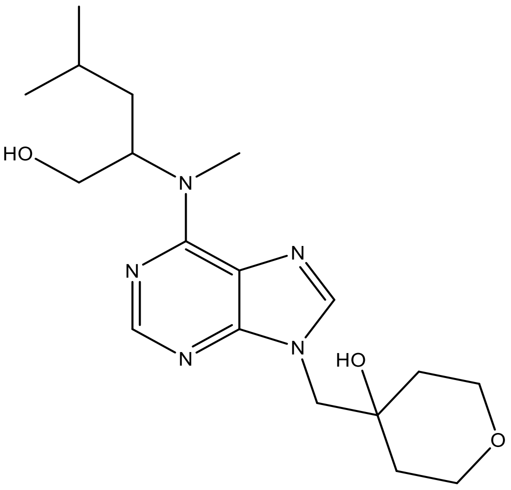 CC(C)CC(CO)N(C)C=1N=CN=C2N(CC3(O)CCOCC3)C=NC12

221	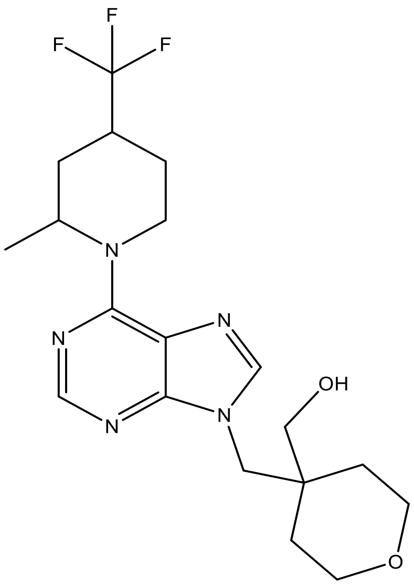 CC1CC(CCN1C=2N=CN=C3N(CC4(CO)CCOCC4)C=NC23)C(F)(F)F
222	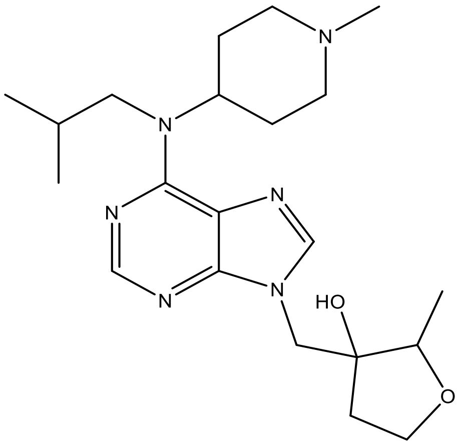 CC(C)CN(C1CCN(C)CC1)C=2N=CN=C3N(CC4(O)CCOC4C)C=NC23
223	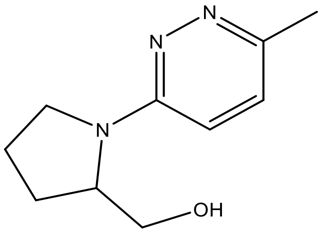 CC=1C=CC(=NN1)N2CCCC2CO
224	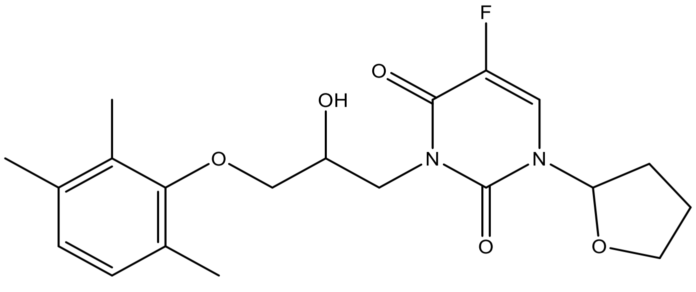 CC=1C=CC(C)=C(OCC(O)CN2C(=O)C(F)=CN(C3CCCO3)C2=O)C1C
225	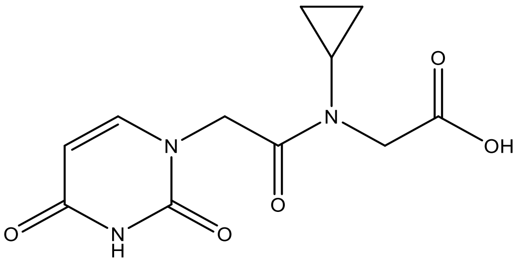 OC(=O)CN(C1CC1)C(=O)CN2C=CC(=O)NC2=O
226	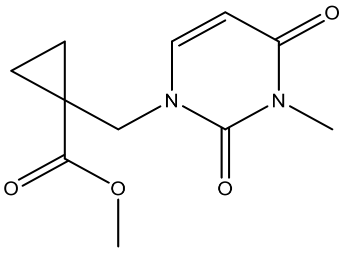 COC(=O)C1(CN2C=CC(=O)N(C)C2=O)CC1

227	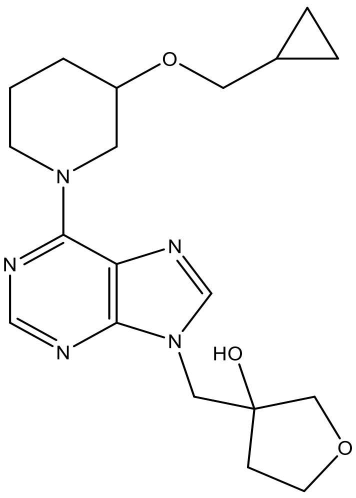<br>OC1(CN2C=NC=3C(=NC=NC23)N4CCCC(C4)OCC5CC5)CCOC1
228	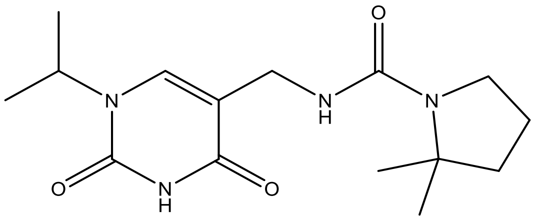<br>CC(C)N1C=C(CNC(=O)N2CCCC2(C)C)C(=O)NC1=O
229	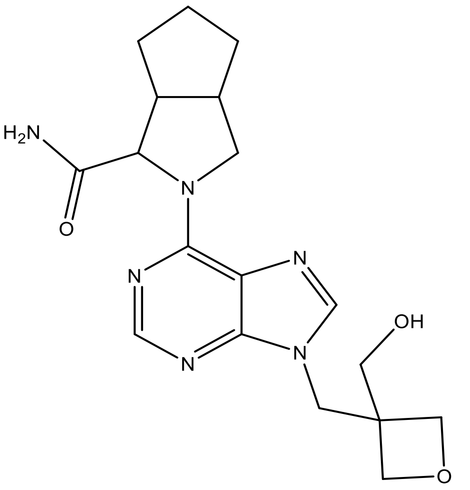<br>NC(=O)C1C2CCCC2CN1C=3N=CN=C4N(CC5(CO)COC5)C=NC34
230	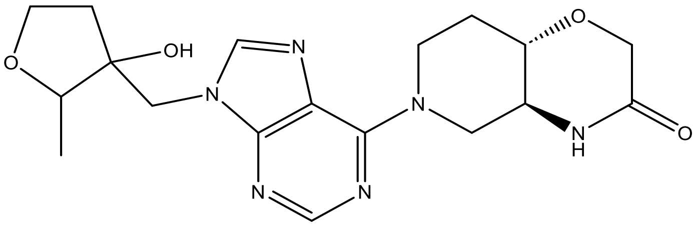<br>CC1OCCC1(O)CN2C=NC=3C(=NC=NC23)N4CC[C@@H]5OCC(=O)N[C@H]5C4
231	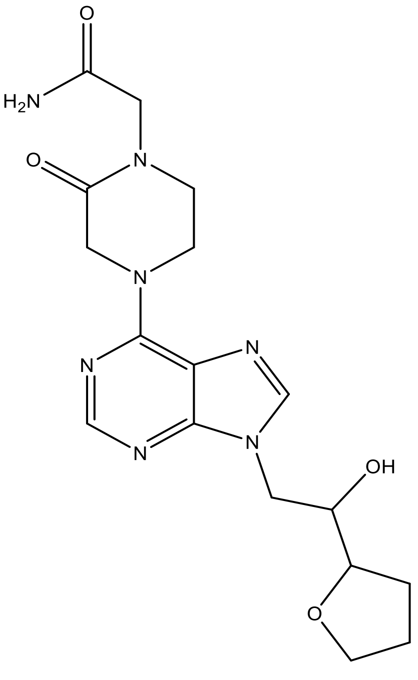<br>NC(=O)CN1CCN(CC1=O)C=2N=CN=C3N(CC(O)C4CCCO4)C=NC23

232	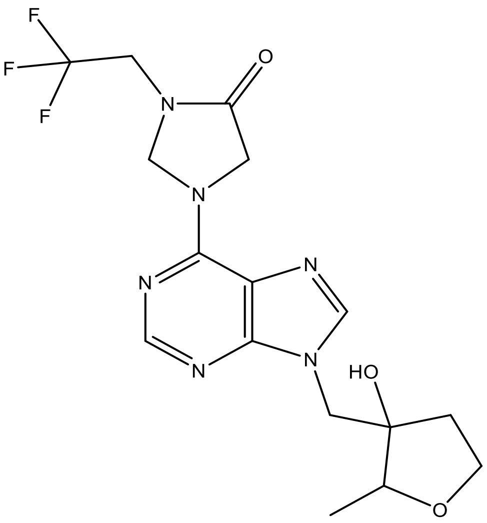 CC1OCCC1(O)CN2C=NC=3C(=NC=NC23)N4CN(CC(F)(F)F)C(=O)C4
233	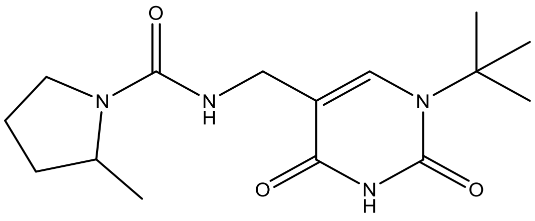 CC1CCCN1C(=O)NCC2=CN(C(=O)NC2=O)C(C)(C)C
234	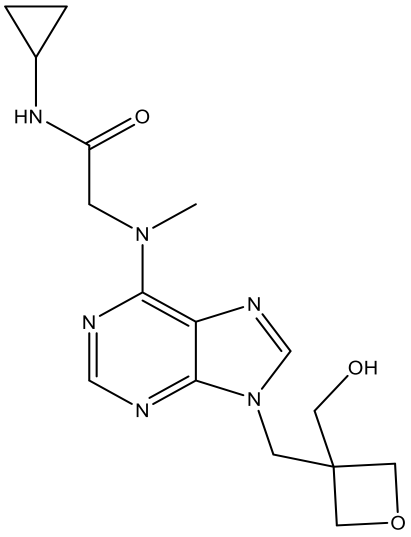 CN(CC(=O)NC1CC1)C=2N=CN=C3N(CC4(CO)COC4)C=NC23
235	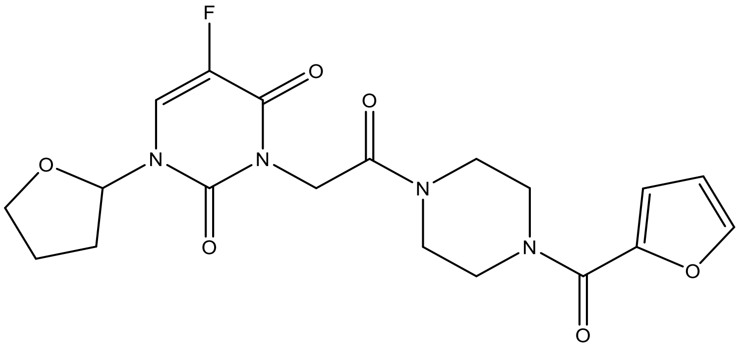 FC1=CN(C2CCCO2)C(=O)N(CC(=O)N3CCN(CC3)C(=O)C4=CC=CO4)C1=O
236	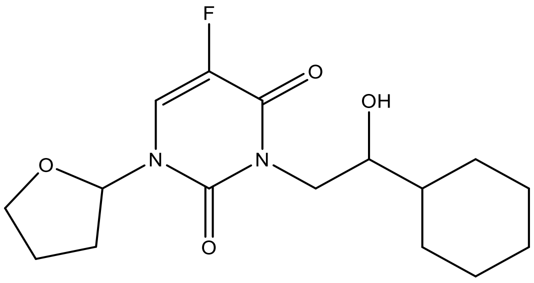 OC(CN1C(=O)C(F)=CN(C2CCCO2)C1=O)C3CCCCC3

237	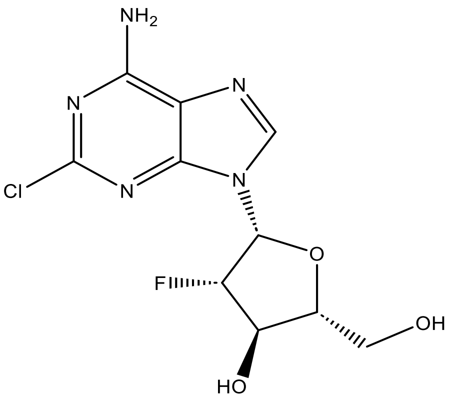<br>NC=1N=C(Cl)N=C2N(C=NC12)[C@@H]3O[C@H](CO)[C@@H](O)[C@@H]3F
238	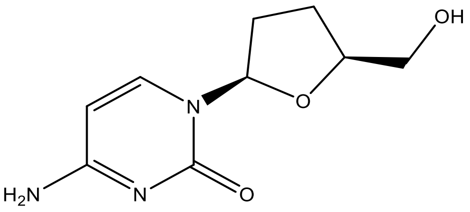<br>NC=1C=CN([C@H]2CC[C@@H](CO)O2)C(=O)N1
239	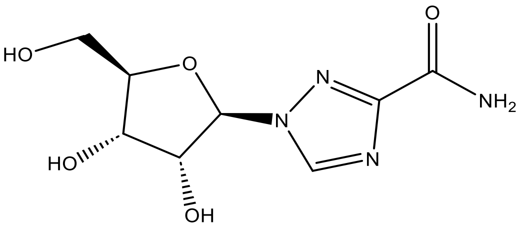<br>NC(=O)C=1N=CN(N1)[C@@H]2O[C@H](CO)[C@@H](O)[C@H]2O
240	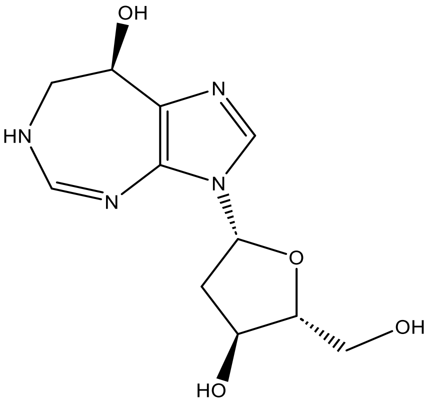<br>OC[C@H]1O[C@H](C[C@@H]1O)N2C=NC=3[C@H](O)CNC=NC32
241	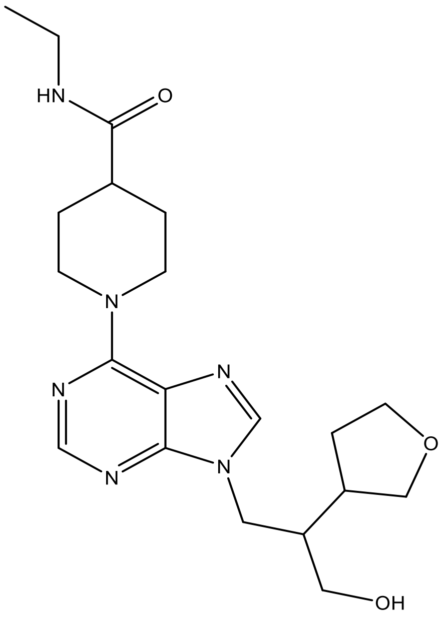<br>CCNC(=O)C1CCN(CC1)C=2N=CN=C3N(CC(CO)C4CCOC4)C=NC23

242	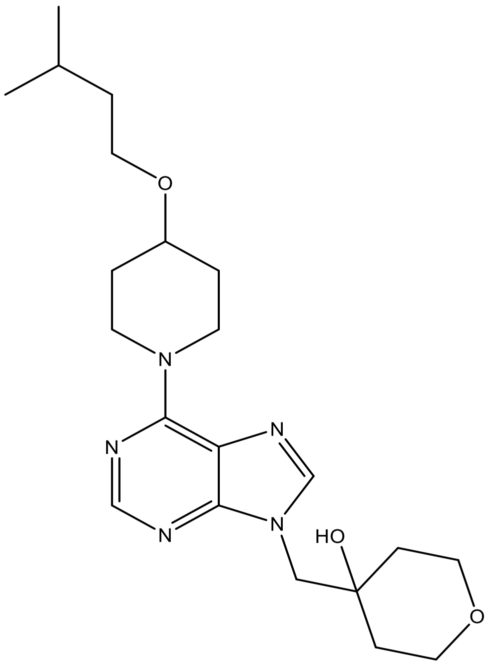 CC(C)CCOC1CCN(CC1)C=2N=CN=C3N(CC4(O)CCOCC4)C=NC23
243	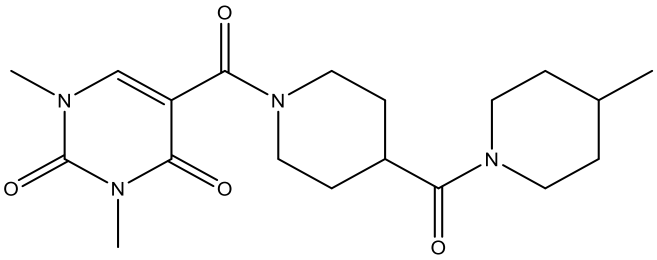 CC1CCN(CC1)C(=O)C2CCN(CC2)C(=O)C3=CN(C)C(=O)N(C)C3=O
244	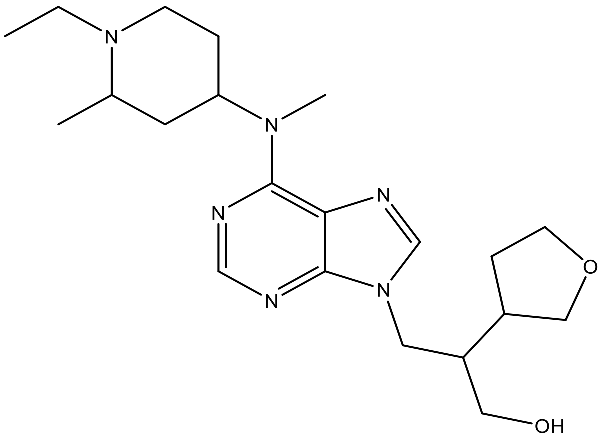 CCN1CCC(CC1C)N(C)C=2N=CN=C3N(CC(CO)C4CCOC4)C=NC23
245	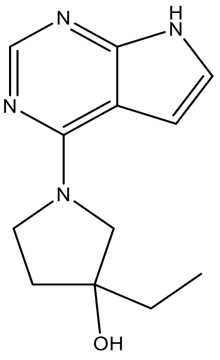 CCC1(O)CCN(C1)C=2N=CN=C3NC=CC23

246	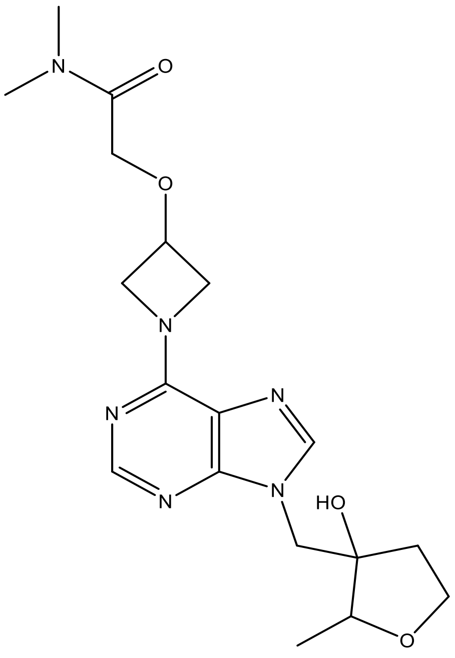<br>CC1OCCC1(O)CN2C=NC=3C(=NC=NC23)N4CC(C4)OCC(=O)N(C)C
247	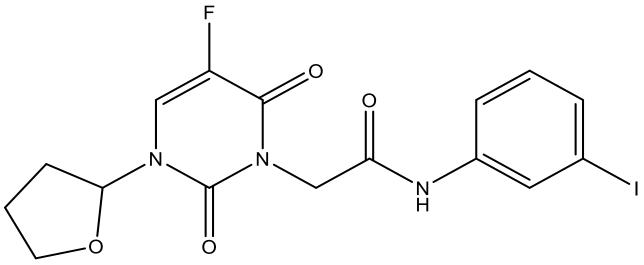<br>FC1=CN(C2CCCO2)C(=O)N(CC(=O)NC=3C=CC=C(I)C3)C1=O
248	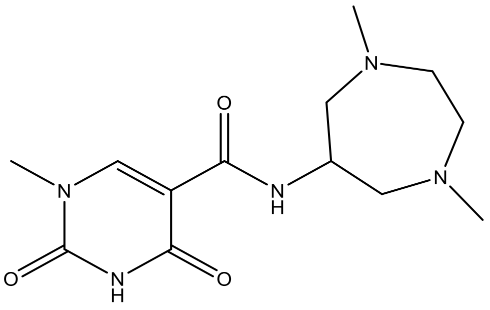<br>CN1CCN(C)CC(C1)NC(=O)C2=CN(C)C(=O)NC2=O
249	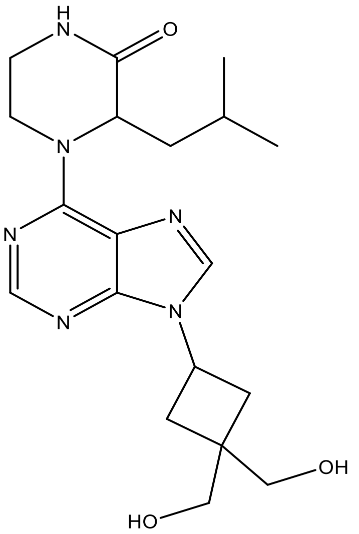<br>CC(C)CC1N(CCNC1=O)C=2N=CN=C3N(C=NC23)C4CC(CO)(CO)C4

250	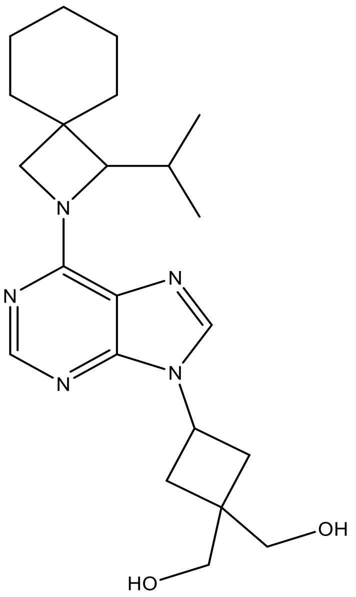<br>CC(C)C1N(CC21CCCCC2)C=3N=CN=C4N(C=NC34)C5CC(CO)(CO)C5
251	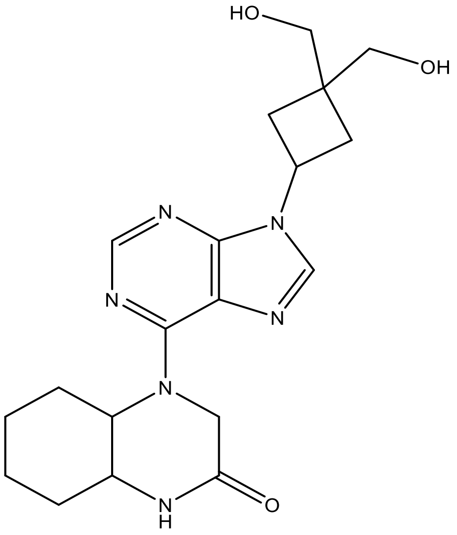<br>OCC1(CO)CC(C1)N2C=NC=3C(=NC=NC23)N4CC(=O)NC5CCCCC54
252	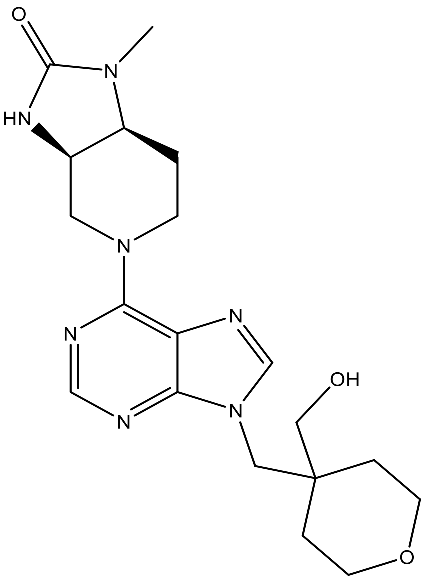<br>CN1[C@H]2CCN(C[C@H]2NC1=O)C=3N=CN=C4N(CC5(CO)CCOCC5)C=NC34
253	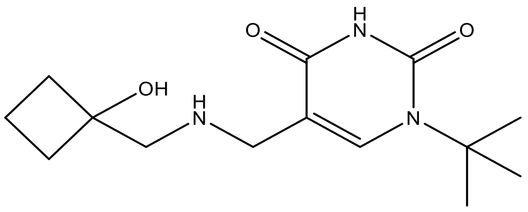<br>CC(C)(C)N1C=C(CNCC2(O)CCC2)C(=O)NC1=O

254	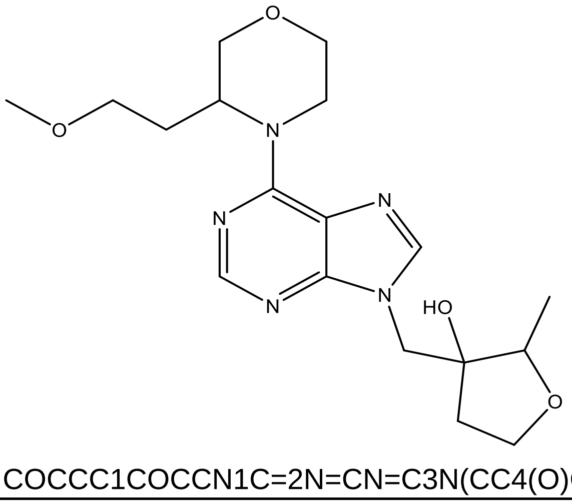<br>COCCC1COCCN1C=2N=CN=C3N(CC4(O)CCOC4C)C=NC23
255	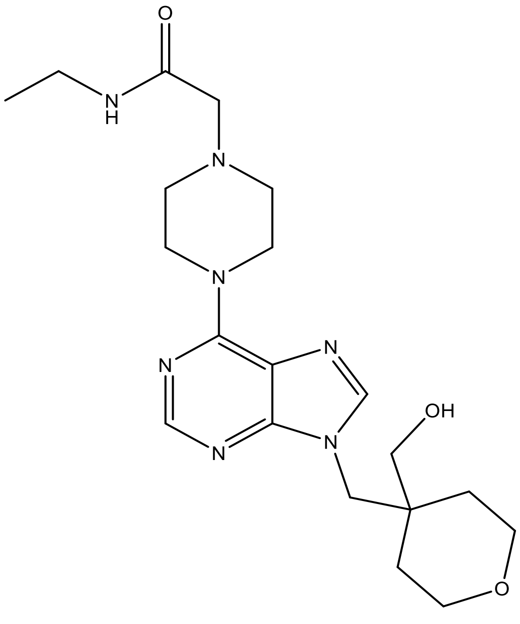<br>CCNC(=O)CN1CCN(CC1)C=2N=CN=C3N(CC4(CO)CCOCC4)C=NC23
256	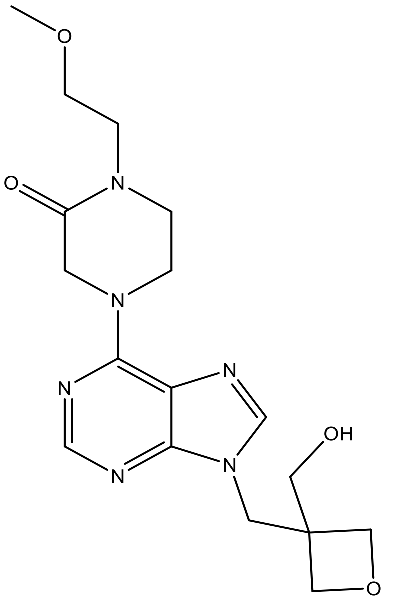<br>COCCN1CCN(CC1=O)C=2N=CN=C3N(CC4(CO)COC4)C=NC23
257	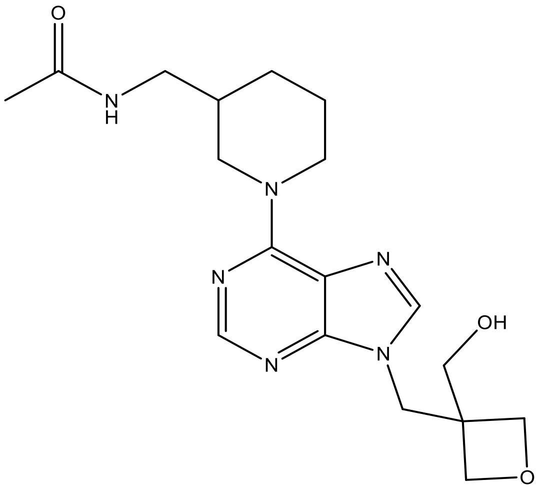<br>CC(=O)NCC1CCCN(C1)C=2N=CN=C3N(CC4(CO)COC4)C=NC23

258	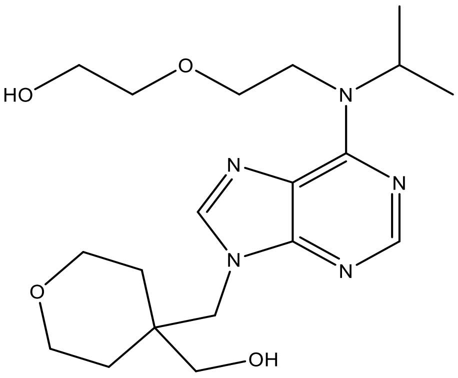<br>CC(C)N(CCOCCO)C=1N=CN=C2N(CC3(CO)CCOCC3)C=NC12
259	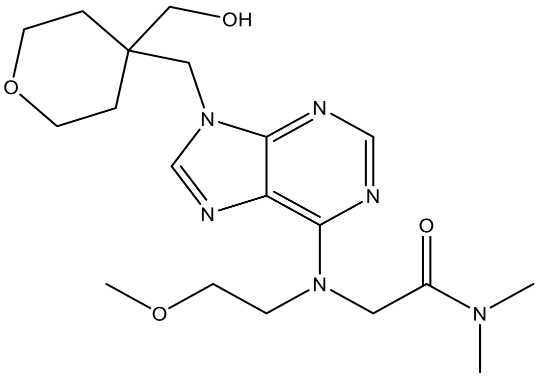<br>COCCN(CC(=O)N(C)C)C=1N=CN=C2N(CC3(CO)CCOCC3)C=NC12
260	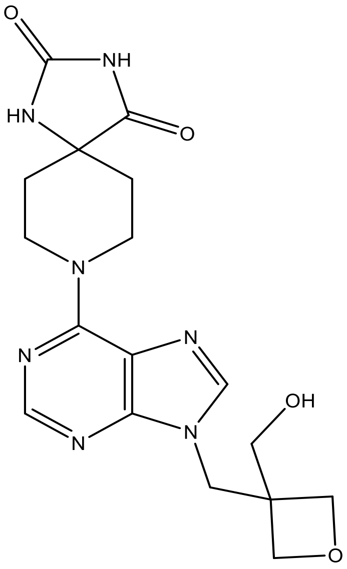<br>OCC1(CN2C=NC=3C(=NC=NC23)N4CCC5(CC4)NC(=O)NC5=O)COC1
261	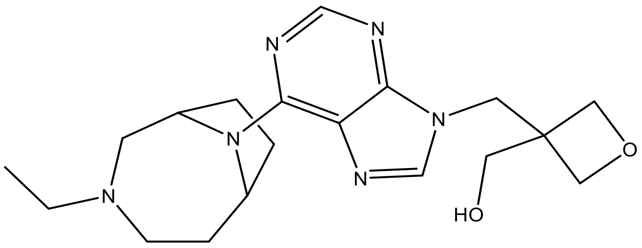<br>CCN1CCC2CCC(C1)N2C=3N=CN=C4N(CC5(CO)COC5)C=NC34
262	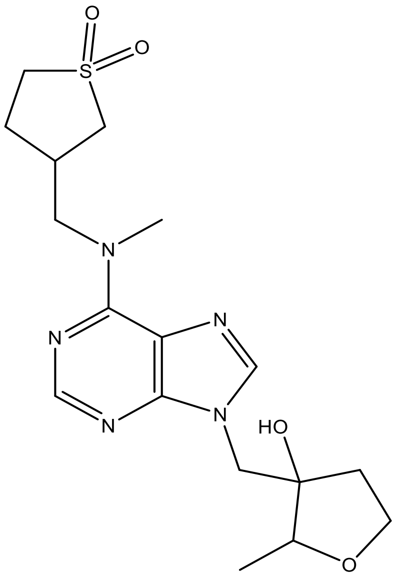<br>CC1OCCC1(O)CN2C=NC=3C(=NC=NC23)N(C)CC4CCS(=O)(=O)C4

263	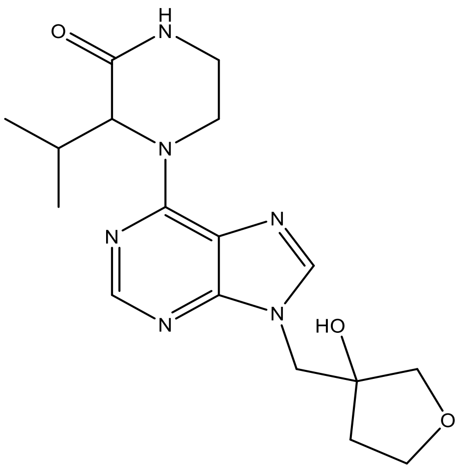 CC(C)C1N(CCNC1=O)C=2N=CN=C3N(CC4(O)CCOC4)C=NC23
264	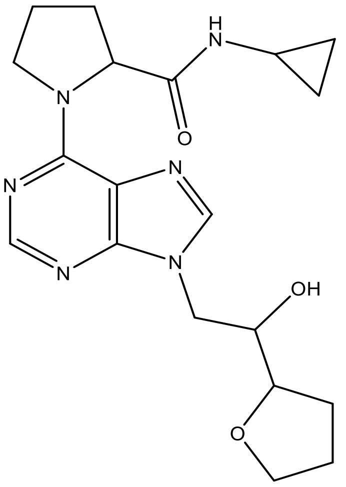 OC(CN1C=NC=2C(=NC=NC12)N3CCCC3C(=O)NC4CC4)C5CCCO5
265	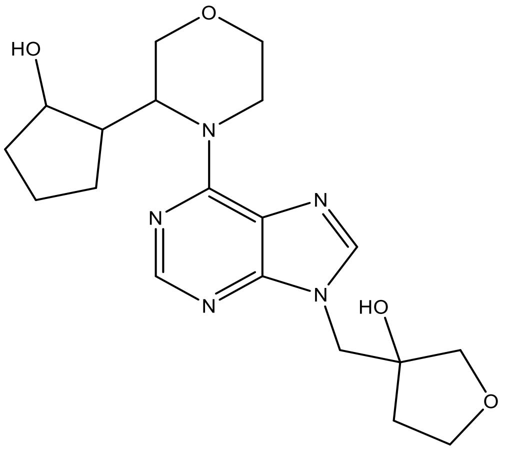 OC1CCCC1C2COCCN2C=3N=CN=C4N(CC5(O)CCOC5)C=NC34
266	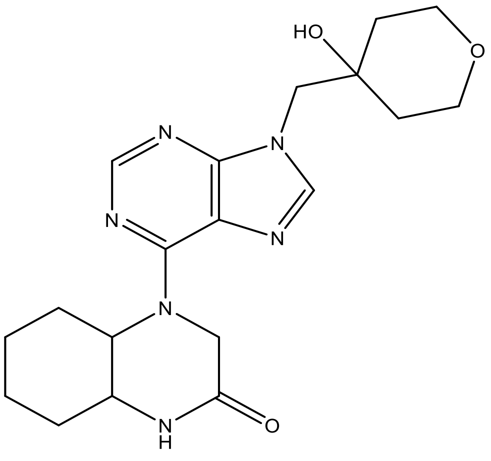 OC1(CN2C=NC=3C(=NC=NC23)N4CC(=O)NC5CCCCC54)CCOCC1

267	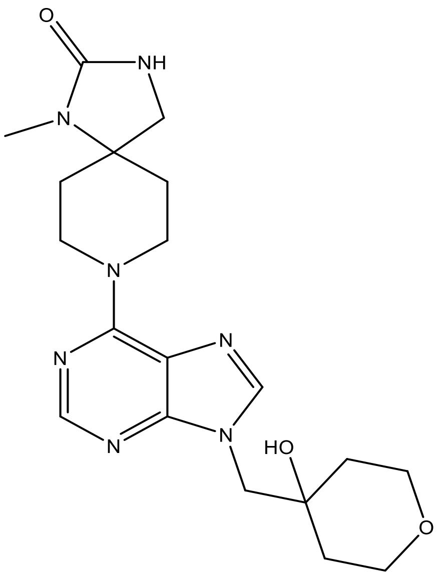<br>CN1C(=O)NCC21CCN(CC2)C=3N=CN=C4N(CC5(O)CCOCC5)C=NC34
268	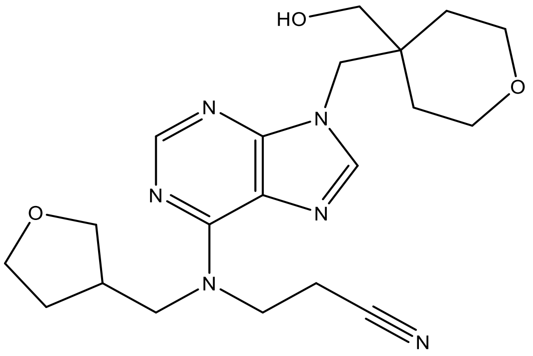<br>OCC1(CN2C=NC=3C(=NC=NC23)N(CCC#N)CC4CCOC4)CCOCC1
269	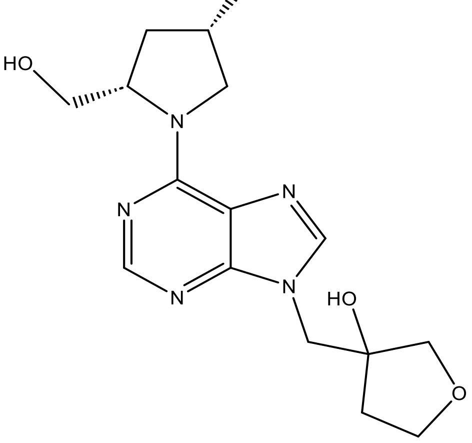<br>OC[C@@H]1C[C@H](F)CN1C=2N=CN=C3N(CC4(O)CCOC4)C=NC23
270	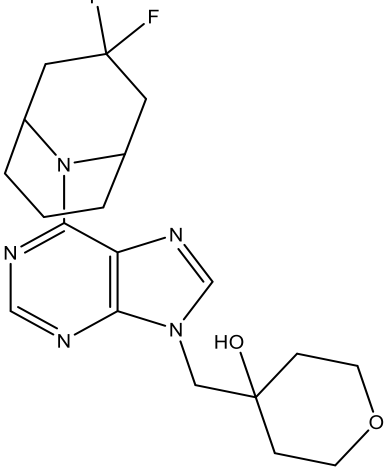<br>OC1(CN2C=NC=3C(=NC=NC23)N4C5CCCC4CC(F)(F)C5)CCOCC1

271	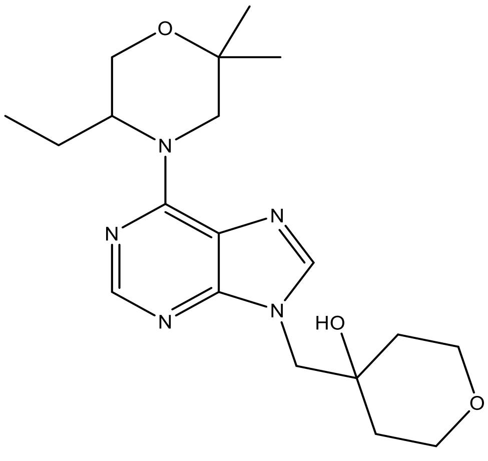<br>CCC1COC(C)(C)CN1C=2N=CN=C3N(CC4(O)CCOCC4)C=NC23
272	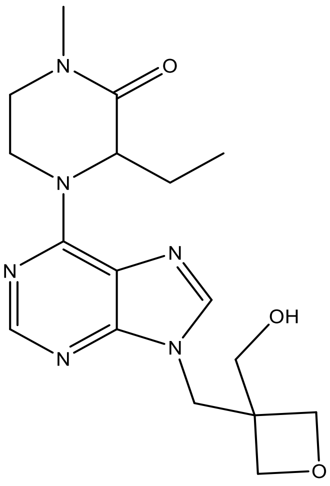<br>CCC1N(CCN(C)C1=O)C=2N=CN=C3N(CC4(CO)COC4)C=NC23
273	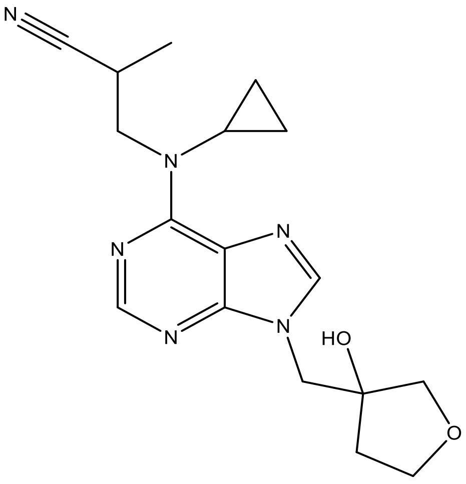<br>CC(CN(C1CC1)C=2N=CN=C3N(CC4(O)CCOC4)C=NC23)C#N
274	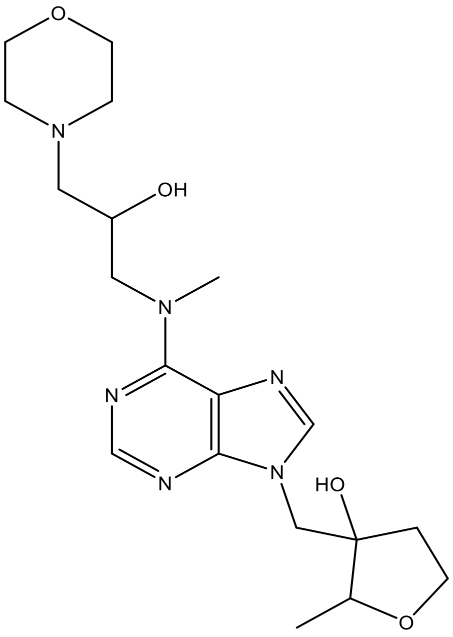<br>CC1OCCC1(O)CN2C=NC=3C(=NC=NC23)N(C)CC(O)CN4CCOCC4

275	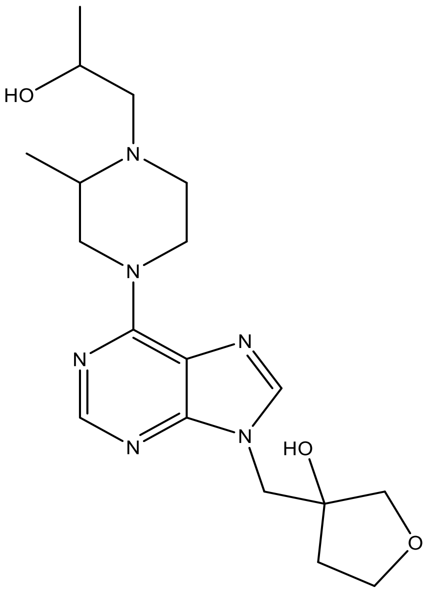 CC(O)CN1CCN(CC1C)C=2N=CN=C3N(CC4(O)CCOC4)C=NC23
276	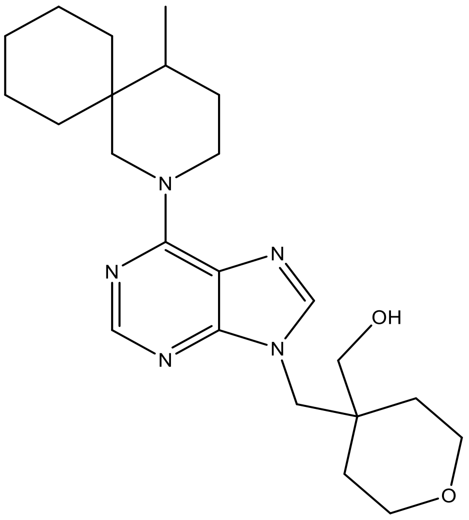 CC1CCN(CC21CCCCC2)C=3N=CN=C4N(CC5(CO)CCOCC5)C=NC34
277	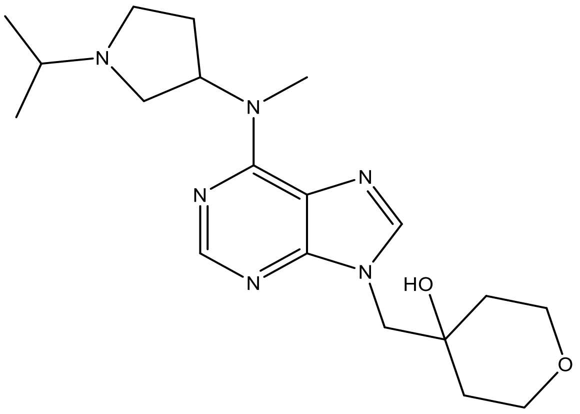 CC(C)N1CCC(C1)N(C)C=2N=CN=C3N(CC4(O)CCOCC4)C=NC23
278	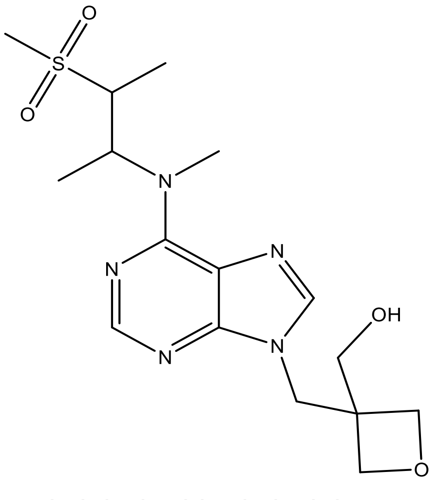 CC(C(C)S(=O)(=O)C)N(C)C=1N=CN=C2N(CC3(CO)COC3)C=NC12

279	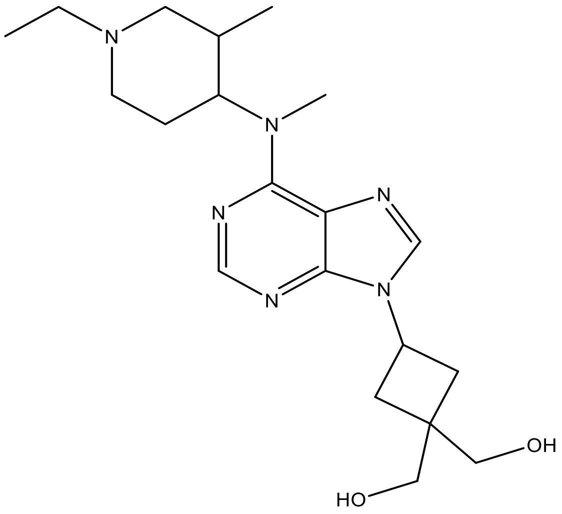<br>CCN1CCC(C(C)C1)N(C)C=2N=CN=C3N(C=NC23)C4CC(CO)(CO)C4
280	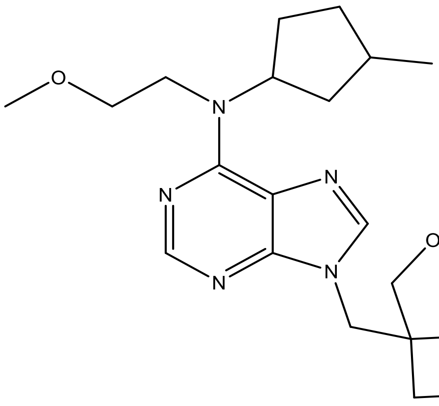<br>COCCN(C1CCC(C)C1)C=2N=CN=C3N(CC4(CO)COC4)C=NC23
281	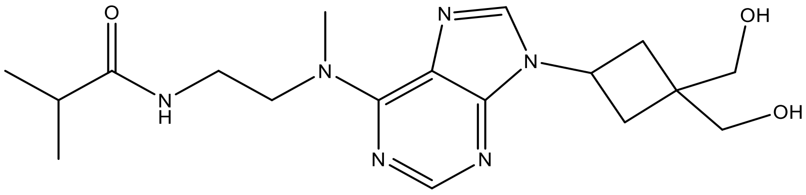<br>CC(C)C(=O)NCCN(C)C=1N=CN=C2N(C=NC12)C3CC(CO)(CO)C3
282	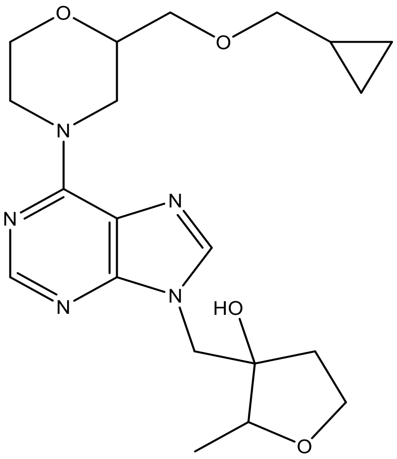<br>CC1OCCC1(O)CN2C=NC=3C(=NC=NC23)N4CCOC(COCC5CC5)C4
283	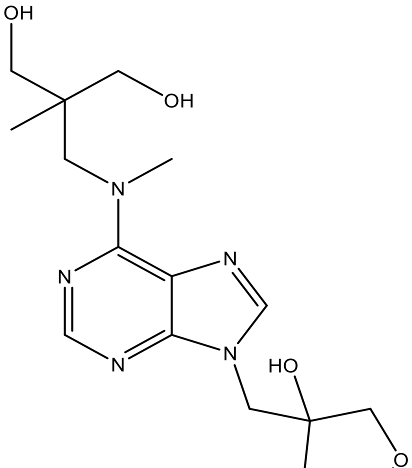<br>CN(CC(C)(CO)CO)C=1N=CN=C2N(CC3(O)CCOC3)C=NC12

284	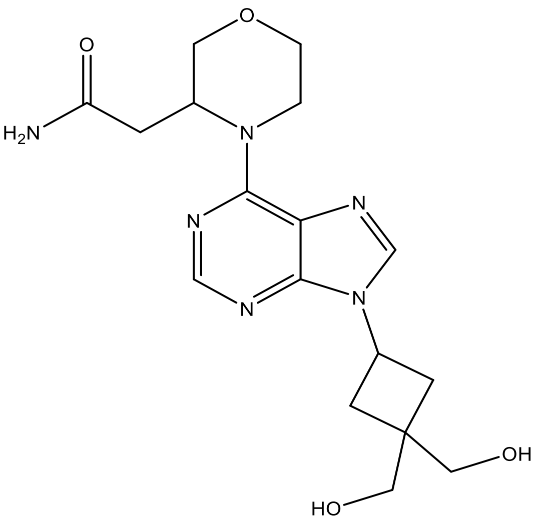<br>NC(=O)CC1COCCN1C=2N=CN=C3N(C=NC23)C4CC(CO)(CO)C4
285	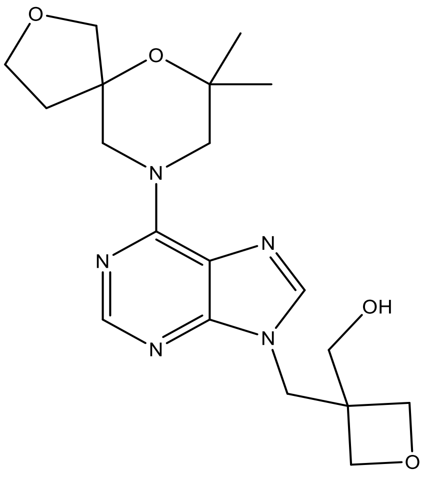<br>CC1(C)CN(CC2(CCOC2)O1)C=3N=CN=C4N(CC5(CO)COC5)C=NC34
286	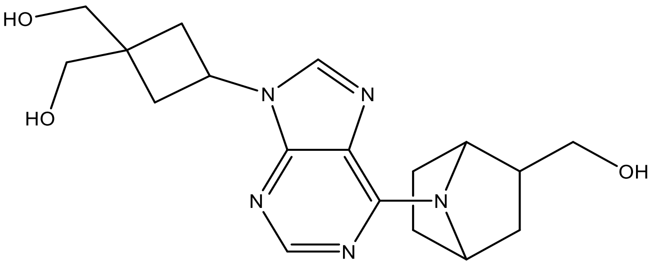<br>OCC1CC2CCC1N2C=3N=CN=C4N(C=NC34)C5CC(CO)(CO)C5
287	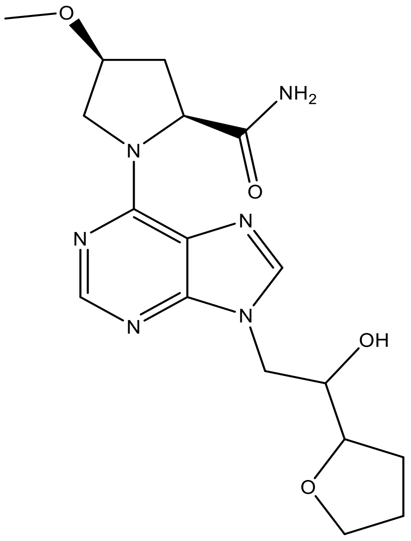<br>CO[C@H]1C[C@H](N(C1)C=2N=CN=C3N(CC(O)C4CCCO4)C=NC23)C(=O)N
288	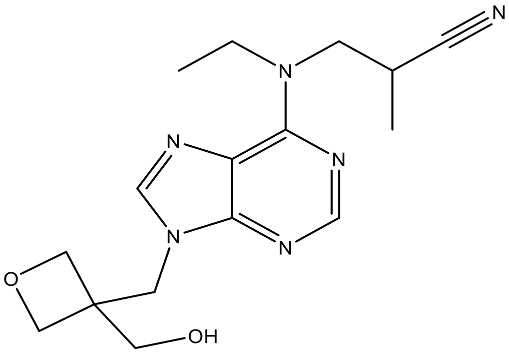<br>CCN(CC(C)C#N)C=1N=CN=C2N(CC3(CO)COC3)C=NC12

289	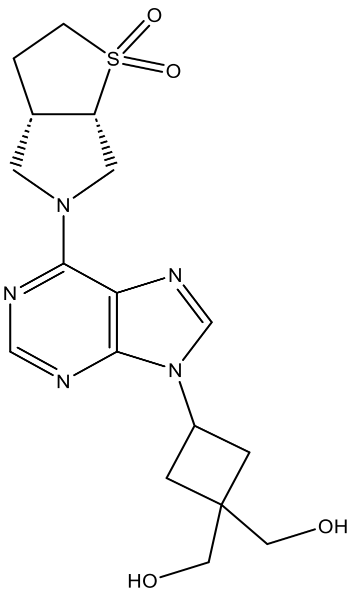 OCC1(CO)CC(C1)N2C=NC=3C(=NC=NC23)N4C[C@@H]5CCS(=O)(=O)[C@@H]5C4
290	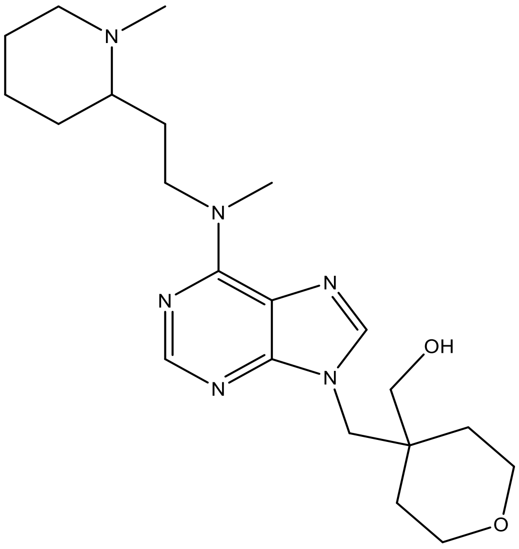 CN(CCC1CCCCN1C)C=2N=CN=C3N(CC4(CO)CCOCC4)C=NC23
291	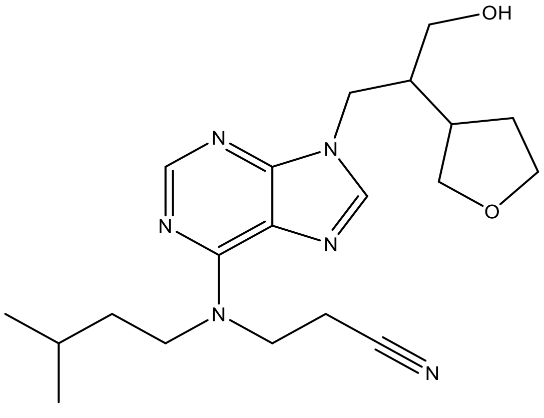 CC(C)CCN(CCC#N)C=1N=CN=C2N(CC(CO)C3CCOC3)C=NC12
292	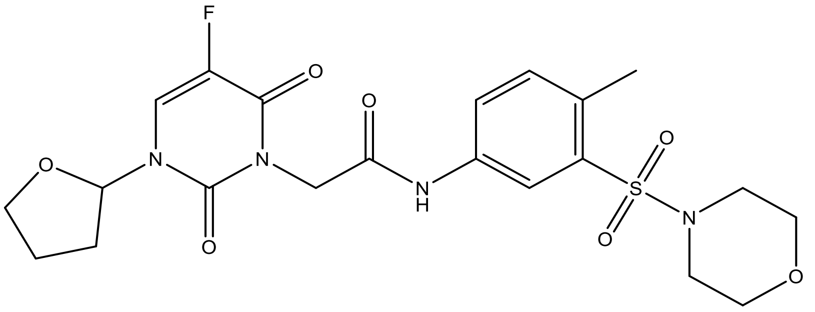 CC=1C=CC(NC(=O)CN2C(=O)C(F)=CN(C3CCCO3)C2=O)=CC1S(=O)(=O)N4CCOCC4

	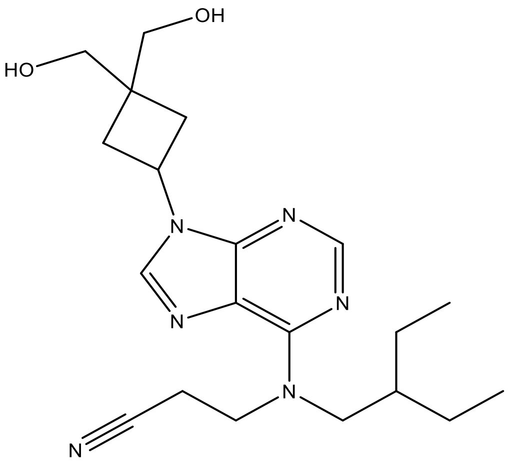
293	CCC(CC)CN(CCC#N)C=1N=CN=C2N(C=NC12)C3CC(CO)(CO)C3
	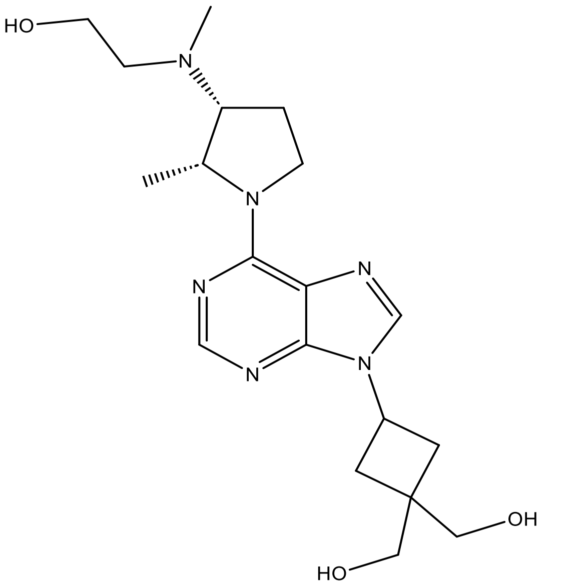
294	C[C@@H]1[C@@H](CCN1C=2N=CN=C3N(C=NC23)C4CC(CO)(CO)C4)N(C)CCO
	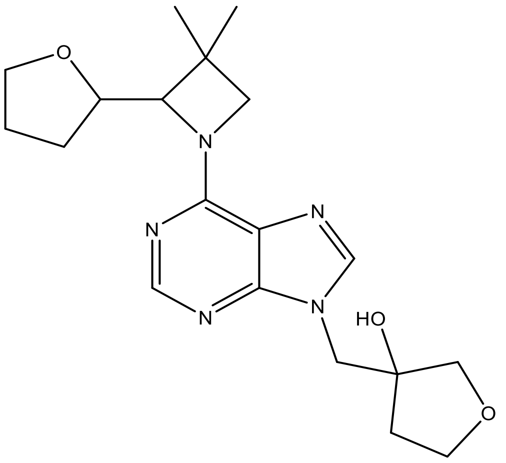
295	CC1(C)CN(C1C2CCCO2)C=3N=CN=C4N(CC5(O)CCOC5)C=NC34
	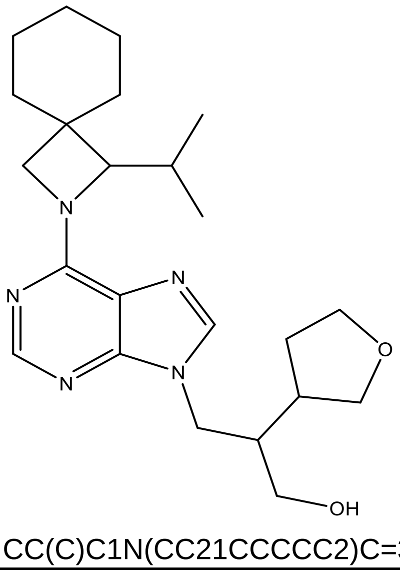
296	CC(C)C1N(CC21CCCCC2)C=3N=CN=C4N(CC(CO)C5CCOC5)C=NC34

	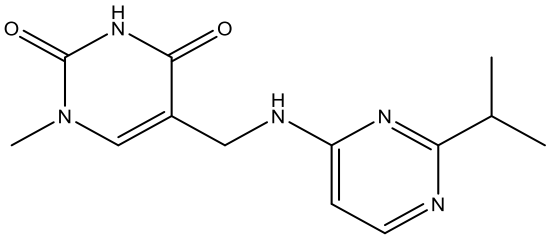
297	CC(C)C=1N=CC=C(NCC2=CN(C)C(=O)NC2=O)N1
	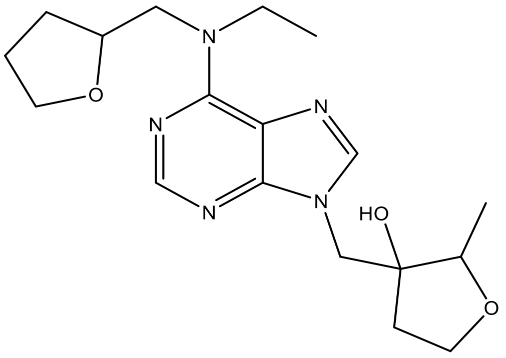
298	CCN(CC1CCCO1)C=2N=CN=C3N(CC4(O)CCOC4C)C=NC23
	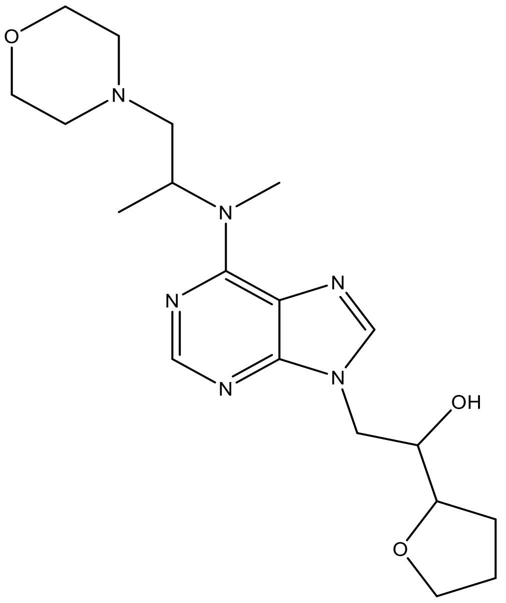
299	CC(CN1CCOCC1)N(C)C=2N=CN=C3N(CC(O)C4CCCO4)C=NC23
	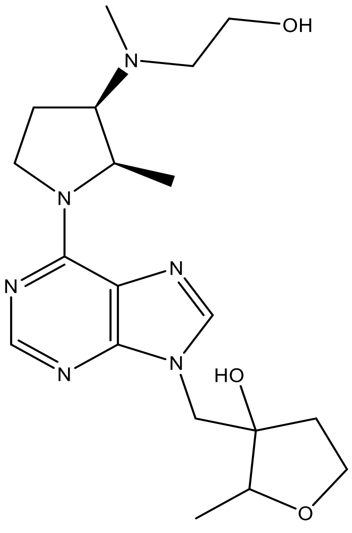
300	CC1OCCC1(O)CN2C=NC=3C(=NC=NC23)N4CC[C@H]([C@H]4C)N(C)CCO

301	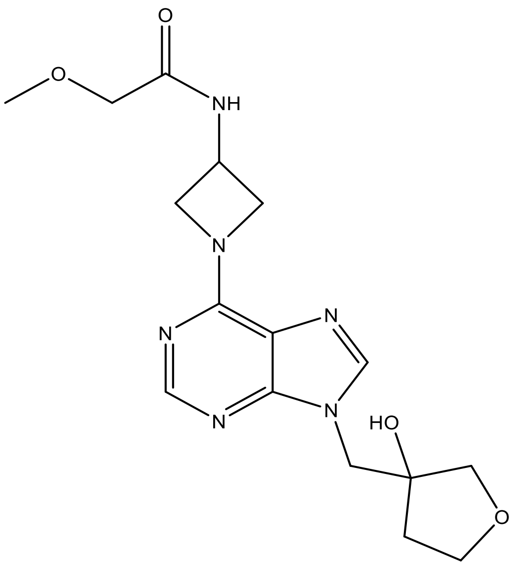 COCC(=O)NC1CN(C1)C=2N=CN=C3N(CC4(O)CCOC4)C=NC23
302	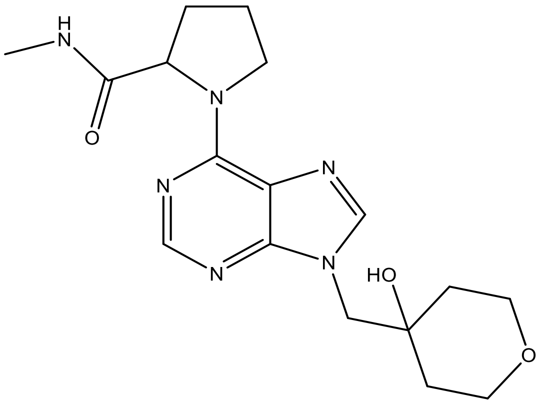 CNC(=O)C1CCCN1C=2N=CN=C3N(CC4(O)CCOCC4)C=NC23
303	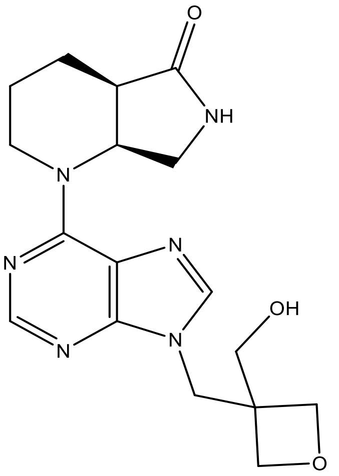 OCC1(CN2C=NC=3C(=NC=NC23)N4CCC[C@@H]5[C@H]4CNC5=O)COC1
304	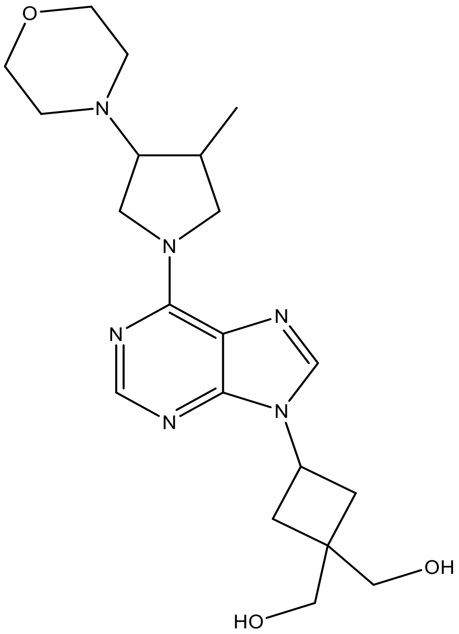 CC1CN(CC1N2CCOCC2)C=3N=CN=C4N(C=NC34)C5CC(CO)(CO)C5

305	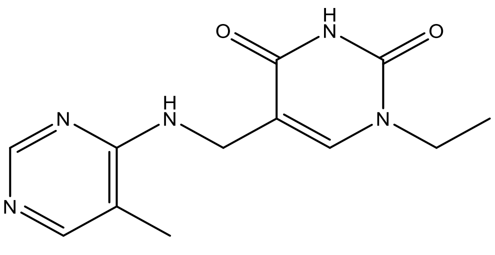<br>CCN1C=C(CNC=2N=CN=CC2C)C(=O)NC1=O
306	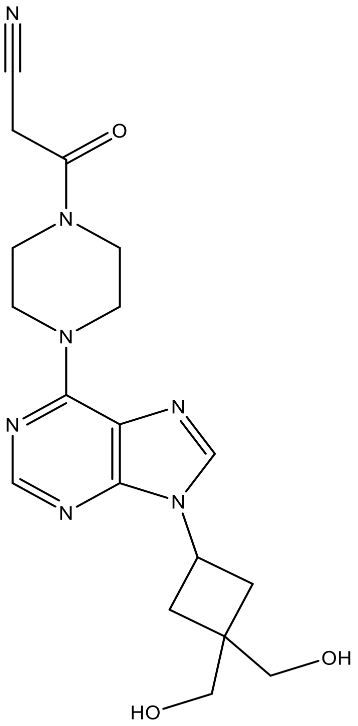<br>OCC1(CO)CC(C1)N2C=NC=3C(=NC=NC23)N4CCN(CC4)C(=O)CC#N
307	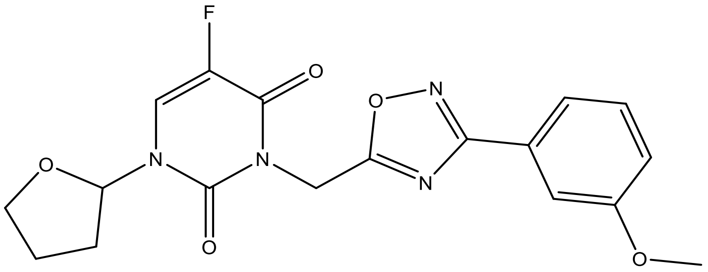<br>COC=1C=CC=C(C1)C2=NOC(CN3C(=O)C(F)=CN(C4CCCO4)C3=O)=N2
308	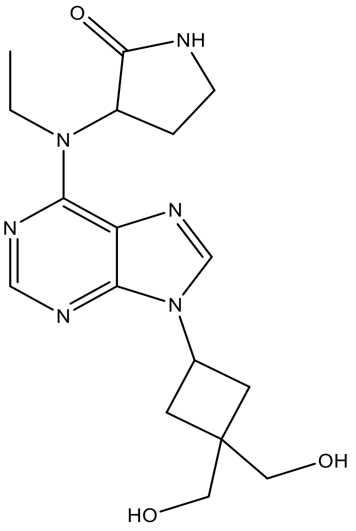<br>CCN(C1CCNC1=O)C=2N=CN=C3N(C=NC23)C4CC(CO)(CO)C4
309	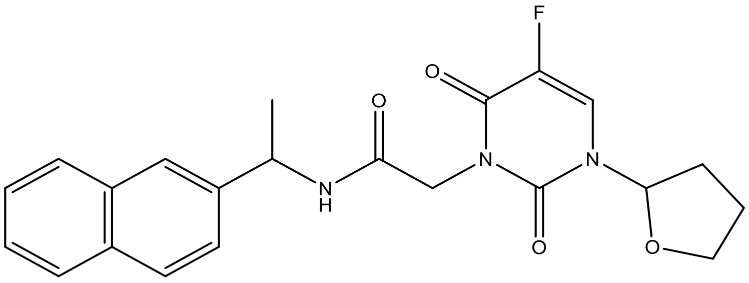<br>CC(NC(=O)CN1C(=O)C(F)=CN(C2CCCO2)C1=O)C=3C=CC=4C=CC=CC4C3

310	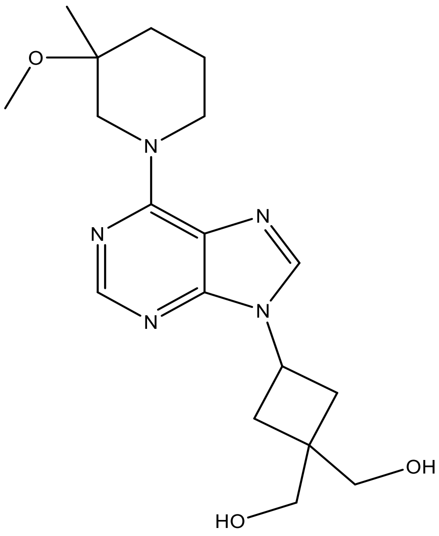 COC1(C)CCCN(C1)C=2N=CN=C3N(C=NC23)C4CC(CO)(CO)C4
311	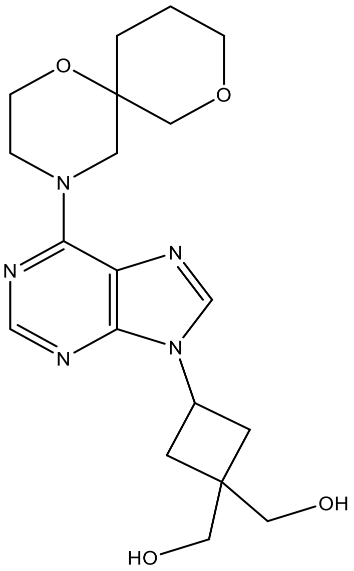 OCC1(CO)CC(C1)N2C=NC=3C(=NC=NC23)N4CCOC5(CCCOC5)C4
312	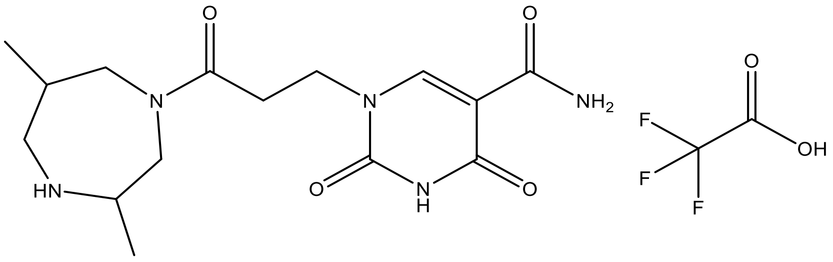 CC1CNC(C)CN(C1)C(=O)CCN2C=C(C(=O)N)C(=O)NC2=O.OC(=O)C(F)(F)F
313	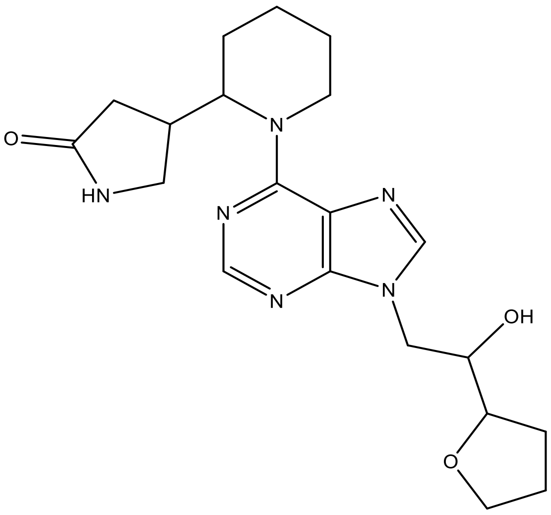 OC(CN1C=NC=2C(=NC=NC12)N3CCCCC3C4CNC(=O)C4)C5CCCO5

314	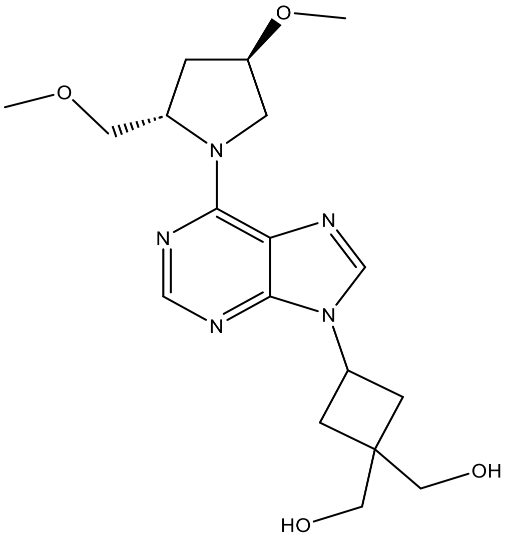 COC[C@@H]1C[C@H](CN1C=2N=CN=C3N(C=NC23)C4CC(CO)(CO)C4)OC
315	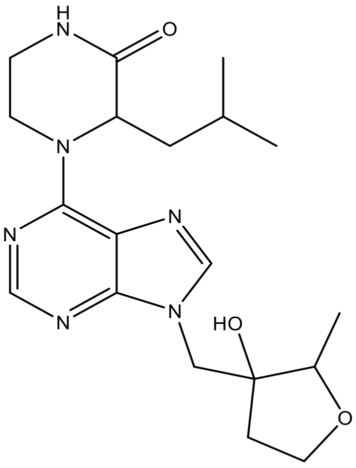 CC(C)CC1N(CCNC1=O)C=2N=CN=C3N(CC4(O)CCOC4C)C=NC23
316	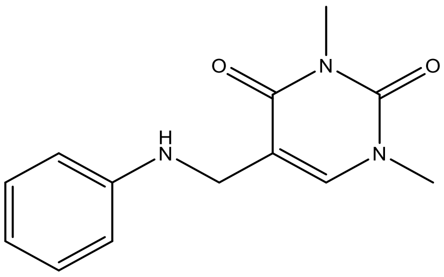 CN1C=C(CNC=2C=CC=CC2)C(=O)N(C)C1=O
317	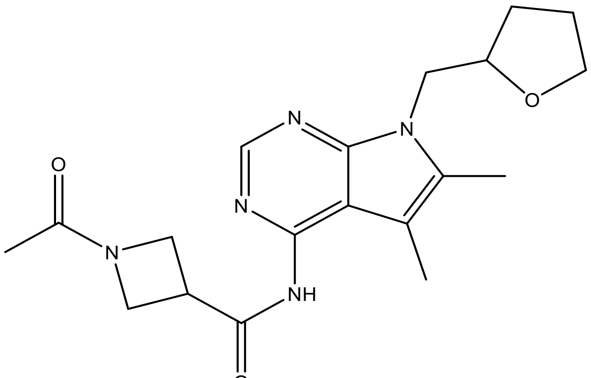 CC(=O)N1CC(C1)C(=O)NC=2N=CN=C3N(CC4CCCO4)C(C)=C(C)C23

318	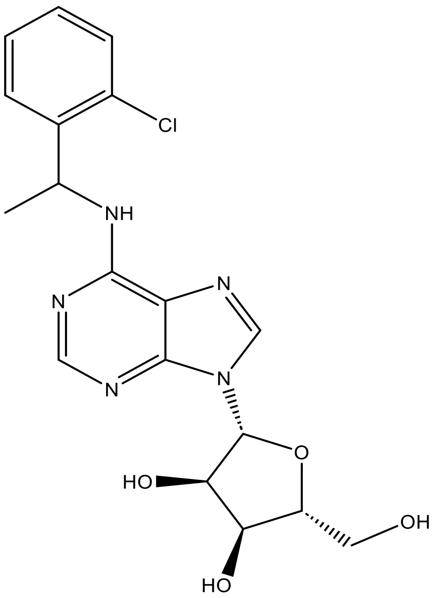<br>CC(NC=1N=CN=C2N(C=NC12)[C@@H]3O[C@H](CO)[C@@H](O)[C@H]3O)C=4C=CC=CC4Cl
319	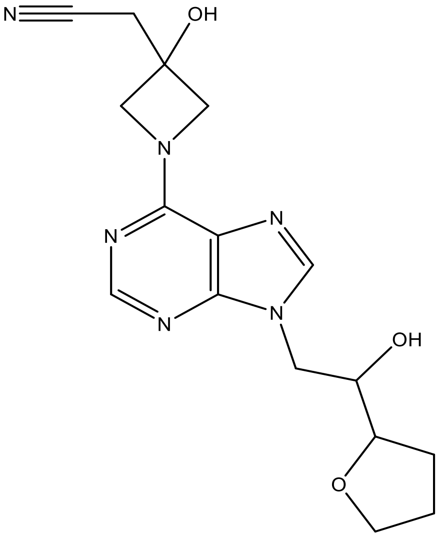<br>OC(CN1C=NC=2C(=NC=NC12)N3CC(O)(CC#N)C3)C4CCCO4
320	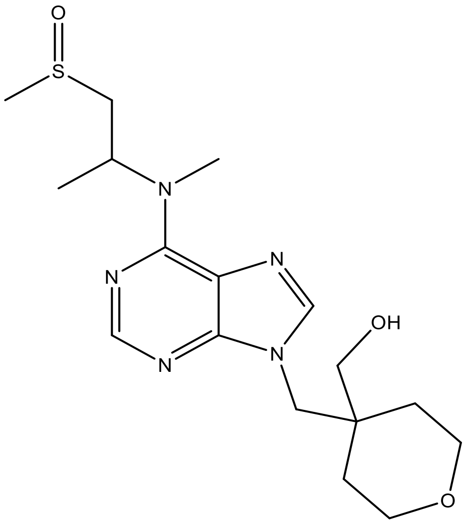<br>CC(CS(=O)C)N(C)C=1N=CN=C2N(CC3(CO)CCOCC3)C=NC12
