# Supplementary material for: FRET Assays for the Identification of C. albicans HSP90-Sba1 and Human HSP90α-p23 Binding Inhibitors
Source: Pharmaceuticals (Basel). 2024 Apr 17;17(4):516. doi: 10.3390/ph17040516 (PMC11053944; doi:10.3390/ph17040516)
Supplement: Supplementary file 1 [file pharmaceuticals-17-00516-s001.zip › Supplementary Material_ACI-Kd report_C. albicans HSP90-Sba1 binding.pdf]

AcuK - ACI Assessment

15/02/2024 15:45

Results: Confidence intervals of determined and true K_d with different confidence levels

Confidence levels

	**68.3%**	**95.5%**	**99.7%**
Confidence interval of determined K_d (ECI)	83.97-106.20 nM	72.85-117.31 nM	61.74-128.42 nM
Confidence interval of true K_d (ACI)	99.46-120.39 nM	93.37-127.97 nM	87.56-136.57 nM

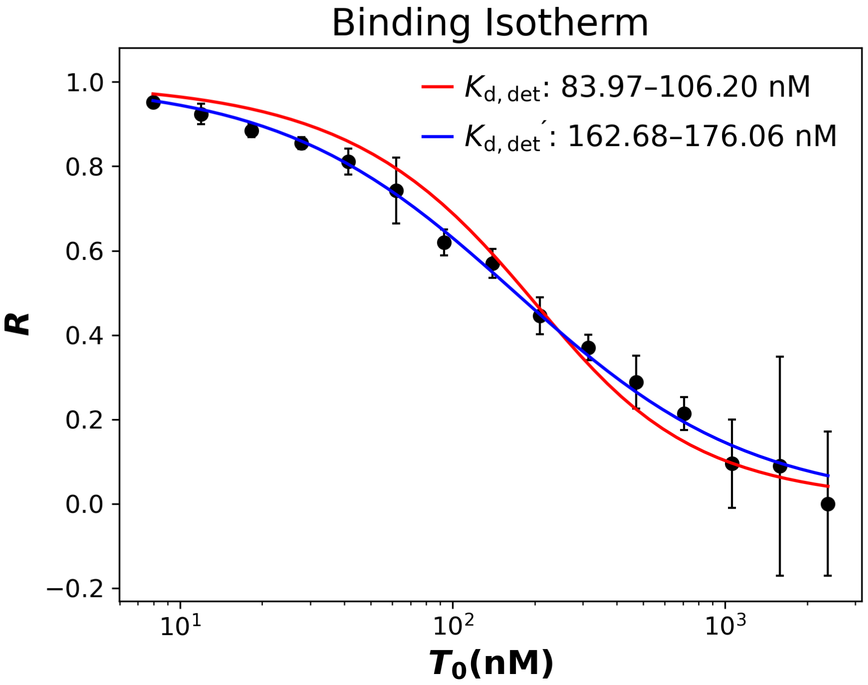

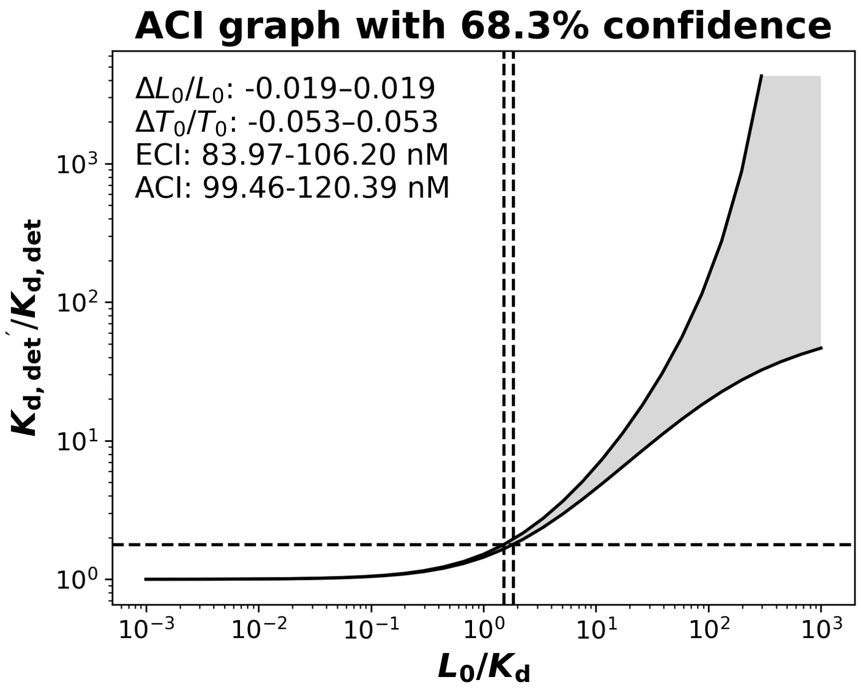

Input Data of Binding Isotherm (L_0= 183.0 nM)

T_0 (nM)	R Values	R RSD
2385.0	-4.44444445135694e-09	0.170952613643146
1590.0	0.089082256666667	0.259693952365186
1059.735	0.095233475555556	0.10454348255911
706.755	0.213277377777778	0.038984101679075
471.435	0.288606455555556	0.06264522986613
314.025	0.3702753	0.030514968000803
209.085	0.445797777777778	0.043786788671264
139.92	0.569947566666667	0.033908812723575
93.015	0.6193277	0.030973187905429
62.01	0.742837166666667	0.077809411755809
41.34	0.810896855555556	0.030760933293673
27.825	0.854907255555556	0.014508428912714

18.285	0.885011177777778	0.015921556226213
11.925	0.924100977777778	0.024133986517605
7.95	0.9520356	0.0099740948751
0.0	0.999999922222222	0.021520858365245

Analysis:

ECI ($K_{d,det}$): 83.97-106.20 nM

$K_{d,det}'/K_{d,det}$: 1.78

L_0/K_d: 1.52-1.84

ACI ($K_{d,true}$): 99.46-120.39 nM

$K_{d,det}/K_{d,true}$: 0.79-0.96
