# Supplementary material for: FRET Assays for the Identification of C. albicans HSP90-Sba1 and Human HSP90α-p23 Binding Inhibitors
Source: Pharmaceuticals (Basel). 2024 Apr 17;17(4):516. doi: 10.3390/ph17040516 (PMC11053944; doi:10.3390/ph17040516)
Supplement: Supplementary file 1 [file pharmaceuticals-17-00516-s001.zip › Supplementary Material_ACI-Kd report_human HSP90a-p23 binding.pdf]

AcuK - ACI Assessment

15/02/2024 15:49

Results: Confidence intervals of determined and true K_d with different confidence levels

	**Confidence levels**		
	**68.3%**	**95.5%**	**99.7%**
Confidence interval of determined K_d (ECI)	197.07-237.81 nM	176.70-258.18 nM	156.32-278.56 nM
Confidence interval of true K_d (ACI)	209.33-249.21 nM	196.25-266.1 nM	180.46-280.36 nM

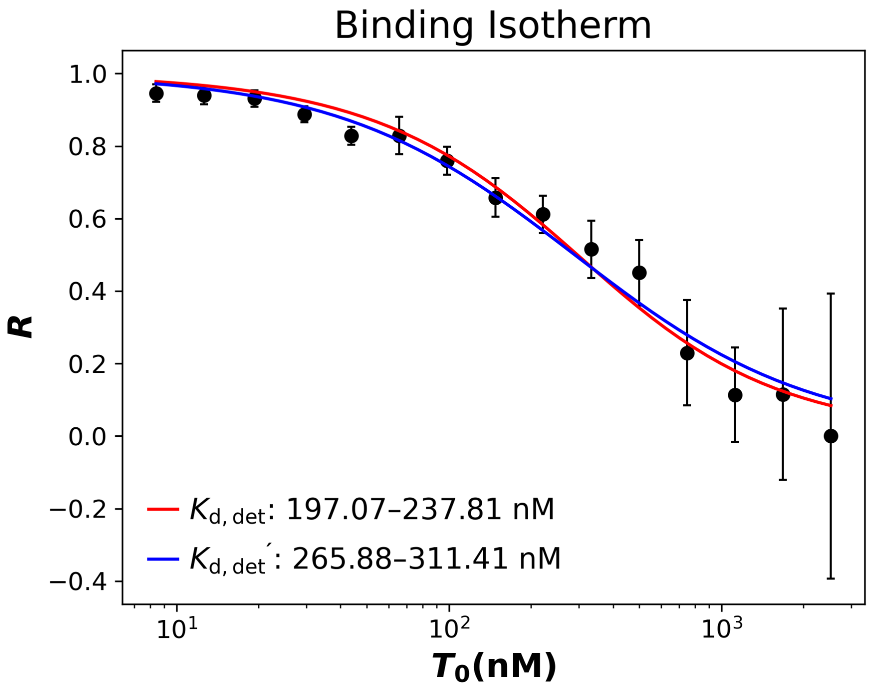

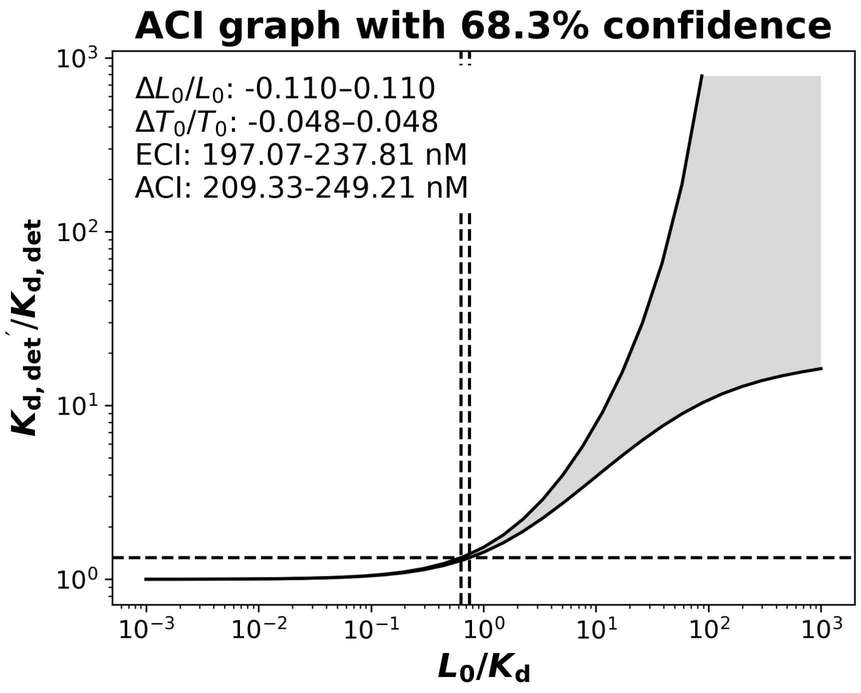

Input Data of Binding Isotherm

(L_0= 157.0 nM)

T_0 (nM)	R Values	R RSD
2523.0	-6.66666667549344e-09	0.393771158499173
1682.0	0.115033214444444	0.236295598586142
1121.053	0.113823836666667	0.129871134709478
747.649	0.229466982777778	0.145085681410565
498.713	0.4504173	0.090731633612911
332.195	0.515304177777778	0.078931639678993
221.183	0.611538577777778	0.052101069295692
148.016	0.657958811111111	0.053276848308774
98.397	0.759524333333333	0.038537633315728
65.598	0.829083388888889	0.051689305690115
43.732	0.828231977777778	0.025103764749873
29.435	0.887954333333333	0.022286682887209

AcuK - ACI Assessment

15/02/2024 15:49

19.343	0.931115488888889	0.022318519100595
12.615	0.939761822222222	0.024872457758472
8.41	0.9458468	0.02419472193248
0.0	0.987135366666667	0.007682458542518

Analysis:

ECI ($K_{d,det}$): 197.07-237.81 nM

$K_{d,det}'/K_{d,det}$: 1.33

L_0/K_d: 0.63-0.75

ACI ($K_{d,true}$): 209.33-249.21 nM

$K_{d,det}/K_{d,true}$: 0.87-1.04
